# Effects of diarrhea and antibiotic-induced microbial elimination on dynamic changes in fecal microbial communities and antibiotic resistance of Hu sheep lambs (*Ovis aries*)

**DOI:** 10.7717/peerj.21574

**Published:** 2026-07-31

**Authors:** Xi Li, Jiayi Jiang, Xianling Li, Guosheng Jian, Fengjun Li

**Affiliations:** 1 Institute of Ecology, China West Normal University, Nanchong, Sichuan, China; 2 College of Life Science, China West Normal University, Nanchong, Sichuan, China; 3 Key Laboratory of Southwest China Wildlife Resources Conservation (Ministry of Education), China West Normal University, Nanchong, Sichuan, China

**Keywords:** Hu Sheep, Intestinal microbiota, Diarrhea, Metagenome assembled genomes (MAGs), Antibiotic resistance genes (ARGs)

## Abstract

**Background:**

As a highly reproductive meat sheep breed in China, Hu sheep is an important economic group in ruminant animal breeding. However, research on its intestinal microbiomes under the background of diarrhea and antibiotic treatment remains relatively limited.

**Methods:**

This study investigated the intestinal microbiota of Hu sheep lambs in the preliminary stage of diarrhea (group DM), the late recovery stage of diarrhea (group DL), and the healthy stage (group H). Diseased individuals (groups DM and DL) were treated with a combination of Shuanghuanglian, Cefazolin, Lincomycin, and Dexamethasone (0.2 mL dosage). To characterize the intestinal microbiota, fecal samples were collected from all groups, and metagenomic sequencing was performed. Using metagenomic binning tools and co-assembly methods, we reconstructed 482 high-quality non-redundant metagenome assembled genomes (MAGs).

**Results:**

Among these MAGs, 70% belong to the phyla Bacillota, Bacteroidota, and Pseudomonadota, highly consistent with the typical structure of intestinal microbiota in ruminants. Functional annotation revealed that the genes encoding carbohydrate-active enzymes (CAZymes) are more abundant in Bacillota and Bacteroidota, which supports the degradation and energy metabolism functions of Hu sheep on fibrous feed. During the preliminary stage of diarrhea, the virulence genes carried by symbiotic bacteria such as Lachnospiraceae, Acutalibacteraceae and Bacteroidaceae were enriched. Although diarrhea symptoms alleviated during the late recovery stage of diarrhea, the combined use of multiple antibiotics led to the continuous enrichment of antibiotic resistance genes (ARGs) related to lincosamides and cephalosporins. The average abundance of cephalosporin-related ARGs in group DL was significantly higher than that in group DM and H, indicating a risk of residual ARGs. Microbial diversity analysis showed that there was no significant overall difference in MAGs between group DM and H, but both groups showed significant differences compared to group DL, suggesting that antibiotic driven clearance of sensitive bacteria is the core driving force. Moreover, our study shows that the abundance of the zoonotic pathogens *Barnesiella* and *Campylobacter* significantly increased in the diarrhea group (*p* <  0.05), and they carry 567 and 382 virulence genes, respectively. Their pathogenicity is regulated by the dynamic changes in the host intestinal microbiota.

**Conclusions:**

This study not only expands the genomic database of ruminant intestinal microorganisms but also provides a key theoretical basis for formulating intestinal microecological regulation strategies and optimizing diarrhea treatment regimens for Hu sheep.

## Introduction

The intestinal microbiota is the core regulator of animal homeostasis, and its community structure and function are directly related to host nutritional metabolism, immune defense, and physiological functions (Yang et al., 2024a). In captivity, ruminants rely on intestinal microbiota to decompose recalcitrant carbohydrates (Chen, Luo & Yan, 2021) such as cellulose and hemicellulose to produce short chain fatty acids (SCFAs), which provide energy for their hosts (Zhao et al., 2023a). Meanwhile, beneficial microorganisms such as *Bifidobacterium* and *Lactobacillus* can competitively inhibit the colonization of pathogenic bacteria, regulate mucosal immune barrier function, and reduce the occurrence of intestinal inflammation (Chen, Luo & Yan, 2021). Once the microbial community is imbalanced, such as a decrease in microbial diversity and an enrichment of pathogenic bacteria, it can lead to a decrease in host digestive efficiency, immune dysfunction, and an increased risk of diseases such as diarrhea and enteritis (Ma et al., 2022). Many studies have shown that the productivity of ruminants is strongly influenced by the intestinal microbiota (Zhong et al., 2022b; Yao et al., 2024; Zhang, Ma & Qi, 2025). For livestock, dietary intervention is a widely used strategy to modulate gut ecology: for instance, liquid whey-supplemented diets have been shown to reshape gut microbiota composition (*e.g.*, increasing *Lactobacillus* abundance) and enhance intestinal barrier function (D’Alessandro et al., 2022; Mhalhel et al., 2025). Therefore, analyzing the association mechanism between the intestinal microbiota and host health is the key foundation for ensuring the productive efficiency of captive animals.

Hu sheep is a unique and excellent breed of meat sheep in China, classified as short fat tail sheep (Sun et al., 2023). Due to its high reproductive rate (2–3 lambs per year, 2–3 lambs per litter) (Yue, 1996), fast growth rate, heat and moisture resistance, early maturity, and good meat quality (Zhao et al., 2024), it has become the core breed for large-scale meat sheep breeding in East and North China, and was introduced to the western region for breeding in the 1990s (Yuan et al., 2025; Liu et al., 2025). In recent years, with the promotion of intensive breeding models, the scale of Hu sheep breeding has continued to expand, with an annual output of over ten million sheep nationwide (Wang et al., 2024a). However, high-density breeding has also increased the risk of disease transmission (Zhao et al., 2023b).

In the breeding of Hu sheep, digestive related diseases are the main problems restricting their healthy growth, and common diseases include lamb diarrhea and chronic enteritis (Wang et al., 2019). In Hu sheep and East Friesian sheep, weaned animals exhibited poor disease resistance and were highly susceptible to diseases such as diarrhea (Yao et al., 2024). In addition, the intestinal microbiota of Hu sheep has more pathways related to nutrient metabolism than that of East Friesian sheep, suggesting that intestinal microbiota is the main driving factor for the differences in disease resistance phenotypes between Hu sheep and East Friesian sheep (Zhong et al., 2022a). Current research suggests that the occurrence of such diseases is closely related to feed management, environmental stress, and intestinal microbiota disorders (Wang et al., 2019). For example, Zhong et al. (2022a) has shown that 25% of lambs suffer from diarrhea and the highest incidence rate is observed in lambs of 1–2 months old. It is also found that the bacterial diversity of lambs increases in the first few days after weaning, and the composition of many dominant groups changes in the intestinal tract of lambs. Sheep suffering from diarrhea exhibit symptoms such as dehydration, sudden weight loss, and growth retardation, directly causing economic losses (Mariano et al., 2018; El-gbily et al., 2025). Data indicate that the costs related to diseases such as enteritis and pneumonia in animal husbandry, including deaths and productivity losses, range from $10 million to $29 million annually, and the costs are increasing every year (Yao et al., 2024).

Antibiotics are the preferred clinical intervention for the treatment of diarrhea in captive ruminants (Van Boeckel et al., 2015; Du et al., 2023; Davies, Hyde & Lovatt, 2023). For example, in a study of 325 sheep flocks and 119 goat flocks in Greece, Lianou & Fthenakis (2022) found that the annual incidence rate of diarrhea in lambs was 9.6% and 13.7% respectively and the treatment of diarrhea in all lambs involved the use of antibiotics. Currently, commonly used drugs for ruminant animals with diarrhea include beta lactams (such as penicillin and cephalosporins), lincomsamides (such as lincomycin), and tetracyclines (such as oxytetracycline) antibiotics. In some cases, corticosteroids (such as dexamethasone) are also used in combination to alleviate inflammatory reactions (Ventola, 2015). For example, in a related study conducted in Canada, Moon et al. (2010) collected information from 49 sheep herds and reported that penicillin and oxytetracycline were the two most commonly used antibiotics (Moon et al., 2010).

Antibiotics are widely used in the clinical practice of captive animals to quickly control the disease (Subramaniam & Girish, 2020; Hou et al., 2022; Xiao, Li & Sun, 2023), but there are obvious irregularities in the current use of antibiotics in captive animals. Numerous studies have shown that long-term or excessive use of antibiotics in captive animals can disrupt the stability of intestinal microbiota, increase the risk of subsequent infections, and induce the production of antibiotic resistance genes (ARGs) in intestinal microbiota (Kim et al., 2021; Caffarena et al., 2021; Du et al., 2023). In addition, multiple studies have shown that antibiotic resistant bacteria can contaminate the environment through feces, spread through the food chain or environmental media, and pose a threat to public health safety (Xiao, Li & Sun, 2023). This is an important risk factor for antibiotic resistance (Davies, Hyde & Lovatt, 2023).

The traditional methods for studying intestinal microbiota in diarrheal sheep include isolation and culture, 16S rRNA and metagenomic sequencing. Metagenomic sequencing technology has significant advantages in analyzing the composition and function of intestinal microbiota compared to traditional microbial research methods (Tanca et al., 2017; Cholewińska et al., 2020; Wang et al., 2024b). By high-throughput sequencing and assembly, metagenomic assembly genomes (MAGs) can be obtained, which can not only analyze microorganisms in the community without bias, but also deeply explore the functional genes of microorganisms, such as Carbohydrate-active enzymes (CAZymes), Virulence Factors of Pathogenic Bacteria (VFDB), and Antibiotic Resistance Genes (ARGs). Multiple studies have utilized metagenomic technology to accurately locate the host microbiota of resistance genes in ruminants (Stewart et al., 2019; Albakri, Bouqellah & Shabana, 2020; Chang et al., 2020; Glendinning et al., 2021; Xie et al., 2021; Cheng et al., 2022; Zhang et al., 2022; Shi et al., 2023), which have clarified the transmission mechanism of resistance genes, and provide more comprehensive and accurate technical support for revealing the impact of disease status and drug intervention on intestinal microbiota genome and resistance. So far, studies on the impact of antibiotic administration on the intestinal microbiome and the ARGs in Hu sheep are not extensively conducted.

This study aims to characterize fecal microbiome dynamics using metagenomics in Hu sheep lambs during the preliminary and late recovery stages of diarrhea, as well as in healthy individuals. By analyzing the basic characteristics, functional gene differences, and antibiotic resistance gene distributions of the MAGs, we seek to uncover how disease states and antibiotic interventions shape intestinal microbial genomes, functional potential, and drug resistance of Hu sheep lambs. This research addresses critical gaps in Hu sheep intestinal microbiome research, providing a scientific basis for diarrhea prevention, precision antibiotic use, and sustainable Hu sheep production.

## Materials and Methods

### Sample collection

Fecal samples were collected from Chinese Hu sheep (*Ovis aries*) in YueXi County, Liangshan, Sichuan Province, China, and divided into three groups: the preliminary stage of diarrhea (group DM), the late recovery stage of diarrhea (group DL), and the healthy stage (group H) (Table S1). All lambs were approximately two months old and reared under captive management. Each composite sample was a triplicate of subsamples collected from the same individual. During the sampling period, each sheep was kept individually in a separate pen. Fresh feces were collected from the central portion of the fecal mass using sterile PE gloves to avoid environmental contamination as much as possible. Healthy lambs (group H) were vaccinated regularly, whereas diseased individuals (groups DM and DL) were treated with a combination of Shuanghuanglian, Cefazolin, Lincomycin, and Dexamethasone (0.2 mL dosage) during their diarrhea. The treatment protocol was determined based on veterinary clinical experience for the management of diarrhea in the study animals. Specifically, fecal samples for group DM were collected 2 days following the initial dose of the antibiotic combination therapy, corresponding to the preliminary diarrhea stage when animals exhibited early clinical signs of diarrhea. In contrast, samples for group DL were collected 14 days following the initial dose of the same combination therapy, representing the late recovery stage where animals showed significant alleviation of diarrhea symptoms and approached clinical recovery. The experimental procedures and protocol of this study were approved by the Ethics Committee of the College of Life Sciences, China West Normal University (approval number: 2025LLSC0097).

### Metagenomic data preprocessing

Metagenomic sequencing of samples was carried out by Novogene Co., Ltd. To obtain clean data for subsequent analysis, raw data from the Illumina sequencing platform were processed by removing low-quality reads, reads with ≥10 bp N bases, and adapter-contaminated reads. The KneadData workflow (https://github.com/biobakery/kneaddata) was used for quality control of raw sequencing reads. Adapter sequences, low-quality reads (Phred score < 20), along with host-derived sequences were trimmed using Trimmomatic (v0.39) (Bolger, Lohse & Usadel, 2014). We removed host contamination by aligning clean reads against the sheep reference genome (*Ovis aries*, ARS-UI_Ramb_v3.0) by using Bowtie2 (v2.4.5) (Langmead & Salzberg, 2012), with the parameters: *–un-conc*.

### Genomic assembly and binning strategies

Single-sample assemblies were generated using MEGAHIT (v1.2.9) (Li et al., 2016), setting the minimum contig length to 1,000 bp. To enhance recovery of low-abundance genomes, co-assembly was performed on three groups respectively. Binning of metagenomes was performed by three tools implemented in metaWRAP (v1.3.2) (Uritskiy, Di Ruggiero & Taylor, 2018): concoct (Alneberg et al., 2014), maxbin2 (Wu, Simmons & Singer, 2016), and metabat2 (Kang et al., 2015), followed by bin refinement with completeness more than 80% and contamination less than 10% *via* the metaWRAP bin_refinement module.

### Genome bin refinement and deduplication

The dRep (v3.6.2) (Olm et al., 2017) dereplicate module with the option: -sa 0.99 -nc 0.30 -comp 80 -con 10 was used to filter the refined bins. Initial bins were dereplicated at a stringent 99% average nucleotide identity (ANI) threshold to eliminate highly identical genomes. Afterwards, a less strict 95% ANI threshold was also applied to define species-level genome bins (SGBs). Genome quality metrics, including both completeness and contamination, were assessed by CheckM workflow (v1.2.3) (Parks et al., 2015) .

### Taxonomic assignment and MAG abundance profiling

The classify_wf module in GTDB-Tk (v2.4.1) (Chaumeil et al., 2022) was used to perform species annotation with reference to the Genome Taxonomy Database (GTDB vr226) database. Pairwise ANI values among MAGs were also calculated with GTDB-Tk. The taxonomy mappings were exported through the scripts provided by GTDB-Tk, and taxonomic labels were assigned at the lowest confidently resolved rank (from phylum to species). Phylogenetic tree of MAGs was generated by GTDB-Tk. Accordingly, GTDB nomenclature was adopted as the primary taxonomic framework throughout the entire manuscript and the corresponding NCBI synonyms were provided in parentheses at the first occurrence of each major phylum name. To determine the relative abundance of each genome in different samples, the coverage analysis of the metagenomic assembled genomes (MAGs) was performed using genome function of the CoverM (v 0.7.0) (Aroney et al., 2025), followed by calculating the coverage between the metagenomic sequencing data and the assembled genomes.

### Functional annotation of metagenome-assembled genomes

Through Prodigal (v2.6.3) (Hyatt et al., 2010), protein-coding genes in MAGs were predicted, followed by clustering into non-redundant sets at 99%, 95%, and 90% amino acid identity using CD-HIT (v4.8.1) (Fu et al., 2012). Based on the non-redundant gene set, SALMON (v1.10.3) (Patro et al., 2017) calculated the expression abundance of each gene in each sample. Functional annotations were obtained by aligning non-redundant genes to several database, namely UniProt database (Apweiler et al., 2004), Virulence Factors of Pathogenic Bacteria Database (VFDB) (Liu et al., 2022), Structured ARG reference database (SARG) (Yang et al., 2016), Microbial Antibacterial Biocide and Metal Resistance Genes Database (BacMet) (Pal et al., 2014), Pathogen-Host Interaction Database (PHI) (Winnenburg et al., 2006), along with Carbohydrate-active enzymes (CAZymes) (Cantarel et al., 2009) *via* DIAMOND (v2.1.10, *E*-value < 1e−5, Identity ≥ 50%, Coverage ≥ 70%) (Buchfink, Xie & Huson, 2015). Antibiotic resistance genes (ARGs) were annotated using the Resistance Gene Identifier (RGI) v6.0.3 software with default parameters against the Comprehensive Antibiotic Resistance Database (CARD) for resistome prediction from protein sequences (Alcock Brian et al., 2020). Functional annotations of orthologous groups in the eggNOG database were conducted using eggNOG-Mapper (v2.1.12) (Cantalapiedra et al., 2021). Annotations of KEGG orthologs (KOs) were performed using KofamKOALA (v1.3.0) (Aramaki et al., 2020), and rRNA genes were predicted using Barrnap (v0.9) (Loman, 2017). Data processing was performed using dplyr/tidyr, and visualization was carried out with ggplot2 (Wilkinson, 2011) in combination with ColorBrewer, to reveal the distribution characteristics of resistance genes under different physiological states and their associations with therapeutic medications. Visualization analysis was exhibited using the R ggplot2 (v4.3.1) (Wilkinson, 2011) and the OmicStudio tools (Lyu et al., 2023) (https://www.omicstudio.cn/tool).

## Results

### Reconstruction of MAGs from fecal metagenomes of Hu sheep

**Figure 1:**
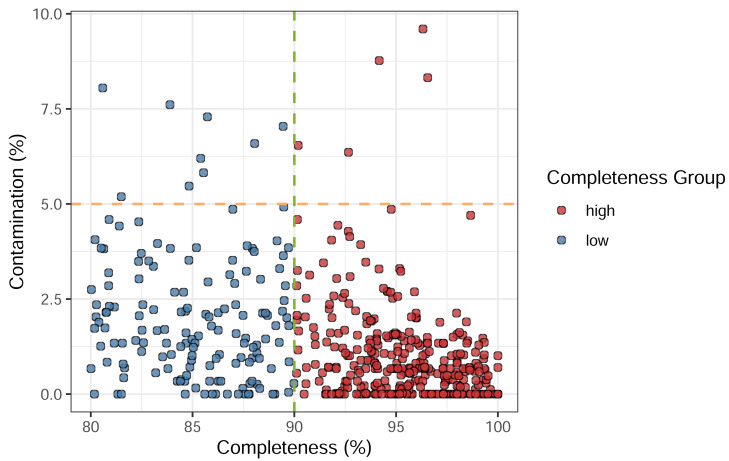
The distribution of completeness and contamination of MAGs. The red point represents high-quality MAGs with completeness > 90% and contamination <5%.

**Figure 2:**
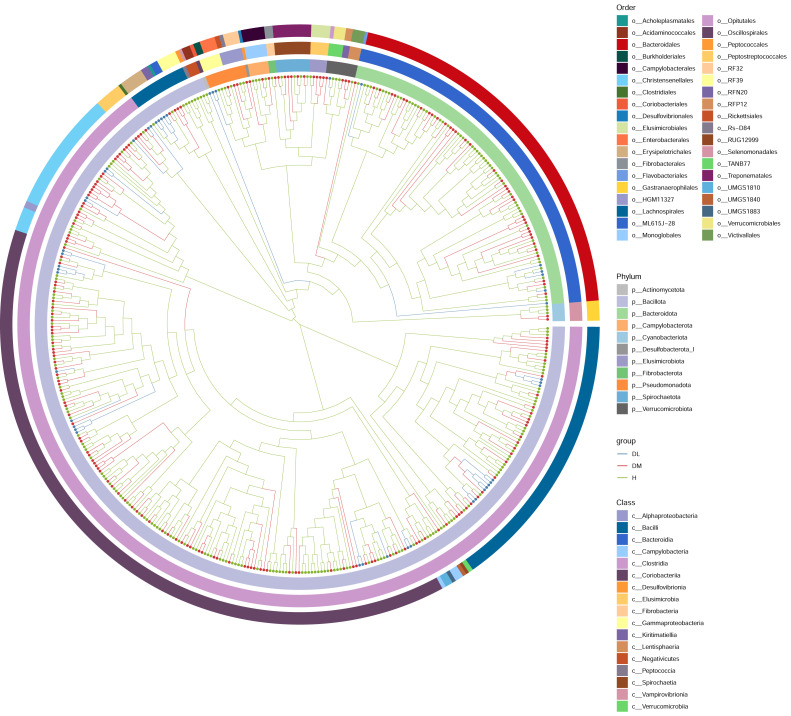
The phylogenetic tree of 482 MAGs from fecal metagenomes of Hu sheep. .

Metagenomic binning of nine fecal metagenomes of Hu sheep lambs was performed. After completing metagenomic binning using Concoct, MaxBin2, MetaBat2, we obtained 8,806 raw bins from fecal metagenomes of Hu sheep (Fig. S1A). After removing redundant bins with ANI > 99% between genomes, we obtained 482 non-redundant MAGs with an estimation of completeness > 80% and contamination < 10% (Fig. 1). Among these MAGs, 324 MAGs exhibited completeness > 90% and contamination < 5% (which were defined as high-quality draft genomes), 186 MAGs exhibited completeness > 95% and contamination < 5%, and 35 MAGs exhibited completeness > 97% and zero contamination (Table S2). Overall, taxonomic labels of all MAGs were identified to family level at least. All 482 MAGs were identified to one kingdom, 11 phyla and 17 classes, 38 orders, 65 families, 479 MAGs to 244 genera and 301 MAGs to 260 species (Table 1, Table S2). The phylogenetic tree of the 482 MAGs was generated based on more than 2,699 most conserved proteins from microbial genomes (Fig. 2).

**Table 1:** The taxonomic classification of MAGs in different levels.

Taxonomic level	Classified MAGs	Identified taxa	Unclassified MAGs
Kingdom	482	1	0
Phylum	482	11	0
Class	482	17	0
Order	482	38	0
Family	482	65	0
Genus	279	244	3
Species	301	260	181

**Figure 3:**
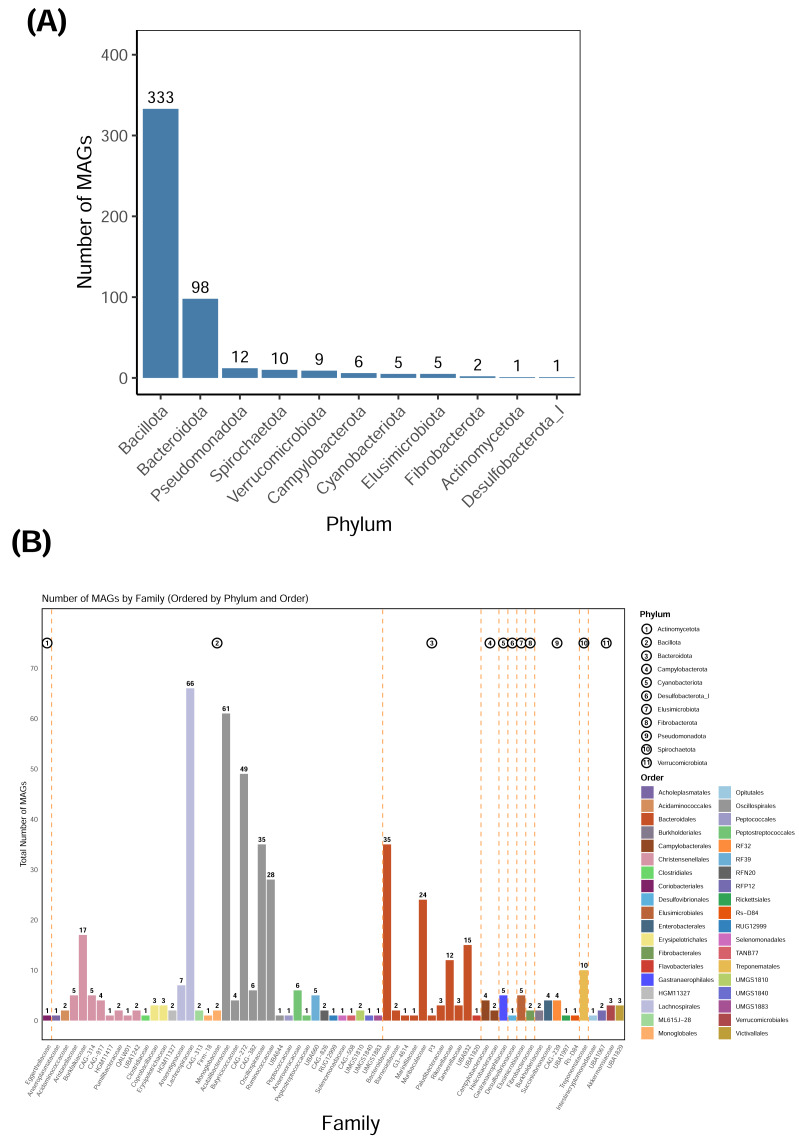
The taxonomic labels of MAGs from fecal metagenomes of Hu sheep. (A) The number of 482 MAGs at different phyla. (B) The number of 482 MAGs at different families.

The taxonomic labels of these MAGs at phylum level were consistent with the phylogenetic tree. All MAGs were classified to 11 bacterial phyla. Most MAGs belonged to Bacillota (NCBI taxonomy: Firmicutes, *n* = 333), followed by Bacteroidota (NCBI taxonomy: Bacteroidetes, *n* = 98), Pseudomonadota (NCBI taxonomy: Proteobacteria, *n* = 12), Spirochaetota (NCBI taxonomy: Spirochaetes, *n* = 10), as well as Verrucomicrobiota (NCBI taxonomy: Verrucomicrobia, *n* = 9) (Fig. 3A). The members of phylum Bacillota predominately belonged to the class Clostridia (*n* = 313). The members of Clostridia mainly included the order Oscillospirales (*n* = 184), Lachnospirales (*n* = 73), and Christensenellales (*n* = 37). The top three families in the class Clostridia are Lachnospiraceae (*n* = 66), Acutalibacteraceae (*n* = 61), and CAG-272 (*n* = 49) (Fig. 3B). The members of phylum Bacteroidota all belonged to the class Bacteroidia (*n* = 98). The members of Bacteroidota included the order Bacteroidales (*n* = 97) and Flavobacteriales (*n* = 1). The top three families in the phylum Bacteroidota are Bacteroidaceae (*n* = 37), Muribaculaceae (*n* = 24), and UBA932 (*n* = 15). All members of phylum Spirochaetota belonged to the class Spirochaetia, including the order Treponematales (*n* = 10) and family Treponemataceae (*n* = 10). Compared with reference genomes of GTDB, 352 MAGs (72.02%) had ANI < 99% (Table S3).

Genome bins were also clustered at a threshold of 95% ANI, resulting in 418 species-level genomes bins (SGBs, Table S3). Therefore, these SGBs represented taxa of the 482 MAGs at species level. At the phylum level, the 418 SGBs were predominantly classified into Bacillota (*n* = 285, 68.2%), Bacteroidota (*n* = 89, 21.3%), and Pseudomonadota (*n* = 11, 2.6%). At the class level, the dominant groups were Clostridia (*n* = 265, 63.4%, within phylum Bacillota), Bacteroidia (*n* = 89, 21.3%, within phylum Bacteroidota), and Bacilli (*n* = 16, 3.8%, within phylum Bacillota). When it comes to order level, the top three orders were Oscillospirales (*n* = 155, 37.1%, the largest clade in Bacillota), Bacteroidales (*n* = 88, 21.1%, a core order of Bacteroidia), and Lachnospirales (*n* = 63, 15.1%, a key order in Bacillota). At the family level, Lachnospiraceae (*n* = 62, 14.8%, within phylum Lachnospirales), Acutalibacteraceae (*n* = 50, 11.9%, within phylum Bacillota), and CAG-272 (*n* = 36, 8.6%) were the most abundant, indicating the dominance of Bacillota and its related taxa in this metagenome.

### Abundances of MAGs in metagenomes

**Figure 4:**
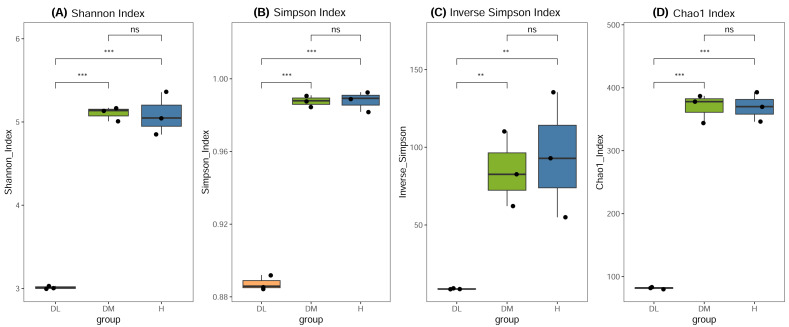
The alpha diversity of MAGs in fecal metagenomes of diarrhea Hu sheep (DM group), recovered hu sheep (DL group) and healthy Hu sheep (H group). (A) Shannon Index. (B) Simpson Index. (C) Inverse Simpson Index. (D) Chao1 Index.

To compare compositional differences of intestinal microbiome between asymptomatic Hu sheep and diarrheic Hu sheep, we conducted quantification and abundance comparisons of the 482 MAGs in fecal metagenomes of Hu sheep. The alpha-diversity of these MAGs including Shannon index, Simpson index, Inverse Simpson Index and Chao1 index were significantly higher in diarrhea and healthy Hu sheep than in Hu sheep recovered from diarrhea (Fig. 4). There were statistically significant differences in the abundance of 163 MAGs in the fecal metagenomes between group DM and group DL (*T*-test, *p* < 0.05). Notably, 89 MAGs in the fecal metagenomes showed statistically significant differences in abundance between group DL and healthy Hu sheep (group H) (*T*-test, *p* < 0.05). However, only 10 MAGs exhibited statistically significant differences in abundance between group DM and group H (*T*-test, *p* < 0.05) (Table S5). Additionally, in the fecal metagenomes of the group DM, the abundance of most members of phylum Bacteroidota (such as family Bacteroidaceae), most members of phylum Spirochaetota (family Treponemataceae), members of phylum Campylobacterota (formerly Epsilonproteobacteria under NCBI taxonomy, family Campylobacteraceae and Helicobacteraceae), and members of family Lachnospiraceae was significantly higher than that in group DL. Several taxa among these MAGs, such as family Akkermansiaceae, Eggerthellaceae and Coprobacillaceae were barely detected in group DM but were commonly present in group H. In contrary, family Gastranaerophilaceae and Oscillospiraceae were hardly detected in the group H but were widespread in group DM (Table S4).

### The functional characterization of 482 MAGs

For further characterization of the functional features, we predicted and annotated the genes of these MAGs. Contigs’ numbers of these 482 MAGs ranged from 11 to 993 (Table S2). The size of the retrieved MAGs ranged from 882,780 bp to 5,698,853 bp, with average contig lengths ranging from 2,848.64 bp to 145,710.54 bp. MAGs harbored between 786 and 5,235 predicted genes, and the average contig length was 967.76 bp. rRNA genes of most MAGs cannot be identified, but 5S rRNA genes of 266 MAGs, 16S rRNA genes of 44 MAGs along with 23S rRNA genes of 24 MAGs were identified from genome sequences. 236 MAGs included full-length 5S rRNA genes, six MAGs included full-length 16S rRNA genes, and 7 MAGs included full-length 23S rRNA genes.

Following the completion of gene prediction, a total of 1,022,739 genes were encompassed within 482 MAGs. We put all genes of 482 MAGs altogether and then constructed non-redundant gene sets at 99%, 95% and 90% identity, yielding 946,817, 896,891, 880,490 clusters, respectively. The non-redundant gene set at 95% identity was further annotated at protein levels. 843,535 (94.00%) genes were mapped to UniProt TrEMBL database with average identity of 75.40%, while 53,356 (5.95%) genes could not be annotated to UniProt TrEMBL database. In addition, 848,097 (94.55%) genes, 845,555 (94.27%) genes, 804,917 (89.75%) genes, 1,443 (0.16%) genes, 34,780 (3.87%) genes, 9,969 (1.11%) genes, 1,421 (0.15%) genes, 3,057 (0.34%) genes and 20,787 (2.31%) genes, could be assigned to UniRef90, UniRef50, eggNOG, CARD, CAZy, VFDB_setB, SARG, BacMet and PHI database respectively.

**Figure 5:**
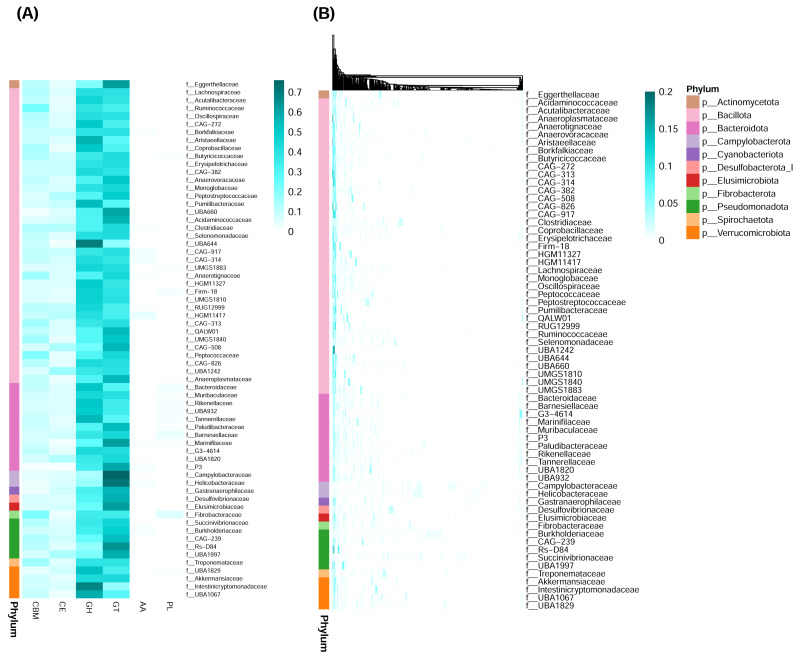
The distribution of CAZymes and virulence genes in MAGs. (A) The distribution of CAZymes genes in MAGs, including glycosyltransferases (GTs), carbohydrate-binding modules (CBMs), carbohydrate esterases (CEs), polysaccharide lyases (PLs), auxiliary activities (AAs). (B) The distribution of virulent genes in MAGs.

We performed prediction of CAZymes in the genome of each MAG. A total of 34,780 CAZymes were identified from all MAGs. Bacillota (*n* = 333), Bacteroidota (*n* = 98) and Pseudomonadota (*n* = 12) carried the most abundant CAZymes. Bacillota, Bacteroidota and Verrucomicrobiota had high percentage of CAZymes in genomes (Fig. S1B). Glycoside hydrolases (GHs) were enriched in the family UBA644, Intestinicryptomonadaceae, UBA1067, Aristaeellaceae and Tannerellaceae (Fig. 5A). Glycosyltransferases (GTs) were prevalent in all MAGs. Carbohydrate-binding modules (CBMs) were mainly identified in the families Ruminococcaceae, Fibrobacteraceae and Peptococcaceae. Carbohydrate esterases (CEs) were mainly found in the phyla Bacillota and Bacteroidota. Polysaccharide lyases (PLs) were mainly identified in the phylum Bacteroidota. Auxiliary activities (AAs) were scarce in most MAGs. The virulence-associated genes in genome of each MAG were predicted based on VFDB database. In total, 9,969 virulence genes were identified in MAGs. Notably, virulence-associated genes were enriched in the Lachnospiraceae, Acutalibacteraceae, Bacteroidaceae, Ruminococcaceae and Oscillospiraceae (Fig. 5B). For Lachnospiraceae and Bacteroidetes, these genes were mainly adaptive genes which support bacterial colonization and nutritional competition, rather than traditional pathogenic genes, which is consistent with their symbiotic role in the gut.

**Figure 6:**
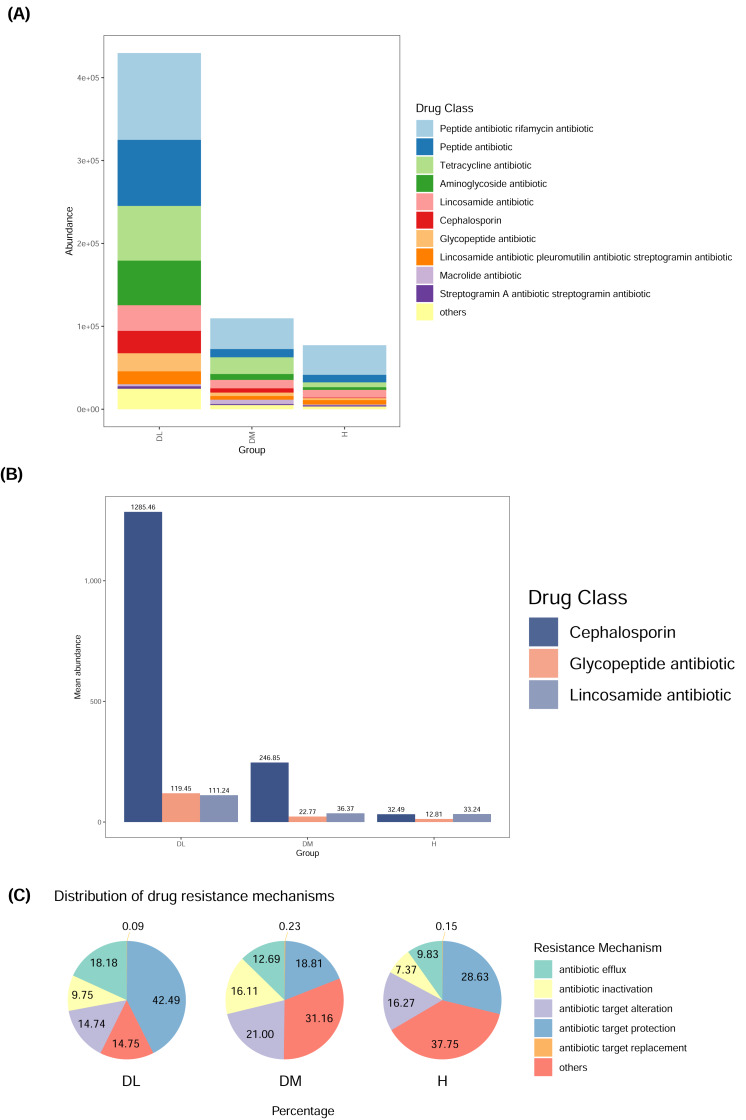
Differences in the number of antibiotic resistance genes (ARGs). (A) The abundance of top 10 drug classes of ARGs in different groups. (B) The abundance of ARGs related to treatment medications. (C) Distribution of drug resistance mechanisms of ARGs.

### Antibiotic resistance genes (ARGs) of 482 MAG

We predicted ARGs in genome of each MAG based on CARD database. A total of 1,443 ARGs were obtained from 482 MAGs with an average identity of 57.37%. The ARGs noted encompassed various antibiotic categories, such as Lincosamide (*n* = 278) (LlmA_23S_CLI was the most abundant ARG family), followed by rifamycin-resistant beta-subunit of RNA polymerase (rpoB) (*n* = 210), pmr phosphoethanolamine transferase (*n* = 136), ATP-binding cassette (ABC) antibiotic efflux pump (*n* = 75) and lsa-type ABC-F protein (*n* = 73) (Table S5).We presented the overall distribution characteristics of resistance genes through the visualization of all categories, while highlighting the intergroup differences in major drug resistance types focused on top 10 drug resistance categories (such as peptide antibiotics and tetracycline antibiotics) (Fig. 6A). The analysis of resistance genes against cephalosporins, lincosamides, and glycopeptide antibiotics showed that these treatment-related resistance genes had higher abundances in the DM and DL, while lower in the group H. For example, the average abundances of cephalosporin resistance genes were 246.85 and 1,285.46 in the DM and DL, respectively, while 21.49 in the group H, indicating the enrichment of treatment-related resistance genes in diarrheic and recovering sheep (Fig. 6B). In the study of drug resistance mechanisms, the main resistance mechanisms in the three groups of sheep included antibiotic-efflux, antibiotic-inactivation, and antibiotic-target-alteration. The proportion of antibiotic-efflux was higher in the DM and DL groups, while the distribution of resistance mechanisms in group H was relatively balanced, suggesting that the diarrheic state may promote the expression of specific resistance mechanisms (Fig. 6C).

### The genomes of the genus *Barnesiella* and *Campylobacter*

Four genus *Barnesiella* MAGs classified at the species level were reconstructed from Hu sheep, belonging to two species: *Barnesiella* sp017410245 and *Barnesiella* sp937891845. Six *Campylobacter* MAGs classified at the species level were also reconstructed, belonging to two species: *Campylobacter vicugnae* and *Campylobacter* sp017506845 (Table S3). At the genus level, the abundances of MAGs belonging to genus *Barnesiella* and *Campylobacter* in the fecal metagenomes of group DM were significantly higher than those in group H (*p* < 0.05) and DL group (*p* < 0.05). Analysis of the four genus *Barnesiella* MAGs identified a total of 567 virulence genes, while analysis of the six *Campylobacter* MAGs identified 382 virulence genes. Among them, the MAGs of *Campylobacter vicugnae* (315), *Barnesiella* sp937891845 (308), and *Barnesiella* sp017410245 (259) carried the most virulence genes. Both *Barnesiella* and *Campylobacter* contained flagella-related genes (such as *flgJ*, *flhF*, *flmH*, and *pdxA*), LPS synthesis and immune regulation-related genes (such as *lpxB*, *lpxC*, and *rfbD*), as well as stress response and metabolic adaptation-related genes (*htpB* (Hsp60), *mpa*, *pyrB*, *regX3*, and *senX3*) (Table S5).

## Discussion

The microbial genomes can provide a better insight to explore the function of intestinal microbiome in metabolism, nutrition and disease of host (Yang et al., 2023). There is a wealth of studies on metagenome-assembled genomes (MAGs) in sheep (Xie et al., 2021; Zhang et al., 2024; Mi et al., 2024; Wang et al., 2025; Su et al., 2026), but research on Chinese Hu sheep remains limited, particularly regarding the intestinal microbiome of Hu sheep under diarrhea and subsequent antibiotic treatment. This study focused on the fecal metagenomes of the preliminary stage of diarrhea (group DM), the late recovery stage of diarrhea (group DL), and the healthy stage (group H). In this study, a total of 482 metagenome-assembled genomes (MAGs) were successfully reconstructed, further expanding the quantity and diversity of intestinal microbial genomes in ruminants. Metagenomic binning is a core technology for acquiring microbial genomic information. Combined binning using multiple tools can leverage the advantages of different algorithms. In this study, three mainstream binning tools within MetaWRAP: Concoct, MaxBin2, and MetaBat2, were used for combined analysis, and strict quality screening ensured the reliability of the MAGs (Uritskiy, Di Ruggiero & Taylor, 2018). Additionally, the application of co-assembly technology effectively improved the recovery rate of low-abundance MAGs while enhancing the assembly potential of novel genomes (Wang et al., 2024b), laying a high-quality genomic foundation for subsequent taxonomic annotation and functional analysis.

Approximately 70% of the MAGs in this study were classified as the phylum Bacillota, followed by Bacteroidota, Pseudomonadota, Spirochaetota, and Verrucomicrobiota. This compositional characteristic is highly consistent with the typical structure of intestinal microbiota in ruminants (Tanca et al., 2017; Cholewińska et al., 2020; Xie et al., 2021). Bacillota and Bacteroidota are the core intestinal microbiota of ruminants: the former supports energy metabolism by producing short-chain fatty acids (SCFAs), while the latter excels in degrading complex carbohydrates, and they maintain the host’s ability to digest fibrous feed (Qing et al., 2019). Spirochaetota (*e.g.*, genus *Treponema*) can participate in cellulose degradation (Salgado et al., 2024), and Verrucomicrobiota (*e.g.*, genus *Akkermansia*) can utilize mucopolysaccharides (Cani et al., 2022), further enriching the functional diversity of intestinal microbiota. At the micro-taxonomic level of dominant phyla, the family Lachnospiraceae is a key group for SCFA production (*e.g.*, genus *Butyrivibrio* can produce butyric acid), and the order Oscillospirales (*e.g.*, genus *Oscillospira*) is closely associated with host dietary fiber metabolism (Vacca et al., 2020). The high proportion of these groups reaffirms the core role of Bacillota in the energy metabolism of sheep. The class Bacteroidia (*e.g.*, genus *Bacteroides*) carries a large number of carbohydrate-active enzymes (CAZymes) and is a core group for degrading hemicellulose and pectin (McKee et al., 2021), and its abundance characteristics are highly adapted to the dietary needs of sheep.

Non-redundant gene sets were constructed and functionally annotated for the genes predicted from the MAGs. The results revealed the core functional reserves and differential characteristics of sheep intestinal microbiota under different health states. Based on database matching, 94.00% of the genes were mapped to the UniProt database (with an average identity of 75.40%), indicating that most functional genes of the MAGs in this study are known conserved functional genes, with clear core metabolic and physiological functions. Among the key functional genes, the phyla Bacillota, Bacteroidota, and Verrucomicrobiota had a higher proportion of CAZyme-encoding genes, which is consistent with the core functional roles of intestinal microbiota in ruminants (Shin et al., 2024; Tan et al., 2025). A large number of virulence genes in this study were enriched in groups such as Lachnospiraceae, Acutalibacteraceae, and Bacteroidaceae. Critically, most members of Lachnospiraceae and Bacteroidaceae are intestinal commensal bacteria (Vacca et al., 2020; Shin et al., 2024); the virulence genes they carry may not be pathogenic genes in the traditional sense, but rather adaptive genes that participate in bacterial adhesion, such as adhering to host intestinal epithelial cells to maintain colonization, and nutrient competition, such as producing antimicrobial substances to inhibit other microorganisms (Sommer & Bäckhed, 2016; Ducarmon et al., 2019). This is crucial for their symbiotic survival in the intestine, rather than for direct pathogenesis. However, when the host is in a diarrheic state, intestinal microecological imbalance may lead to the excessive proliferation of some commensal bacteria, and the expression level of their virulence genes increases, thereby exacerbating intestinal inflammatory responses (Lu & Walker, 2001; Yu et al., 2012). This also suggests that the diarrheic state may activate the virulence genes of specific groups to aggravate intestinal damage (Li et al., 2021), while the shift of virulence gene abundance during the recovery period may be related to the gradual restoration of the intestinal microecology.

In this study, both the DM and DL groups were treated with Shuanghuanglian, cephalosporins, lincomycin, and dexamethasone. The selection pressure of antibiotics is a core factor driving the evolution of intestinal microbial resistance (Baquero, Alvarez-Ortega & Martinez, 2009; Holmes et al., 2016). Therefore, the analysis of antibiotic resistance genes (ARGs) in MAGs can provide key evidence for evaluating the ecological risks of drug intervention and optimizing diarrhea treatment regimens. The ARGs in this study covered multiple antibiotic categories, among which Lincosamide-related ARGs were highly abundant. The high abundance of Lincosamide ARGs is directly related to the use of lincomycin in the DL and DM groups: the continuous use of antibiotics exerts lethal pressure on susceptible bacteria, while resistant bacteria proliferate due to selective advantages, leading to the enrichment of ARGs in the intestine (Yang et al., 2024b).

In this study, the 57.37% average identity between detected ARGs and CARD database entries may reflect the underrepresentation of ARGs specific to the intestinal microbiota of the study’s target species in existing reference databases. We attribute this to several potential factors. First, the CARD database is predominantly curated from clinically derived sequences, which are biased toward human-associated pathogens and may inadequately represent the resistome of commensal bacteria within the ruminant gastrointestinal tract (Gibson, Forsberg & Dantas, 2015; Antelo et al., 2021). Second, the ARGs identified in this study may include divergent homologs of characterized resistance genes that have evolved under distinct selective pressures within the gut environment, such as antibiotic concentrations and intense inter-species microbial competition. These selective pressures may drive sequence divergence while preserving functional resistance activity, as has been previously reported in environmental and animal-associated microbiomes (Rochegüe et al., 2021; Zhang et al., 2026). Third, the use of RGI with default models which include both strict and loose detection models, while maximizing the sensitivity of ARG detection, may have captured remote homologs with inherently lower sequence similarity. Therefore, we emphasize that the ARG annotations presented in this study should be interpreted as computational predictions of putative resistance determinants, and future studies incorporating phenotypic resistance profiling and functional metagenomics would be valuable to validate the resistance activity of these genes. Despite this caveat, the consistent enrichment patterns of treatment-related ARG categories (*e.g.*, cephalosporin and lincosamide resistance genes) across diarrheic and recovery groups provide biologically meaningful support for the association between antibiotic treatment and ARG selection.

When further investigating ARGs directly related to the drugs used, the abundances of cephalosporin, lincosamide, and glycopeptide ARGs in the DM and DL groups were significantly higher than those in the H group, and the average abundance of cephalosporin ARGs in the DL group was much higher than that in the DM and H groups. This result reveals that antibiotic treatment indeed leads to the directional enrichment of resistance genes (Rochegüe et al., 2021). Cephalosporins and lincosamides were the core therapeutic drugs in this study, and their use directly selects for microorganisms carrying the corresponding resistance genes. In addition, the higher abundance of ARGs detected in the DL group may be related to duration of drug exposure: the DM group is in the acute phase of diarrhea, where drugs have just begun to act, and resistant bacteria start to proliferate but have not yet become dominant. In contrast, the DL group is in the recovery phase, where drugs have been acting for a period of time, allowing resistant bacteria to fully proliferate in the intestine, leading to a further increase in ARG abundance. This indicates that even if diarrhea symptoms are relieved, resistance genes in the intestine may still remain at a high level, posing a risk of resistance gene residue (Van Boeckel et al., 2015; Yang et al., 2022; Herawati et al., 2023).

In terms of resistance mechanisms, the main resistance mechanisms of intestinal microbiota in the three groups included antibiotic efflux, antibiotic inactivation, and antibiotic target alteration. However, the proportion of the antibiotic efflux mechanism in the DM and DL groups was significantly higher, while the distribution of the three mechanisms in the H group was relatively balanced. The antibiotic efflux enables microorganisms to expel intracellular antibiotics through efflux pumps (Holmes et al., 2016), reducing intracellular drug concentration to achieve resistance (Levy, 2002). Therefore, under continuous drug selection pressure, microorganisms carrying efflux pump genes are more likely to become dominant groups, leading to an increase in the proportion of the efflux mechanism (Nikaido & Zgurskaya, 1999; Holmes et al., 2016). These result indicates that antibiotic treatment during diarrhea in Hu sheep not only increases the number of ARGs but also changes the composition of resistance mechanisms, driving intestinal microbiota to a resistance strategy of active efflux. This shift may further enhance the resistance of microorganisms to subsequent drug treatments and increase the difficulty of treating diarrhea in the future.

In addition, although Shuanghuanglian (a traditional Chinese medicine compound) is not an antibiotic in the traditional sense, existing studies have shown that some components of traditional Chinese medicine may exert selection pressure on microorganisms (Zhu et al., 2025). The DM and DL groups were jointly exposed to the combined pressure of traditional Chinese medicine and Western antibiotics, and the evolution of their resistance may be more complex than that under single-drug intervention. Whether the enrichment of ARGs in this study is related to Shuanghuanglian requires further analysis in combination with specific traditional Chinese medicine components (Zhou et al., 2011).

In the study of intestinal microbial diversity, the overall difference in MAGs between diarrheic sheep (DM group) and healthy sheep (H group) was not significant, but both groups showed significant differences from diarrhea-recovered sheep (DL group), and the diversity of the DL group was reduced. This reflects the dynamic evolution of intestinal microecology under the combined action of disease pressure and antibiotic intervention. The intestinal microecology of healthy sheep (H group) is in a steady state, and core microbiota such as Bacillota and Bacteroidota maintain key functions such as carbohydrate degradation and energy metabolism through synergy (Cholewińska et al., 2020; Xie et al., 2021). Although diarrheic sheep (DM group) are in a disease state, the disorder of their intestinal microecology is reflected in fluctuations in microbiota abundance rather than disappearance of core groups (Martel et al., 2022). The occurrence of diarrhea may be caused by increased abundance of specific pathogenic bacteria rather than large-scale replacement of core functional microbiota, resulting in no significant difference in the overall taxonomic composition of MAGs. The significant difference between the DL group and the DM/H groups is mainly driven by the selective of antibiotics, which reshapes the composition of MAGs (Theodosiou et al., 2023). This is presumably due to the massive elimination of susceptible bacteria and the enrichment and proliferation of resistant bacteria (Zarrinpar et al., 2018). Cephalosporins kill susceptible bacteria by inhibiting bacterial cell wall synthesis, while lincomycin exerts its effect by inhibiting bacterial protein synthesis (Hornish & Kotarski, 2002). The combined use of these two drugs exerts strong selection pressure on sensitive core microbiota in the intestine, leading to a sharp decline in the abundance of these strains or even their disappearance from the intestine (Holmes et al., 2016; Zarrinpar et al., 2018). This indicates that although the DL group had recovered from diarrhea symptoms, its intestinal microecology has not yet returned to a healthy state (Maciel-Fiuza et al., 2023).

The genus *Campylobacter* is a common pathogen causing intestinal diseases in humans and animals, such as *Campylobacter jejuni*, which can cause human enteritis (Liu et al., 2018; Wieczorek, Wołkowicz & Osek, 2018). In this study, the abundances of *Barnesiella* and *Campylobacter* MAGs in the diarrheic DM group were significantly higher than those in the H and DL groups (*p* < 0.05). Additionally, four *Barnesiella* MAGs carried 567 virulence genes in total, and six *Campylobacter* MAGs carried 382 virulence genes in total. This result indicates that intestinal microecological imbalance (such as a reduction of beneficial bacteria, impairment of intestinal barrier (Fu et al., 2022) in the diarrheic state provides ecological niches for these two bacterial genera, enabling them to proliferate by competing for nutrients and inhibiting other microorganisms (Maciel-Fiuza et al., 2023). On the other hand, the decreased abundance of these two genera in the DL group suggests that as diarrhea symptoms are relieved and intestinal microecology is restored, their competitive advantages weaken, further confirming the correlation between their abundance changes and the diarrheic state (Fu et al., 2022). Notably, although the DL group is in the recovery phase, the abundance of these two genera is still lower than that in the H group, which may be related to the residual effects of antibiotic treatment: antibiotics control diarrhea symptoms but also alter the intestinal microbiota structure, resulting in incomplete recovery of beneficial bacteria and failure to completely inhibit the growth of these two bacterial genera. Notably, dietary intervention represents a promising alternative to reduce reliance on antibiotics for diarrhea management in livestock (Bagaria et al., 2024). For instance, recent studies on liquid whey-supplemented diets in pigs have demonstrated that such diets can enrich beneficial gut taxa (*e.g.*, *Lactobacillus*, *Bifidobacterium*), enhance intestinal barrier function by upregulating tight junction proteins, and reduce inflammatory responses (Sutera et al., 2023; Mhalhel et al., 2025). These findings offer valuable comparative context for Hu sheep diarrhea intervention. Future studies could explore whether similar fermentable dietary supplements (*e.g.*, whey-derived oligosaccharides) could enhance SCFA production in Hu sheep intestines, thereby restoring microecological balance and alleviating diarrhea.

Although *Barnesiella* is generally considered an intestinal commensal bacterium (Zhang et al., 2025), similar to Lachnospiraceae and Bacteroidetes, the virulence genes it carries may mainly be adaptive genes that support intestinal colonization. Only in a state of diarrhea, when the gut microbiota is imbalanced, the high expression of its virulence genes in the diarrheic state may transform it from a commensal bacterium to an opportunistic pathogen (Freter, 1992; Mancabelli et al., 2017), which participates in the diarrhea process by exacerbating inflammatory responses. This finding suggests that the pathogenicity or beneficiality of intestinal microbiota is not fixed but dynamically regulated by the host’s health status and intestinal environment (Fu et al., 2022).

In this study, high-quality intestinal MAGs of Hu sheep were recovered through metagenomic binning. The taxonomic composition of these MAGs was clarified with Bacillota and Bacteroidota as the core, and the synergistic adaptation between ruminant intestinal microbiota and host diet was verified. Meanwhile, this study revealed that disease pressure and antibiotic intervention reshaped intestinal MAGs. In addition, by focusing on *Barnesiella* and *Campylobacter*, this study analyzed their abundance advantages and virulence mechanisms in diarrhea, providing a new perspective on the microecological driving mechanism of sheep diarrhea.

## Conclusions

This study successfully reconstructed 482 non-redundant MAGs with high quality fecal metagenomes of Hu sheep lambs using combined binning tools and co-assembly. These MAGs were distributed across 11 phyla dominated by Bacillota and Bacteroidota, enriching the genomic resources of ruminant intestinal microbiota. Functional annotation showed the 482 MAGs contained abundant carbohydrate-active enzymes (CAZymes, 34,780), virulence genes (9,969), and antibiotic resistance genes (ARGs, 1,443). Diarrheic (DM) and diarrhea-recovered (DL) groups had higher abundances of treatment-related ARGs (cephalosporins, lincosamides) and antibiotic-efflux resistance mechanisms than healthy (H) group, with DL group showing resistance gene residue risk. Alpha diversity of MAGs was lower in DL group than DM and H groups. *Barnesiella* and *Campylobacter* MAGs had significantly higher abundances and more virulence genes in DM group, suggesting their association with diarrhea. Overall, this study clarifies the taxonomic and functional characteristics of Hu sheep intestinal MAGs under different health states, providing new insights into the microecological mechanisms of sheep diarrhea and a basis for optimizing antibiotic treatment.

## Supplemental Information

10.7717/peerj.21574/supp-1 Supplemental Information 1 The annotation characterization in MAGs (A) The distributions of contigs’ number (upper left), contigs’ total lengths (upper right), contigs’ average lengths (low left) and MAGs genes’ number (low right). (B) The percentage of CAZymes in MAGs at different phylum.

10.7717/peerj.21574/supp-2 Supplemental Information 2 The summary of information of samples

10.7717/peerj.21574/supp-3 Supplemental Information 3 The characterization of completeness, contamination and genomes of MAGs

10.7717/peerj.21574/supp-4 Supplemental Information 4 The taxonomy labels of MAGs annotated by GTDB-tk

10.7717/peerj.21574/supp-5 Supplemental Information 5 The MAGs with significant difference in hu sheep fecal metagenomes

10.7717/peerj.21574/supp-6 Supplemental Information 6 The main ARG family in MAGs

## Additional Information and Declarations

### Data Availability

The following information was supplied regarding data availability: The raw sequence data are available at NCBI BioProject: PRJNA1334248, SRR35663771–SRR35663779.
