# Supplementary figures and images for: Effects of diarrhea and antibiotic-induced microbial elimination on dynamic changes in fecal microbial communities and antibiotic resistance of Hu sheep lambs (Ovis aries)
Source: PeerJ. 2026 Jul 31;14:e21574. doi: 10.7717/peerj.21574 (PMC13431306; doi:10.7717/peerj.21574)

**(a)**

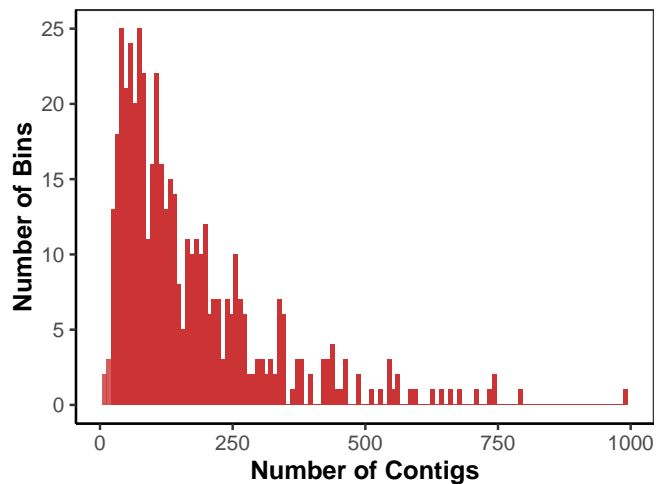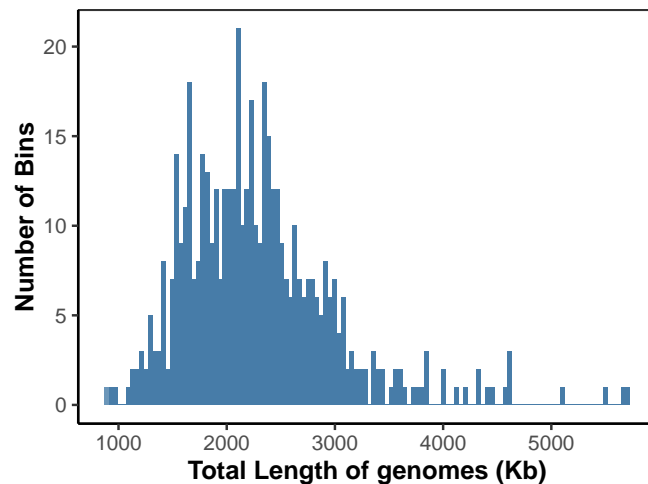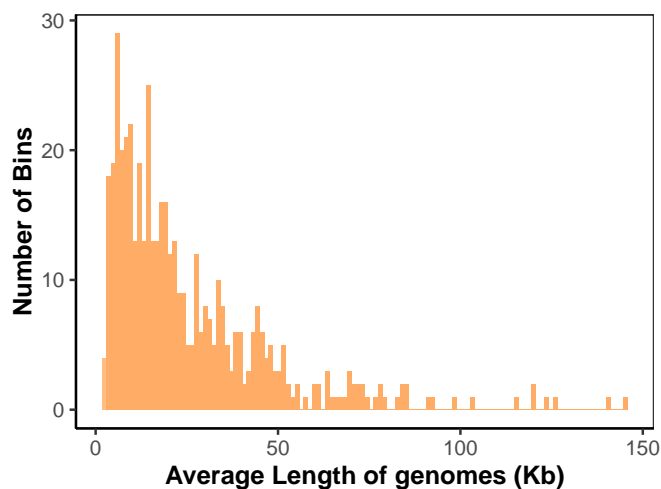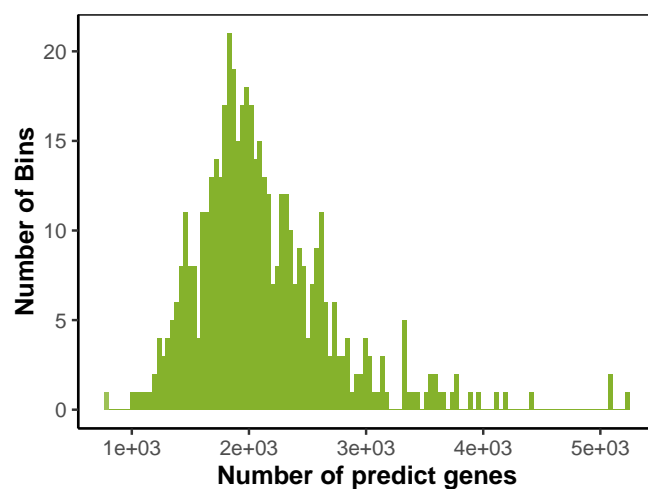

**(b)**

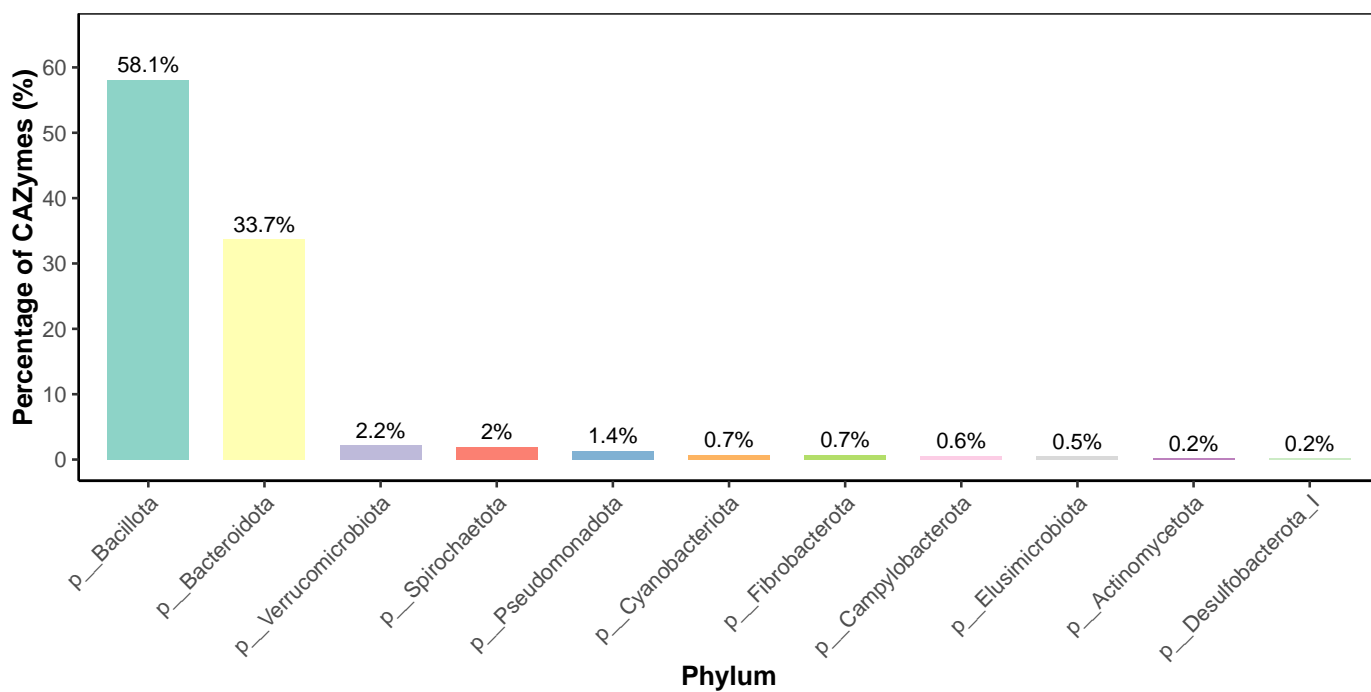

Supplement: Supplemental Information 1 — (A) The distributions of contigs’ number (upper left), contigs’ total lengths (upper right), contigs’ average lengths (low left) and MAGs genes’ number (low right). (B) The percentage of CAZymes in MAGs at different phylum. [file peerj-14-21574-s001.pdf]
