# Supplementary material for: Effects of diarrhea and antibiotic-induced microbial elimination on dynamic changes in fecal microbial communities and antibiotic resistance of Hu sheep lambs (Ovis aries)
Source: PeerJ. 2026 Jul 31;14:e21574. doi: 10.7717/peerj.21574 (PMC13431306; doi:10.7717/peerj.21574)
Supplement: Supplemental Information 2 [file peerj-14-21574-s002.docx]

| Supplementary Table S1. The summary of information of samples | | | | | | | | | | |
| --- | --- | --- | --- | --- | --- | --- | --- | --- | --- | --- |
| Sample ID | group | Group short name | Site | Latitude | Longitude | Human intervention | Sample Type | Antibiotic use | Age | Gender |
| H1 | Healthy | H | Yuexi County, Liangshan, Sichuan Province, China | 28.62 | 102.49 | Captivity | Faeces | None | ~2 months | Female |
| H2 | Healthy | H | Yuexi County, Liangshan, Sichuan Province, China | 28.62 | 102.49 | Captivity | Faeces | None | ~2 months | Female |
| H3 | Healthy | H | Yuexi County, Liangshan, Sichuan Province, China | 28.62 | 102.49 | Captivity | Faeces | None | ~2 months | Female |
| DM1 | Preliminary stage of diarrhea | DM | Yuexi County, Liangshan, Sichuan Province, China | 28.62 | 102.49 | Captivity | Faeces | Shuanghuanglian, Cefazolin, Lincomycin, and Dexamethasone (0.2 mL dosage) | ~2 months | Female |
| DM2 | Preliminary stage of diarrhea | DM | Yuexi County, Liangshan, Sichuan Province, China | 28.62 | 102.49 | Captivity | Faeces | Shuanghuanglian, Cefazolin, Lincomycin, and Dexamethasone (0.2 mL dosage) | ~2 months | Female |
| DM3 | Preliminary stage of diarrhea | DM | Yuexi County, Liangshan, Sichuan Province, China | 28.62 | 102.49 | Captivity | Faeces | Shuanghuanglian, Cefazolin, Lincomycin, and Dexamethasone (0.2 mL dosage) | ~2 months | Female |
| DL1 | Late recovery stage of diarrhea | DL | Yuexi County, Liangshan, Sichuan Province, China | 28.62 | 102.49 | Captivity | Faeces | Shuanghuanglian, Cefazolin, Lincomycin, and Dexamethasone (0.2 mL dosage) | ~2 months | Female |
| DL2 | Late recovery stage of diarrhea | DL | Yuexi County, Liangshan, Sichuan Province, China | 28.62 | 102.49 | Captivity | Faeces | Shuanghuanglian, Cefazolin, Lincomycin, and Dexamethasone (0.2 mL dosage) | ~2 months | Female |
| DL3 | Late recovery stage of diarrhea | DL | Yuexi County, Liangshan, Sichuan Province, China | 28.62 | 102.49 | Captivity | Faeces | Shuanghuanglian, Cefazolin, Lincomycin, and Dexamethasone (0.2 mL dosage) | ~2 months | Female |
