# Supplementary material for: Effects of diarrhea and antibiotic-induced microbial elimination on dynamic changes in fecal microbial communities and antibiotic resistance of Hu sheep lambs (Ovis aries)
Source: PeerJ. 2026 Jul 31;14:e21574. doi: 10.7717/peerj.21574 (PMC13431306; doi:10.7717/peerj.21574)
Supplement: Supplemental Information 3 [file peerj-14-21574-s003.docx]

| Supplementary Table S2. The characterization of completeness, contamination and genomes of MAGs. | | | | | | |
| --- | --- | --- | --- | --- | --- | --- |
| MAGs | completeness | contamination | N50 | num_seqs | sum_len | avg_len |
| DL1.bin.17 | 99.25 | 0 | 220468 | 4184 | 4624626 | 1105.31 |
| DL1.bin.23 | 95.32 | 0.61 | 63757 | 2584 | 2584230 | 1000.09 |
| DL1.bin.28 | 98.11 | 0 | 41728 | 2110 | 1923081 | 911.41 |
| DL1.bin.35 | 99.37 | 0 | 32475 | 4129 | 3993063 | 967.08 |
| DL2.bin.13 | 93.95 | 0.83 | 204829 | 2181 | 2354268 | 1079.44 |
| DL2.bin.18 | 97.96 | 0.57 | 15319 | 2830 | 2551722 | 901.67 |
| DL2.bin.21 | 98.32 | 1.03 | 10445 | 2579 | 2167002 | 840.25 |
| DL2.bin.22 | 91.7 | 0.7 | 338562 | 1356 | 1392828 | 1027.16 |
| DL2.bin.26 | 98.66 | 0.67 | 75300 | 2086 | 1903683 | 912.6 |
| DL2.bin.28 | 88.15 | 1.29 | 7512 | 2530 | 2215623 | 875.74 |
| DL2.bin.29 | 80.75 | 2.15 | 12651 | 2011 | 1828419 | 909.21 |
| DL2.bin.3 | 100 | 0.7 | 149490 | 4407 | 3796482 | 861.47 |
| DL2.bin.30 | 97.96 | 0 | 80370 | 2461 | 2530053 | 1028.06 |
| DL2.bin.33 | 99.26 | 0.37 | 125032 | 3650 | 4067274 | 1114.32 |
| DL2.bin.36 | 97.43 | 0.32 | 25857 | 5079 | 5093766 | 1002.91 |
| DL2.bin.38 | 100 | 0 | 86768 | 2275 | 2414328 | 1061.24 |
| DL2.bin.6 | 98.92 | 0.81 | 48386 | 3018 | 2795553 | 926.29 |
| DL3.bin.10 | 93.43 | 0.22 | 11821 | 2721 | 2352102 | 864.43 |
| DL3.bin.24 | 85.29 | 1.53 | 20189 | 3331 | 3722709 | 1117.6 |
| DL3.bin.25 | 97.32 | 0.67 | 22036 | 1882 | 1705215 | 906.07 |
| DL3.bin.35 | 100 | 0 | 94355 | 3551 | 3175977 | 894.39 |
| DL3.bin.38 | 100 | 0 | 38517 | 2474 | 2334813 | 943.74 |
| DL3.bin.40 | 95.65 | 1.63 | 23033 | 5235 | 4955706 | 946.65 |
| DL3.bin.6 | 89.72 | 0.05 | 12335 | 1699 | 1620258 | 953.65 |
| DL3.bin.7 | 99.37 | 0.63 | 68969 | 3315 | 3031416 | 914.45 |
| DM1.bin.10 | 93.74 | 0.89 | 35306 | 2182 | 2139015 | 980.3 |
| DM1.bin.102 | 99.62 | 0 | 97682 | 2726 | 2961582 | 1086.42 |
| DM1.bin.104 | 86.71 | 0 | 30781 | 1788 | 1694295 | 947.59 |
| DM1.bin.105 | 94.11 | 1.29 | 12919 | 2293 | 2266065 | 988.25 |
| DM1.bin.107 | 96.37 | 0.81 | 92435 | 2593 | 2590515 | 999.04 |
| DM1.bin.112 | 95.4 | 1.4 | 33906 | 2089 | 2190204 | 1048.45 |
| DM1.bin.118 | 98.3 | 0 | 83318 | 2042 | 2088567 | 1022.8 |
| DM1.bin.12 | 88.17 | 0.26 | 36582 | 2030 | 2242116 | 1104.49 |
| DM1.bin.127 | 95.95 | 0.34 | 45391 | 1992 | 1982745 | 995.35 |
| DM1.bin.13 | 98.86 | 0.6 | 86645 | 2569 | 2762508 | 1075.32 |
| DM1.bin.131 | 94.83 | 2.52 | 23850 | 2618 | 2642760 | 1009.46 |
| DM1.bin.133 | 84.68 | 1.34 | 4578 | 1993 | 1512882 | 759.1 |
| DM1.bin.134 | 89.71 | 3.85 | 19559 | 1898 | 1759458 | 927.01 |
| DM1.bin.139 | 95.08 | 0 | 79669 | 1684 | 1755357 | 1042.37 |
| DM1.bin.141 | 97.55 | 0.38 | 76932 | 2096 | 2231202 | 1064.5 |
| DM1.bin.22 | 92.14 | 4.44 | 6883 | 1417 | 1155537 | 815.48 |
| DM1.bin.38 | 99.63 | 0.12 | 221906 | 2620 | 2851941 | 1088.53 |
| DM1.bin.39 | 81.16 | 2.29 | 3642 | 3429 | 2794242 | 814.89 |
| DM1.bin.44 | 97.99 | 0 | 67514 | 2017 | 2019762 | 1001.37 |
| DM1.bin.54 | 95.17 | 3.3 | 47914 | 2097 | 2091885 | 997.56 |
| DM1.bin.60 | 93.27 | 0.19 | 145655 | 2307 | 2544159 | 1102.8 |
| DM1.bin.62 | 87.14 | 1.59 | 22054 | 1752 | 1593021 | 909.26 |
| DM1.bin.68 | 97.99 | 1.57 | 23955 | 1875 | 1787292 | 953.22 |
| DM1.bin.71 | 100 | 0 | 114120 | 2372 | 2309925 | 973.83 |
| DM1.bin.73 | 95.03 | 0.81 | 22787 | 1417 | 1406664 | 992.71 |
| DM1.bin.75 | 100 | 0 | 115952 | 3099 | 3380895 | 1090.96 |
| DM1.bin.77 | 88.05 | 6.59 | 10454 | 1940 | 1604172 | 826.89 |
| DM1.bin.78 | 86.26 | 1.68 | 6922 | 1735 | 1599825 | 922.09 |
| DM1.bin.83 | 95.66 | 0.69 | 29782 | 1816 | 1866945 | 1028.05 |
| DM1.bin.85 | 92.47 | 0.21 | 64308 | 1924 | 2106186 | 1094.69 |
| DM1.bin.98 | 91.43 | 3.45 | 17985 | 3369 | 2927274 | 868.89 |
| DM2.bin.100 | 95.9 | 2.69 | 37482 | 2219 | 2118330 | 954.63 |
| DM2.bin.104 | 91.03 | 1.3 | 34207 | 2130 | 2344197 | 1100.56 |
| DM2.bin.105 | 87.94 | 0.95 | 13404 | 1436 | 1489566 | 1037.3 |
| DM2.bin.113 | 88.68 | 2.07 | 7936 | 2238 | 2010726 | 898.45 |
| DM2.bin.121 | 98.43 | 0.67 | 57171 | 2233 | 2117775 | 948.4 |
| DM2.bin.18 | 80.03 | 2.75 | 3893 | 2018 | 1593276 | 789.53 |
| DM2.bin.23 | 96.11 | 0.37 | 51922 | 2696 | 2951562 | 1094.79 |
| DM2.bin.28 | 95.08 | 0 | 194904 | 1612 | 1507131 | 934.94 |
| DM2.bin.36 | 82.36 | 4.53 | 5217 | 1949 | 1709487 | 877.11 |
| DM2.bin.50 | 80.28 | 2.35 | 3510 | 1965 | 1442826 | 734.26 |
| DM2.bin.52 | 96.63 | 0 | 36602 | 1865 | 1856136 | 995.25 |
| DM2.bin.54 | 95.2 | 0.22 | 60821 | 2083 | 2140359 | 1027.54 |
| DM2.bin.61 | 81.4 | 4.42 | 6304 | 2430 | 2351286 | 967.61 |
| DM2.bin.66 | 96.51 | 1.34 | 26589 | 2156 | 2136639 | 991.02 |
| DM2.bin.72 | 99.88 | 0 | 162233 | 1547 | 1428456 | 923.37 |
| DM2.bin.81 | 98.28 | 1.55 | 56374 | 2616 | 2469522 | 944.01 |
| DM2.bin.83 | 90.13 | 1.95 | 15393 | 2188 | 2258574 | 1032.26 |
| DM2.bin.84 | 98.08 | 0.48 | 174904 | 1771 | 1862226 | 1051.51 |
| DM2.bin.94 | 91.71 | 2.24 | 5692 | 1736 | 1447266 | 833.68 |
| DM2.bin.95 | 82.38 | 2.06 | 5066 | 1281 | 1022532 | 798.23 |
| DM3.bin.1 | 85.84 | 0.34 | 3462 | 2064 | 1737000 | 841.57 |
| DM3.bin.100 | 86.83 | 3.14 | 5953 | 1723 | 1470000 | 853.16 |
| DM3.bin.101 | 89.46 | 3.64 | 6995 | 3769 | 3462951 | 918.8 |
| DM3.bin.102 | 93.96 | 0 | 52063 | 2133 | 2110113 | 989.27 |
| DM3.bin.104 | 80.26 | 2.03 | 6114 | 2119 | 1812492 | 855.35 |
| DM3.bin.106 | 84.56 | 2.68 | 7333 | 1871 | 1833852 | 980.15 |
| DM3.bin.110 | 86.26 | 2.14 | 33264 | 2724 | 3184431 | 1169.03 |
| DM3.bin.111 | 93.6 | 0.89 | 17313 | 1748 | 1731648 | 990.65 |
| DM3.bin.113 | 99.51 | 0 | 104689 | 1630 | 1484058 | 910.47 |
| DM3.bin.118 | 87.94 | 3.83 | 11235 | 1917 | 1623852 | 847.08 |
| DM3.bin.12 | 88.03 | 3.75 | 11129 | 2164 | 1795698 | 829.8 |
| DM3.bin.122 | 96.53 | 1.49 | 88398 | 2449 | 2693757 | 1099.94 |
| DM3.bin.124 | 93.82 | 1.12 | 5212 | 1793 | 1418700 | 791.24 |
| DM3.bin.128 | 96 | 2 | 40360 | 1526 | 1514616 | 992.54 |
| DM3.bin.129 | 94.76 | 4.86 | 8626 | 1585 | 1453677 | 917.15 |
| DM3.bin.20 | 96.43 | 0 | 37490 | 2347 | 2134665 | 909.53 |
| DM3.bin.21 | 93.96 | 0.22 | 61937 | 2094 | 2065287 | 986.29 |
| DM3.bin.23 | 93.55 | 0 | 28712 | 1473 | 1316424 | 893.7 |
| DM3.bin.26 | 95.28 | 0.09 | 38374 | 1828 | 1996587 | 1092.22 |
| DM3.bin.31 | 96.55 | 8.32 | 65800 | 1779 | 1605582 | 902.52 |
| DM3.bin.33 | 82.35 | 3.49 | 32278 | 1417 | 1333527 | 941.09 |
| DM3.bin.35 | 91.53 | 0 | 59162 | 1391 | 1322919 | 951.06 |
| DM3.bin.36 | 83.91 | 3.83 | 3773 | 2444 | 1912710 | 782.61 |
| DM3.bin.37 | 97.97 | 2.13 | 62301 | 2510 | 2437167 | 970.98 |
| DM3.bin.38 | 88.3 | 0.15 | 58252 | 2037 | 2093940 | 1027.95 |
| DM3.bin.43 | 86.32 | 1.04 | 6572 | 2344 | 2219028 | 946.68 |
| DM3.bin.44 | 80.59 | 8.05 | 4267 | 2906 | 2626335 | 903.76 |
| DM3.bin.46 | 96.43 | 0.5 | 82339 | 2036 | 2365560 | 1161.87 |
| DM3.bin.49 | 86.13 | 0 | 74460 | 1436 | 1507218 | 1049.59 |
| DM3.bin.5 | 93.56 | 1.85 | 16385 | 1824 | 1749675 | 959.25 |
| DM3.bin.53 | 97.72 | 1.01 | 22490 | 2167 | 2127534 | 981.79 |
| DM3.bin.55 | 81.66 | 0.69 | 13142 | 1225 | 1101831 | 899.45 |
| DM3.bin.56 | 89.09 | 1.8 | 58949 | 2234 | 2445141 | 1094.51 |
| DM3.bin.57 | 95.75 | 0 | 35901 | 2095 | 2099331 | 1002.07 |
| DM3.bin.58 | 91.98 | 1.24 | 42866 | 2883 | 3020700 | 1047.76 |
| DM3.bin.60 | 90.75 | 3.13 | 12566 | 1935 | 1728858 | 893.47 |
| DM3.bin.61 | 92.34 | 0.08 | 57478 | 1500 | 1447311 | 964.87 |
| DM3.bin.63 | 89.15 | 4.03 | 12140 | 2013 | 1806150 | 897.24 |
| DM3.bin.67 | 96.45 | 1.06 | 28448 | 1768 | 1701558 | 962.42 |
| DM3.bin.68 | 95.48 | 0.56 | 51029 | 3035 | 3231723 | 1064.82 |
| DM3.bin.69 | 100 | 1.01 | 28603 | 3384 | 3331377 | 984.45 |
| DM3.bin.7 | 99.3 | 0.93 | 60357 | 3189 | 3480384 | 1091.37 |
| DM3.bin.70 | 98.89 | 0.12 | 209437 | 2263 | 2488074 | 1099.46 |
| DM3.bin.75 | 91.57 | 0.11 | 17676 | 1123 | 1137684 | 1013.08 |
| DM3.bin.76 | 90.59 | 0.7 | 14960 | 1359 | 1276809 | 939.52 |
| DM3.bin.78 | 90.18 | 1.16 | 9574 | 2073 | 1984914 | 957.51 |
| DM3.bin.8 | 98.52 | 1.4 | 107522 | 2346 | 2491707 | 1062.11 |
| DM3.bin.85 | 94.16 | 3.29 | 11970 | 1946 | 1652304 | 849.08 |
| DM3.bin.87 | 89.26 | 1.36 | 9177 | 1772 | 1600179 | 903.04 |
| DM3.bin.88 | 85.16 | 0.16 | 7700 | 1004 | 841302 | 837.95 |
| DM3.bin.89 | 97.99 | 1.05 | 20976 | 1843 | 1684302 | 913.89 |
| DM3.bin.90 | 84.25 | 0.34 | 5531 | 1542 | 1459173 | 946.29 |
| DM3.bin.91 | 95.31 | 0.93 | 35501 | 2542 | 2716533 | 1068.66 |
| grpDL.bin.1 | 98.64 | 0 | 103132 | 2682 | 2699556 | 1006.55 |
| grpDL.bin.12 | 84.12 | 2.68 | 25201 | 2404 | 2134179 | 887.76 |
| grpDL.bin.13 | 90.94 | 1.75 | 123021 | 2860 | 2700771 | 944.33 |
| grpDL.bin.16 | 99.06 | 0 | 30784 | 1684 | 1510302 | 896.85 |
| grpDL.bin.21 | 86.16 | 0.94 | 4467 | 2430 | 2104452 | 866.03 |
| grpDL.bin.24 | 96.32 | 9.6 | 151002 | 3732 | 4203132 | 1126.24 |
| grpDL.bin.27 | 80.18 | 0 | 26915 | 3516 | 3050757 | 867.68 |
| grpDL.bin.28 | 99.33 | 0.67 | 82188 | 2641 | 2484753 | 940.84 |
| grpDL.bin.3 | 92.62 | 0.17 | 35382 | 2749 | 2436378 | 886.28 |
| grpDL.bin.32 | 99.46 | 0 | 73739 | 3896 | 4186488 | 1074.56 |
| grpDL.bin.37 | 93.51 | 0.67 | 28734 | 1989 | 1814382 | 912.21 |
| grpDL.bin.4 | 96.92 | 0 | 80457 | 5085 | 5073033 | 997.65 |
| grpDL.bin.44 | 87.78 | 0 | 7140 | 1629 | 1353237 | 830.72 |
| grpDL.bin.47 | 97.65 | 0 | 43118 | 2625 | 2401002 | 914.67 |
| grpDL.bin.48 | 99.42 | 0.29 | 122985 | 2322 | 2239557 | 964.49 |
| grpDL.bin.49 | 87.47 | 0.15 | 6206 | 2311 | 1806117 | 781.53 |
| grpDL.bin.51 | 93.96 | 1.07 | 20384 | 2506 | 2527869 | 1008.73 |
| grpDL.bin.6 | 97.08 | 1.34 | 24829 | 3319 | 3027426 | 912.15 |
| grpDL.bin.62 | 97.99 | 0 | 346290 | 2377 | 2078967 | 874.62 |
| grpDL.bin.63 | 95.23 | 3.23 | 12337 | 2856 | 2592486 | 907.73 |
| grpDL.bin.66 | 83.96 | 1.04 | 10866 | 1803 | 1656258 | 918.61 |
| grpDL.bin.7 | 93.51 | 3.47 | 8466 | 2902 | 2384385 | 821.64 |
| grpDM.bin.106 | 96.15 | 1.28 | 34827 | 2600 | 2676174 | 1029.3 |
| grpDM.bin.112 | 95 | 0 | 61147 | 1418 | 1528299 | 1077.78 |
| grpDM.bin.115 | 95.53 | 0 | 74278 | 1580 | 1583592 | 1002.27 |
| grpDM.bin.116 | 86.27 | 0.34 | 32370 | 1508 | 1450890 | 962.13 |
| grpDM.bin.119 | 91.83 | 4.05 | 7463 | 1636 | 1437441 | 878.63 |
| grpDM.bin.123 | 82.21 | 1.41 | 26344 | 2635 | 2560056 | 971.56 |
| grpDM.bin.126 | 92.17 | 1.6 | 28560 | 3147 | 3329424 | 1057.97 |
| grpDM.bin.13 | 95.72 | 0.67 | 50708 | 2625 | 2750628 | 1047.86 |
| grpDM.bin.132 | 89.56 | 2.85 | 10742 | 1909 | 1636836 | 857.43 |
| grpDM.bin.133 | 94.79 | 1.03 | 27312 | 1980 | 1834230 | 926.38 |
| grpDM.bin.138 | 87.58 | 0 | 15881 | 1442 | 1320456 | 915.71 |
| grpDM.bin.139 | 93.92 | 1.98 | 36010 | 1832 | 1921632 | 1048.93 |
| grpDM.bin.14 | 99.26 | 1.47 | 66815 | 1963 | 1881471 | 958.47 |
| grpDM.bin.146 | 94.57 | 2.7 | 186663 | 3342 | 3711306 | 1110.5 |
| grpDM.bin.149 | 97.8 | 0.38 | 32639 | 1836 | 1922673 | 1047.21 |
| grpDM.bin.150 | 93.96 | 0.22 | 18801 | 1988 | 1809786 | 910.36 |
| grpDM.bin.151 | 94.52 | 0.16 | 24441 | 1539 | 1538004 | 999.35 |
| grpDM.bin.155 | 99.33 | 1.36 | 43304 | 2573 | 2584071 | 1004.3 |
| grpDM.bin.156 | 92.62 | 1.01 | 33151 | 1443 | 1389873 | 963.18 |
| grpDM.bin.157 | 88.34 | 3.02 | 22780 | 1226 | 1147392 | 935.88 |
| grpDM.bin.158 | 91.03 | 0.78 | 17352 | 1950 | 2025090 | 1038.51 |
| grpDM.bin.159 | 89.04 | 0 | 33379 | 1440 | 1371933 | 952.73 |
| grpDM.bin.16 | 82.52 | 1.68 | 17675 | 1735 | 1701510 | 980.7 |
| grpDM.bin.160 | 95.25 | 1.52 | 50530 | 2542 | 2496261 | 982.01 |
| grpDM.bin.164 | 94.56 | 0.59 | 33513 | 2130 | 2146260 | 1007.63 |
| grpDM.bin.169 | 84.87 | 0.71 | 7174 | 1785 | 1711998 | 959.1 |
| grpDM.bin.173 | 83.72 | 1.34 | 8873 | 1822 | 1635717 | 897.76 |
| grpDM.bin.174 | 97.9 | 0.5 | 99986 | 1887 | 2114331 | 1120.47 |
| grpDM.bin.180 | 94.85 | 0.67 | 34158 | 1597 | 1579065 | 988.77 |
| grpDM.bin.20 | 85.6 | 0 | 20764 | 2019 | 1907361 | 944.71 |
| grpDM.bin.200 | 85.4 | 6.2 | 16271 | 2412 | 2397117 | 993.83 |
| grpDM.bin.201 | 84.97 | 1.44 | 12824 | 1642 | 1495509 | 910.79 |
| grpDM.bin.202 | 92.34 | 0 | 57837 | 1243 | 1218255 | 980.09 |
| grpDM.bin.203 | 91.67 | 0.71 | 11593 | 2055 | 1868526 | 909.26 |
| grpDM.bin.206 | 96.18 | 0.84 | 34269 | 2386 | 2370591 | 993.54 |
| grpDM.bin.207 | 94.07 | 1.59 | 21261 | 2050 | 2079399 | 1014.34 |
| grpDM.bin.215 | 82.5 | 1.12 | 13394 | 2136 | 2147892 | 1005.57 |
| grpDM.bin.216 | 89.63 | 2.01 | 14017 | 1541 | 1404837 | 911.64 |
| grpDM.bin.218 | 85.73 | 7.29 | 14985 | 3009 | 2725290 | 905.71 |
| grpDM.bin.22 | 95.3 | 0.67 | 33175 | 1912 | 2085363 | 1090.67 |
| grpDM.bin.222 | 92.28 | 0 | 27270 | 1697 | 1600815 | 943.32 |
| grpDM.bin.225 | 80.8 | 0.84 | 18949 | 1670 | 1655391 | 991.25 |
| grpDM.bin.226 | 95.16 | 0.16 | 50096 | 1211 | 1247844 | 1030.42 |
| grpDM.bin.23 | 87.61 | 1.47 | 4870 | 1720 | 1474227 | 857.11 |
| grpDM.bin.230 | 92.52 | 0.34 | 37859 | 2047 | 2088528 | 1020.29 |
| grpDM.bin.232 | 97.33 | 1.33 | 62307 | 1837 | 1899402 | 1033.97 |
| grpDM.bin.233 | 87.12 | 2.35 | 43186 | 1953 | 1872090 | 958.57 |
| grpDM.bin.236 | 92.6 | 0.34 | 37387 | 2510 | 2569434 | 1023.68 |
| grpDM.bin.244 | 94.04 | 1.45 | 28161 | 2114 | 2100375 | 993.55 |
| grpDM.bin.253 | 89.98 | 0.28 | 12138 | 1298 | 1235832 | 952.1 |
| grpDM.bin.256 | 96.56 | 0 | 97169 | 1208 | 1182057 | 978.52 |
| grpDM.bin.258 | 92.71 | 1.01 | 72997 | 1748 | 1534035 | 877.59 |
| grpDM.bin.261 | 97.39 | 0 | 41934 | 1959 | 2037180 | 1039.91 |
| grpDM.bin.264 | 88.44 | 2.13 | 10494 | 1613 | 1399821 | 867.84 |
| grpDM.bin.269 | 97.55 | 0 | 146665 | 1843 | 1954557 | 1060.53 |
| grpDM.bin.27 | 97.43 | 0 | 125559 | 2301 | 2362569 | 1026.76 |
| grpDM.bin.274 | 93.1 | 0.34 | 40836 | 1967 | 2009355 | 1021.53 |
| grpDM.bin.283 | 85.54 | 5.82 | 19347 | 2406 | 2292273 | 952.73 |
| grpDM.bin.284 | 92.66 | 6.36 | 21449 | 2319 | 2581602 | 1113.24 |
| grpDM.bin.285 | 80.88 | 2.85 | 19686 | 2274 | 2126796 | 935.27 |
| grpDM.bin.29 | 95.05 | 1.57 | 15565 | 1630 | 1471782 | 902.93 |
| grpDM.bin.294 | 81.33 | 0 | 40815 | 1265 | 1312182 | 1037.3 |
| grpDM.bin.295 | 96.55 | 1.34 | 108628 | 2188 | 2212434 | 1011.17 |
| grpDM.bin.296 | 96.33 | 0 | 68339 | 1886 | 2138859 | 1134.07 |
| grpDM.bin.301 | 96.31 | 0.34 | 15919 | 2648 | 2344632 | 885.44 |
| grpDM.bin.308 | 93.95 | 0.81 | 29782 | 2663 | 2716617 | 1020.13 |
| grpDM.bin.31 | 91.57 | 0 | 103180 | 786 | 799206 | 1016.8 |
| grpDM.bin.310 | 99.04 | 0.24 | 72566 | 1843 | 1950141 | 1058.13 |
| grpDM.bin.314 | 92.38 | 2.54 | 17508 | 2123 | 2312631 | 1089.32 |
| grpDM.bin.318 | 96.64 | 0 | 62202 | 2347 | 2300802 | 980.32 |
| grpDM.bin.32 | 98.45 | 1.9 | 24477 | 2764 | 2751297 | 995.4 |
| grpDM.bin.33 | 88.16 | 1.08 | 22189 | 2019 | 1838742 | 910.72 |
| grpDM.bin.34 | 83.19 | 0.56 | 6460 | 1511 | 1361223 | 900.88 |
| grpDM.bin.37 | 87.98 | 1.23 | 6879 | 1927 | 1554078 | 806.48 |
| grpDM.bin.38 | 90.58 | 2.52 | 7352 | 2743 | 2643603 | 963.76 |
| grpDM.bin.39 | 81.62 | 0.43 | 32277 | 1611 | 1483074 | 920.59 |
| grpDM.bin.4 | 95.28 | 0.13 | 53438 | 2314 | 2424786 | 1047.88 |
| grpDM.bin.42 | 96.7 | 0.6 | 33635 | 1839 | 1897782 | 1031.96 |
| grpDM.bin.44 | 83.79 | 0.67 | 6014 | 1383 | 1128732 | 816.15 |
| grpDM.bin.48 | 94.88 | 0.67 | 32648 | 2089 | 2182434 | 1044.73 |
| grpDM.bin.5 | 87.68 | 3.9 | 25276 | 1950 | 1849440 | 948.43 |
| grpDM.bin.52 | 94.09 | 0 | 32190 | 1445 | 1374399 | 951.14 |
| grpDM.bin.6 | 96.63 | 0 | 54382 | 1521 | 1458390 | 958.84 |
| grpDM.bin.64 | 92.47 | 2.65 | 7374 | 1444 | 1183257 | 819.43 |
| grpDM.bin.67 | 86.57 | 2.24 | 7806 | 1622 | 1432728 | 883.31 |
| grpDM.bin.7 | 98.92 | 0.36 | 76154 | 1377 | 1290753 | 937.37 |
| grpDM.bin.71 | 87.75 | 0 | 19488 | 1961 | 1820697 | 928.45 |
| grpDM.bin.72 | 97.32 | 0.02 | 57239 | 2201 | 2229621 | 1013 |
| grpDM.bin.87 | 92.47 | 1.68 | 15557 | 1388 | 1273362 | 917.41 |
| grpDM.bin.89 | 88.95 | 0 | 4762 | 1335 | 1114305 | 834.69 |
| grpDM.bin.92 | 86.98 | 4.86 | 6832 | 1506 | 1391397 | 923.9 |
| grpDM.bin.93 | 83.89 | 7.61 | 9098 | 2273 | 1995531 | 877.93 |
| grpDM.bin.94 | 94.45 | 0.35 | 11791 | 3157 | 2914185 | 923.09 |
| grpDM.bin.96 | 94.57 | 2.7 | 27794 | 3771 | 4175922 | 1107.38 |
| grpH.bin.10 | 95.7 | 0 | 79560 | 1496 | 1423119 | 951.28 |
| grpH.bin.100 | 92.93 | 0.75 | 44095 | 1834 | 1990578 | 1085.38 |
| grpH.bin.102 | 93.27 | 0.89 | 30126 | 1842 | 1837821 | 997.73 |
| grpH.bin.122 | 97.41 | 0.37 | 115239 | 2136 | 2280465 | 1067.63 |
| grpH.bin.126 | 96.31 | 1.01 | 55145 | 1945 | 1936587 | 995.67 |
| grpH.bin.129 | 95.66 | 0.27 | 113816 | 2558 | 2745666 | 1073.36 |
| grpH.bin.132 | 84.42 | 1.25 | 18290 | 1954 | 1879872 | 962.06 |
| grpH.bin.135 | 92.65 | 4.28 | 16361 | 1913 | 1948917 | 1018.78 |
| grpH.bin.136 | 98.31 | 0.39 | 16510 | 1848 | 1841769 | 996.63 |
| grpH.bin.143 | 90.15 | 4.59 | 22831 | 2572 | 2672658 | 1039.14 |
| grpH.bin.152 | 97.07 | 0 | 59334 | 1437 | 1354881 | 942.85 |
| grpH.bin.156 | 95.7 | 0.54 | 81318 | 2094 | 2223840 | 1062.01 |
| grpH.bin.160 | 98.66 | 0 | 81627 | 2125 | 2155008 | 1014.12 |
| grpH.bin.171 | 85.12 | 1.34 | 11521 | 1626 | 1503486 | 924.65 |
| grpH.bin.175 | 96.63 | 1.12 | 65387 | 1522 | 1486359 | 976.58 |
| grpH.bin.18 | 95.24 | 0.5 | 50462 | 1845 | 2040234 | 1105.82 |
| grpH.bin.181 | 94.73 | 2.68 | 13654 | 1794 | 1677279 | 934.94 |
| grpH.bin.185 | 90.57 | 0.27 | 41394 | 2388 | 2020668 | 846.18 |
| grpH.bin.190 | 98.47 | 1.34 | 70645 | 2194 | 2179824 | 993.54 |
| grpH.bin.192 | 93.97 | 0.67 | 23068 | 2623 | 2464245 | 939.48 |
| grpH.bin.194 | 90.21 | 1.66 | 19191 | 1787 | 1725351 | 965.5 |
| grpH.bin.196 | 97.15 | 1.89 | 31994 | 2272 | 2374593 | 1045.16 |
| grpH.bin.20 | 92.05 | 0.02 | 64591 | 2065 | 2296851 | 1112.28 |
| grpH.bin.202 | 82.56 | 2.35 | 6848 | 1452 | 1280493 | 881.88 |
| grpH.bin.21 | 87.25 | 0 | 145609 | 1503 | 1393449 | 927.11 |
| grpH.bin.210 | 81.55 | 1.34 | 9893 | 1872 | 1799697 | 961.38 |
| grpH.bin.212 | 93.78 | 1.12 | 71937 | 1779 | 1901964 | 1069.12 |
| grpH.bin.218 | 97.65 | 0.67 | 46782 | 3057 | 3164652 | 1035.21 |
| grpH.bin.225 | 86.97 | 3.52 | 10193 | 2039 | 1955382 | 958.99 |
| grpH.bin.229 | 97.85 | 0 | 58097 | 1504 | 1371297 | 911.77 |
| grpH.bin.23 | 93.15 | 0 | 25365 | 1767 | 1797153 | 1017.06 |
| grpH.bin.236 | 80.01 | 0.67 | 12591 | 1669 | 1614807 | 967.53 |
| grpH.bin.237 | 94.41 | 1.07 | 31928 | 1884 | 1626543 | 863.35 |
| grpH.bin.238 | 93.7 | 1.43 | 28180 | 1969 | 1998066 | 1014.76 |
| grpH.bin.243 | 94.61 | 1.48 | 71517 | 2812 | 2960871 | 1052.94 |
| grpH.bin.245 | 84.68 | 1.61 | 21279 | 1474 | 1531032 | 1038.69 |
| grpH.bin.249 | 92.72 | 4.14 | 7368 | 3579 | 3213456 | 897.86 |
| grpH.bin.256 | 94.38 | 2.78 | 29724 | 3004 | 3028047 | 1008 |
| grpH.bin.266 | 87.79 | 0.84 | 20282 | 1961 | 2023002 | 1031.62 |
| grpH.bin.271 | 94.9 | 1.55 | 82190 | 2120 | 2279595 | 1075.28 |
| grpH.bin.275 | 93.36 | 0 | 23311 | 1840 | 1981776 | 1077.05 |
| grpH.bin.276 | 98.91 | 0 | 92104 | 2472 | 2504499 | 1013.15 |
| grpH.bin.28 | 80.91 | 2.31 | 6863 | 1674 | 1403775 | 838.58 |
| grpH.bin.281 | 91.77 | 1.61 | 14707 | 1609 | 1527048 | 949.07 |
| grpH.bin.288 | 98.9 | 1.1 | 99351 | 2439 | 2603538 | 1067.46 |
| grpH.bin.294 | 92.06 | 0.71 | 26097 | 1175 | 984237 | 837.65 |
| grpH.bin.298 | 96.06 | 1.34 | 65017 | 1999 | 1986990 | 993.99 |
| grpH.bin.302 | 80.4 | 1.89 | 13821 | 1833 | 1942275 | 1059.62 |
| grpH.bin.31 | 89.73 | 1.8 | 127452 | 2114 | 2352987 | 1113.05 |
| grpH.bin.310 | 90.54 | 2.03 | 16098 | 1895 | 1913775 | 1009.91 |
| grpH.bin.319 | 91.22 | 0.34 | 18476 | 1455 | 1397013 | 960.15 |
| grpH.bin.32 | 92.06 | 0 | 12580 | 1645 | 1650660 | 1003.44 |
| grpH.bin.323 | 86.39 | 0.34 | 4895 | 2577 | 2181252 | 846.43 |
| grpH.bin.324 | 95.79 | 0 | 61283 | 2166 | 2132850 | 984.7 |
| grpH.bin.36 | 88.28 | 0.94 | 34174 | 1733 | 1833642 | 1058.07 |
| grpH.bin.39 | 89.42 | 2.18 | 14104 | 1862 | 1638333 | 879.88 |
| grpH.bin.42 | 94.88 | 0 | 64597 | 1840 | 2003679 | 1088.96 |
| grpH.bin.46 | 91.94 | 2.58 | 8277 | 1932 | 1703844 | 881.91 |
| grpH.bin.50 | 81.15 | 1.34 | 21525 | 1639 | 1504521 | 917.95 |
| grpH.bin.53 | 98.22 | 0 | 90245 | 1634 | 1716342 | 1050.39 |
| grpH.bin.57 | 95.63 | 0.12 | 117422 | 2610 | 2990454 | 1145.77 |
| grpH.bin.65 | 94.74 | 0.67 | 35833 | 1939 | 1959630 | 1010.64 |
| grpH.bin.66 | 97.37 | 0.24 | 66343 | 1688 | 1805232 | 1069.45 |
| grpH.bin.71 | 99.04 | 0.72 | 163170 | 1944 | 2063721 | 1061.58 |
| grpH.bin.74 | 84.44 | 0.38 | 24853 | 1452 | 1620261 | 1115.88 |
| grpH.bin.80 | 89.28 | 3.3 | 6355 | 1900 | 1500657 | 789.82 |
| grpH.bin.81 | 80.21 | 4.06 | 9948 | 1079 | 1003254 | 929.8 |
| grpH.bin.84 | 87.38 | 0.96 | 7503 | 3147 | 3132063 | 995.25 |
| grpH.bin.95 | 83.28 | 3.96 | 8507 | 1858 | 1571394 | 845.74 |
| grpH.bin.98 | 84.65 | 2.18 | 13505 | 1635 | 1480482 | 905.49 |
| grpH.bin.99 | 97.32 | 0 | 93203 | 2483 | 2399661 | 966.44 |
| H1.bin.10 | 96.6 | 0.67 | 22071 | 1972 | 1859271 | 942.84 |
| H1.bin.101 | 95.97 | 2.01 | 39111 | 1977 | 1852272 | 936.91 |
| H1.bin.103 | 92.06 | 0.67 | 21773 | 2222 | 2049996 | 922.59 |
| H1.bin.108 | 95.51 | 0 | 50508 | 1483 | 1503648 | 1013.92 |
| H1.bin.109 | 95.08 | 2.57 | 13813 | 1595 | 1488255 | 933.08 |
| H1.bin.113 | 98.11 | 1.62 | 35466 | 3552 | 3830967 | 1078.54 |
| H1.bin.114 | 92.13 | 0 | 41306 | 1546 | 1414656 | 915.04 |
| H1.bin.117 | 97.04 | 0.54 | 87947 | 1986 | 2172363 | 1093.84 |
| H1.bin.120 | 100 | 0 | 90290 | 2434 | 2682333 | 1102.03 |
| H1.bin.125 | 95.97 | 0.67 | 31247 | 1939 | 2006256 | 1034.69 |
| H1.bin.13 | 95.97 | 0.81 | 119285 | 1608 | 1573014 | 978.24 |
| H1.bin.15 | 99.08 | 0.59 | 22595 | 2109 | 2051412 | 972.69 |
| H1.bin.16 | 94.1 | 0.67 | 10081 | 2015 | 1962450 | 973.92 |
| H1.bin.17 | 93.79 | 1.92 | 25857 | 1984 | 1828626 | 921.69 |
| H1.bin.2 | 98 | 0.84 | 32951 | 2169 | 2083053 | 960.37 |
| H1.bin.20 | 83.53 | 0.98 | 5383 | 2338 | 2085678 | 892.08 |
| H1.bin.21 | 94.01 | 0 | 48855 | 1978 | 2088519 | 1055.87 |
| H1.bin.22 | 99.33 | 0 | 42875 | 2990 | 2733042 | 914.06 |
| H1.bin.23 | 97.32 | 0 | 49186 | 2073 | 1969740 | 950.19 |
| H1.bin.24 | 87.82 | 0.28 | 4296 | 1363 | 1088358 | 798.5 |
| H1.bin.25 | 94.53 | 1.53 | 43844 | 2686 | 2796195 | 1041.03 |
| H1.bin.26 | 90.12 | 2.85 | 17279 | 1397 | 1313997 | 940.58 |
| H1.bin.29 | 97.99 | 0 | 90696 | 2291 | 2227344 | 972.21 |
| H1.bin.35 | 91.01 | 1.52 | 11088 | 2951 | 3100788 | 1050.76 |
| H1.bin.36 | 85.65 | 2.1 | 3715 | 2034 | 1617630 | 795.29 |
| H1.bin.38 | 87.13 | 0.81 | 29670 | 2338 | 2172801 | 929.34 |
| H1.bin.4 | 96.42 | 0.67 | 21305 | 2818 | 2586603 | 917.89 |
| H1.bin.40 | 95.95 | 1.43 | 30391 | 2179 | 2409834 | 1105.94 |
| H1.bin.43 | 85.97 | 0 | 86471 | 1748 | 1984836 | 1135.49 |
| H1.bin.44 | 96.64 | 0 | 115878 | 1777 | 1703391 | 958.58 |
| H1.bin.46 | 80.49 | 1.26 | 5865 | 1309 | 1078341 | 823.79 |
| H1.bin.50 | 100 | 0 | 184766 | 2333 | 2306235 | 988.53 |
| H1.bin.53 | 92.46 | 0 | 82073 | 1672 | 1812927 | 1084.29 |
| H1.bin.54 | 96.14 | 0.68 | 79407 | 2437 | 2329485 | 955.88 |
| H1.bin.60 | 88.59 | 1.45 | 10755 | 1678 | 1659573 | 989.02 |
| H1.bin.62 | 89.74 | 0.85 | 71113 | 1788 | 1690638 | 945.55 |
| H1.bin.64 | 82.48 | 3.7 | 4401 | 1923 | 1644156 | 855 |
| H1.bin.7 | 91.38 | 0.56 | 7269 | 2295 | 2198511 | 957.96 |
| H1.bin.77 | 99.51 | 0 | 95173 | 1604 | 1458837 | 909.5 |
| H1.bin.79 | 93.26 | 0 | 38590 | 1247 | 1196184 | 959.25 |
| H1.bin.8 | 83.26 | 1.67 | 4921 | 1857 | 1636221 | 881.11 |
| H1.bin.80 | 95.65 | 1.21 | 78938 | 2361 | 2413359 | 1022.18 |
| H1.bin.81 | 96.3 | 0.69 | 26801 | 3333 | 3626256 | 1087.99 |
| H1.bin.82 | 95.3 | 0.02 | 50637 | 2100 | 2116425 | 1007.82 |
| H1.bin.85 | 85.76 | 2.95 | 4286 | 2575 | 2221059 | 862.55 |
| H1.bin.89 | 96.42 | 0.69 | 50699 | 2207 | 2131782 | 965.92 |
| H1.bin.91 | 97.36 | 0.88 | 62215 | 2404 | 2648802 | 1101.83 |
| H1.bin.94 | 85.04 | 1.23 | 5949 | 1727 | 1481007 | 857.56 |
| H1.bin.95 | 91.85 | 2.01 | 29693 | 1968 | 1826994 | 928.35 |
| H1.bin.96 | 96.2 | 0.19 | 22338 | 2318 | 2454324 | 1058.81 |
| H1.bin.97 | 81.5 | 0 | 5278 | 2131 | 1668867 | 783.14 |
| H1.bin.98 | 85.93 | 1.79 | 26646 | 1738 | 1633797 | 940.04 |
| H2.bin.1 | 83.08 | 3.36 | 3593 | 1808 | 1528611 | 845.47 |
| H2.bin.10 | 89.5 | 2.46 | 9028 | 1872 | 1524549 | 814.4 |
| H2.bin.105 | 98.66 | 4.7 | 50057 | 2291 | 2240328 | 977.88 |
| H2.bin.106 | 93.55 | 2.04 | 24914 | 1667 | 1716354 | 1029.61 |
| H2.bin.109 | 80.64 | 3.82 | 9866 | 2005 | 1812147 | 903.81 |
| H2.bin.11 | 94.05 | 1.4 | 35833 | 1890 | 1840542 | 973.83 |
| H2.bin.113 | 92.68 | 2.38 | 15607 | 2262 | 2400279 | 1061.13 |
| H2.bin.115 | 95.58 | 0.22 | 47501 | 1862 | 1882590 | 1011.06 |
| H2.bin.116 | 91.84 | 0 | 57101 | 2061 | 2435064 | 1181.5 |
| H2.bin.117 | 87.92 | 0.34 | 37605 | 2014 | 1940211 | 963.36 |
| H2.bin.118 | 93.74 | 1.34 | 228718 | 2815 | 2819298 | 1001.53 |
| H2.bin.12 | 95.87 | 0.6 | 92487 | 2389 | 2712696 | 1135.49 |
| H2.bin.127 | 94.68 | 0 | 50259 | 2045 | 1952079 | 954.56 |
| H2.bin.128 | 80.9 | 4.59 | 39319 | 1038 | 897297 | 864.45 |
| H2.bin.13 | 95.08 | 1.24 | 16676 | 1476 | 1309596 | 887.26 |
| H2.bin.132 | 80.77 | 2.15 | 18667 | 2111 | 2016873 | 955.41 |
| H2.bin.133 | 82.59 | 1.35 | 25944 | 2370 | 2370990 | 1000.42 |
| H2.bin.18 | 97.3 | 0.67 | 16652 | 1977 | 1882695 | 952.3 |
| H2.bin.19 | 80.51 | 3.84 | 3330 | 1802 | 1526958 | 847.37 |
| H2.bin.20 | 97.99 | 0 | 65559 | 2246 | 2191050 | 975.53 |
| H2.bin.26 | 98.08 | 0.48 | 441011 | 1641 | 1718247 | 1047.07 |
| H2.bin.28 | 84.44 | 2.16 | 9146 | 2243 | 1996650 | 890.17 |
| H2.bin.29 | 93.1 | 1.55 | 33714 | 2178 | 2067768 | 949.39 |
| H2.bin.30 | 95.41 | 1.07 | 19287 | 2619 | 2616900 | 999.2 |
| H2.bin.32 | 90.13 | 2.07 | 9013 | 1386 | 1247070 | 899.76 |
| H2.bin.34 | 84.64 | 0 | 17511 | 1561 | 1494324 | 957.29 |
| H2.bin.35 | 84.62 | 0.34 | 40954 | 1288 | 1343235 | 1042.88 |
| H2.bin.36 | 89.48 | 4.92 | 10772 | 2252 | 1955322 | 868.26 |
| H2.bin.4 | 98.41 | 0.6 | 81564 | 2293 | 2274744 | 992.04 |
| H2.bin.40 | 99.06 | 1.29 | 163014 | 1734 | 1576713 | 909.29 |
| H2.bin.43 | 94.26 | 0.13 | 55639 | 1710 | 1548474 | 905.54 |
| H2.bin.44 | 98.4 | 0.18 | 36291 | 1562 | 1394457 | 892.74 |
| H2.bin.46 | 83.09 | 2.22 | 11454 | 1872 | 1781754 | 951.79 |
| H2.bin.48 | 97.99 | 0.81 | 24098 | 2414 | 2256012 | 934.55 |
| H2.bin.53 | 98.32 | 0.67 | 99925 | 2634 | 2536803 | 963.1 |
| H2.bin.56 | 97.32 | 1.34 | 58454 | 2329 | 2573853 | 1105.13 |
| H2.bin.57 | 84.71 | 0.49 | 6832 | 2254 | 2155101 | 956.12 |
| H2.bin.59 | 89.45 | 7.04 | 9332 | 1761 | 1521369 | 863.92 |
| H2.bin.6 | 92.2 | 0.02 | 69363 | 1873 | 1976778 | 1055.41 |
| H2.bin.7 | 85.19 | 3.85 | 5791 | 1852 | 1516716 | 818.96 |
| H2.bin.71 | 92.38 | 1.21 | 12475 | 1293 | 1246947 | 964.38 |
| H2.bin.73 | 94.63 | 0 | 62229 | 2290 | 2169786 | 947.5 |
| H2.bin.75 | 93.73 | 1.93 | 61403 | 2662 | 2575446 | 967.49 |
| H2.bin.76 | 93.14 | 0.63 | 57961 | 2433 | 2439807 | 1002.8 |
| H2.bin.79 | 80.88 | 3.19 | 11112 | 1879 | 1627992 | 866.41 |
| H2.bin.8 | 97.8 | 1.5 | 47268 | 3021 | 2943501 | 974.35 |
| H2.bin.80 | 98.16 | 1.06 | 124837 | 1894 | 1973220 | 1041.83 |
| H2.bin.81 | 90.48 | 0 | 23968 | 2091 | 2151291 | 1028.83 |
| H2.bin.84 | 93.09 | 1.17 | 53536 | 1656 | 1584423 | 956.78 |
| H2.bin.86 | 97.37 | 0.48 | 58395 | 1735 | 1869510 | 1077.53 |
| H2.bin.87 | 83.63 | 1.7 | 6521 | 1781 | 1486062 | 834.4 |
| H2.bin.89 | 93.06 | 0 | 26792 | 1722 | 1562739 | 907.51 |
| H2.bin.90 | 88.87 | 0.67 | 18420 | 1916 | 1836861 | 958.7 |
| H2.bin.95 | 94.17 | 8.77 | 30195 | 3604 | 3397074 | 942.58 |
| H3.bin.102 | 98.06 | 0.23 | 39631 | 1753 | 1643640 | 937.62 |
| H3.bin.104 | 98.6 | 0.7 | 177387 | 2146 | 2254524 | 1050.57 |
| H3.bin.107 | 90.19 | 6.54 | 8237 | 2859 | 2584311 | 903.92 |
| H3.bin.117 | 87.07 | 2.91 | 9173 | 2511 | 2465067 | 981.71 |
| H3.bin.119 | 95.98 | 2.13 | 116637 | 2462 | 2352150 | 955.38 |
| H3.bin.121 | 94.85 | 1.57 | 13352 | 2044 | 1983348 | 970.33 |
| H3.bin.125 | 95.08 | 0.67 | 41424 | 1922 | 2050029 | 1066.61 |
| H3.bin.127 | 94.95 | 1.61 | 10744 | 2964 | 2633013 | 888.33 |
| H3.bin.128 | 84.95 | 0.89 | 12655 | 2999 | 2373912 | 791.57 |
| H3.bin.130 | 96.42 | 0 | 82495 | 1704 | 1739187 | 1020.65 |
| H3.bin.134 | 92.74 | 3.09 | 28853 | 2355 | 2257089 | 958.42 |
| H3.bin.135 | 81.59 | 0.79 | 49563 | 1913 | 1951449 | 1020.1 |
| H3.bin.14 | 84.98 | 1.01 | 38903 | 2055 | 2141718 | 1042.2 |
| H3.bin.150 | 91.59 | 1.37 | 17909 | 2035 | 2218638 | 1090.24 |
| H3.bin.154 | 85.85 | 0.75 | 25767 | 1742 | 1919451 | 1101.87 |
| H3.bin.156 | 95.74 | 1.42 | 18131 | 1862 | 1742064 | 935.59 |
| H3.bin.157 | 84.83 | 5.47 | 8930 | 2536 | 2435715 | 960.46 |
| H3.bin.158 | 94.97 | 1.57 | 34526 | 2333 | 2220663 | 951.85 |
| H3.bin.161 | 90.11 | 0.55 | 13382 | 1346 | 1161837 | 863.18 |
| H3.bin.162 | 97.5 | 0.49 | 54053 | 3636 | 3664545 | 1007.85 |
| H3.bin.163 | 93.01 | 2.19 | 18620 | 2658 | 2594661 | 976.17 |
| H3.bin.166 | 96.74 | 0 | 17197 | 2771 | 2431152 | 877.36 |
| H3.bin.168 | 93.26 | 3.93 | 15859 | 1789 | 1414458 | 790.64 |
| H3.bin.172 | 98.6 | 0.7 | 96015 | 2249 | 2323323 | 1033.05 |
| H3.bin.19 | 82.85 | 3.5 | 24498 | 2733 | 2692218 | 985.08 |
| H3.bin.21 | 80.19 | 1.73 | 3736 | 2000 | 1464147 | 732.07 |
| H3.bin.23 | 93.62 | 1.21 | 10579 | 1300 | 1155486 | 888.84 |
| H3.bin.26 | 96.4 | 0.71 | 20263 | 1665 | 1686264 | 1012.77 |
| H3.bin.28 | 82.37 | 3.03 | 5064 | 1842 | 1631319 | 885.62 |
| H3.bin.30 | 97.99 | 0.22 | 56894 | 2526 | 2401458 | 950.7 |
| H3.bin.34 | 94.3 | 0 | 54332 | 1805 | 1659624 | 919.46 |
| H3.bin.36 | 95.08 | 1.12 | 21234 | 2176 | 2162604 | 993.84 |
| H3.bin.39 | 87.54 | 1.34 | 10448 | 2076 | 1890453 | 910.62 |
| H3.bin.4 | 94.76 | 0 | 50833 | 1815 | 1965771 | 1083.07 |
| H3.bin.43 | 90.15 | 3.25 | 28633 | 1914 | 1794225 | 937.42 |
| H3.bin.45 | 84.4 | 0.34 | 42622 | 2716 | 2809788 | 1034.53 |
| H3.bin.50 | 99.43 | 0.38 | 168004 | 2045 | 2171364 | 1061.79 |
| H3.bin.53 | 94.05 | 0.75 | 22426 | 1709 | 1779765 | 1041.41 |
| H3.bin.54 | 92.76 | 0.5 | 13403 | 1868 | 1744392 | 933.83 |
| H3.bin.56 | 87.48 | 2.07 | 14721 | 2018 | 1957089 | 969.82 |
| H3.bin.57 | 95.65 | 0.56 | 98904 | 2300 | 2316642 | 1007.24 |
| H3.bin.6 | 85.81 | 2.03 | 8949 | 2483 | 2242647 | 903.2 |
| H3.bin.60 | 98.62 | 0.63 | 34259 | 2186 | 2032476 | 929.77 |
| H3.bin.61 | 95.75 | 2.03 | 50363 | 2033 | 1959993 | 964.09 |
| H3.bin.62 | 93.51 | 0.02 | 64920 | 1603 | 1653666 | 1031.61 |
| H3.bin.65 | 87.64 | 3.23 | 12525 | 2327 | 1776771 | 763.55 |
| H3.bin.68 | 88.58 | 2.13 | 7493 | 3979 | 3931182 | 987.98 |
| H3.bin.7 | 98.15 | 0.19 | 46650 | 2462 | 2560599 | 1040.05 |
| H3.bin.70 | 92.51 | 1.08 | 33608 | 2173 | 2163198 | 995.49 |
| H3.bin.8 | 99.52 | 0.48 | 113275 | 1665 | 1803657 | 1083.28 |
| H3.bin.81 | 93.21 | 0.56 | 21964 | 1782 | 1907418 | 1070.38 |
| H3.bin.83 | 92.08 | 3.04 | 27675 | 2647 | 2517726 | 951.16 |
| H3.bin.84 | 99.33 | 1.03 | 38836 | 2568 | 2400921 | 934.94 |
| H3.bin.85 | 84.75 | 2.26 | 15209 | 2033 | 2048529 | 1007.64 |
| H3.bin.88 | 81.5 | 5.19 | 32234 | 1815 | 1513842 | 834.07 |
| H3.bin.89 | 95.17 | 0 | 17674 | 1783 | 1762728 | 988.63 |
| H3.bin.90 | 84.82 | 3.52 | 10064 | 1772 | 1735992 | 979.68 |
| H3.bin.92 | 94.08 | 0.29 | 30622 | 1765 | 1783539 | 1010.5 |
| H3.bin.95 | 80.7 | 1.74 | 4336 | 1407 | 1152972 | 819.45 |
| H3.bin.97 | 91.73 | 2.36 | 10671 | 2524 | 2557563 | 1013.3 |
