# Supplementary material for: Effects of diarrhea and antibiotic-induced microbial elimination on dynamic changes in fecal microbial communities and antibiotic resistance of Hu sheep lambs (Ovis aries)
Source: PeerJ. 2026 Jul 31;14:e21574. doi: 10.7717/peerj.21574 (PMC13431306; doi:10.7717/peerj.21574)
Supplement: Supplemental Information 4 [file peerj-14-21574-s004.docx]

| Supplementary Table S3. The taxonomy labels of MAGs annotated by GTDB-tk. | | | | | | | | | | | |
| --- | --- | --- | --- | --- | --- | --- | --- | --- | --- | --- | --- |
| MAGs | classification | Kingdom | Phylum | Class | Order | Family | Genus | Species | Closest genome reference | | Closest genome ANI |
| DL1.bin.17 | d__Bacteria;p__Bacteroidota;c__Bacteroidia;o__Bacteroidales;f__Bacteroidaceae;g__Phocaeicola;s__Phocaeicola dorei | Bacteria | Bacteroidota | Bacteroidia | Bacteroidales | Bacteroidaceae | *Phocaeicola* | *Phocaeicola dorei* | GCF_013009555.1 | | 98.86 |
| DL1.bin.23 | d__Bacteria;p__Bacillota;c__Clostridia;o__Lachnospirales;f__Lachnospiraceae;g__Mediterraneibacter;s__Mediterraneibacter faecigallinarum | Bacteria | Bacillota | Clostridia | Lachnospirales | Lachnospiraceae | *Mediterraneibacter* | *Mediterraneibacter faecigallinarum* | GCA_019119735.1 | | 98.81 |
| DL1.bin.28 | d__Bacteria;p__Bacillota;c__Bacilli;o__Erysipelotrichales;f__Erysipelotrichaceae;g__Amedibacterium;s__ | Bacteria | Bacillota | Bacilli | Erysipelotrichales | Erysipelotrichaceae | *Amedibacterium* | | N/A |  | N/A |
| DL1.bin.35 | d__Bacteria;p__Bacillota;c__Clostridia;o__Lachnospirales;f__Lachnospiraceae;g__Otoolea;s__Otoolea symbiosa | Bacteria | Bacillota | Clostridia | Lachnospirales | Lachnospiraceae | *Otoolea* | *Otoolea symbiosa* | GCF_000466485.1 | | 99.01 |
| DL2.bin.13 | d__Bacteria;p__Bacillota;c__Clostridia;o__Christensenellales;f__Borkfalkiaceae;g__Borkfalkia;s__Borkfalkia ceftriaxoniphila | Bacteria | Bacillota | Clostridia | Christensenellales | Borkfalkiaceae | *Borkfalkia* | *Borkfalkia ceftriaxoniphila* | GCF_004134775.1 | | 99.08 |
| DL2.bin.18 | d__Bacteria;p__Bacillota;c__Clostridia;o__Oscillospirales;f__Ruminococcaceae;g__Ruthenibacterium;s__Ruthenibacterium lactatiformans | Bacteria | Bacillota | Clostridia | Oscillospirales | Ruminococcaceae | *Ruthenibacterium* | *Ruthenibacterium lactatiformans* | GCF_000949455.1 | | 99.31 |
| DL2.bin.21 | d__Bacteria;p__Bacillota;c__Clostridia;o__Oscillospirales;f__Acutalibacteraceae;g__Hominenteromicrobium;s__Hominenteromicrobium sp004555625 | Bacteria | Bacillota | Clostridia | Oscillospirales | Acutalibacteraceae | *Hominenteromicrobium* | *Hominenteromicrobium sp004555625* | GCF_021531715.1 | | 96.67 |
| DL2.bin.22 | d__Bacteria;p__Bacillota;c__Clostridia;o__Christensenellales;f__CAG-917;g__CAG-349;s__CAG-349 sp003539515 | Bacteria | Bacillota | Clostridia | Christensenellales | CAG-917 | *CAG-349* | *CAG-349 sp003539515* | GCA_022009995.1 | | 97.24 |
| DL2.bin.26 | d__Bacteria;p__Bacillota;c__Clostridia;o__Oscillospirales;f__Acutalibacteraceae;g__Limousia;s__Limousia pullorum | Bacteria | Bacillota | Clostridia | Oscillospirales | Acutalibacteraceae | *Limousia* | *Limousia pullorum* | GCA_018714545.1 | | 96.33 |
| DL2.bin.28 | d__Bacteria;p__Bacillota;c__Clostridia;o__Lachnospirales;f__Lachnospiraceae;g__Mediterraneibacter;s__Mediterraneibacter vanvlietii | Bacteria | Bacillota | Clostridia | Lachnospirales | Lachnospiraceae | *Mediterraneibacter* | *Mediterraneibacter vanvlietii* | GCA_019113645.1 | | 98.6 |
| DL2.bin.29 | d__Bacteria;p__Bacillota;c__Clostridia;o__Oscillospirales;f__Ruminococcaceae;g__Fournierella;s__Fournierella merdipullorum | Bacteria | Bacillota | Clostridia | Oscillospirales | Ruminococcaceae | *Fournierella* | *Fournierella merdipullorum* | GCA_019116345.1 | | 97.11 |
| DL2.bin.3 | d__Bacteria;p__Bacillota;c__Clostridia;o__Peptostreptococcales;f__Peptostreptococcaceae;g__Terrisporobacter;s__Terrisporobacter othiniensis | Bacteria | Bacillota | Clostridia | Peptostreptococcales | Peptostreptococcaceae | *Terrisporobacter* | *Terrisporobacter othiniensis* | GCF_000808015.1 | | 99.69 |
| DL2.bin.30 | d__Bacteria;p__Verrucomicrobiota;c__Verrucomicrobiia;o__Verrucomicrobiales;f__Akkermansiaceae;g__Akkermansia;s__Akkermansia muciniphila | Bacteria | Verrucomicrobiota | Verrucomicrobiia | Verrucomicrobiales | Akkermansiaceae | *Akkermansia* | *Akkermansia muciniphila* | GCF_000020225.1 | | 98.9 |
| DL2.bin.33 | d__Bacteria;p__Bacteroidota;c__Bacteroidia;o__Bacteroidales;f__Bacteroidaceae;g__Bacteroides;s__Bacteroides uniformis | Bacteria | Bacteroidota | Bacteroidia | Bacteroidales | Bacteroidaceae | *Bacteroides* | *Bacteroides uniformis* | GCF_025147485.1 | | 98.22 |
| DL2.bin.36 | d__Bacteria;p__Bacillota;c__Clostridia;o__Lachnospirales;f__Lachnospiraceae;g__Enterocloster;s__Enterocloster lavalensis | Bacteria | Bacillota | Clostridia | Lachnospirales | Lachnospiraceae | *Enterocloster* | *Enterocloster lavalensis* | GCF_003024655.1 | | 98.97 |
| DL2.bin.38 | d__Bacteria;p__Bacteroidota;c__Bacteroidia;o__Bacteroidales;f__Rikenellaceae;g__Alistipes_A;s__Alistipes_A indistinctus | Bacteria | Bacteroidota | Bacteroidia | Bacteroidales | Rikenellaceae | *Alistipes_A* | *Alistipes_A indistinctus* | GCF_025144995.1 | | 99 |
| DL2.bin.6 | d__Bacteria;p__Bacillota;c__Clostridia;o__Clostridiales;f__Clostridiaceae;g__Clostridium_X;s__Clostridium_X cadaveris | Bacteria | Bacillota | Clostridia | Clostridiales | Clostridiaceae | *Clostridium_X* | *Clostridium_X cadaveris* | GCF_039521325.1 | | 99.6 |
| DL3.bin.10 | d__Bacteria;p__Bacillota;c__Clostridia;o__Oscillospirales;f__Oscillospiraceae;g__Flavonifractor;s__Flavonifractor plautii | Bacteria | Bacillota | Clostridia | Oscillospirales | Oscillospiraceae | *Flavonifractor* | *Flavonifractor plautii* | GCF_000239295.1 | | 98.69 |
| DL3.bin.24 | d__Bacteria;p__Bacteroidota;c__Bacteroidia;o__Bacteroidales;f__Bacteroidaceae;g__Bacteroides;s__Bacteroides xylanisolvens | Bacteria | Bacteroidota | Bacteroidia | Bacteroidales | Bacteroidaceae | *Bacteroides* | *Bacteroides xylanisolvens* | GCF_000210075.1 | | 97.44 |
| DL3.bin.25 | d__Bacteria;p__Bacillota;c__Clostridia;o__Oscillospirales;f__Acutalibacteraceae;g__UMGS1071;s__UMGS1071 sp900542375 | Bacteria | Bacillota | Clostridia | Oscillospirales | Acutalibacteraceae | *UMGS1071* | *UMGS1071 sp900542375* | GCA_900542375.1 | | 98.99 |
| DL3.bin.35 | d__Bacteria;p__Bacillota;c__Bacilli;o__Erysipelotrichales;f__Coprobacillaceae;g__Thomasclavelia;s__Thomasclavelia ramosa | Bacteria | Bacillota | Bacilli | Erysipelotrichales | Coprobacillaceae | *Thomasclavelia* | *Thomasclavelia ramosa* | GCF_014131695.1 | | 99.57 |
| DL3.bin.38 | d__Bacteria;p__Pseudomonadota;c__Gammaproteobacteria;o__Burkholderiales;f__Burkholderiaceae;g__Parasutterella;s__Parasutterella sp900552195 | Bacteria | Pseudomonadota | Gammaproteobacteria | Burkholderiales | Burkholderiaceae | *Parasutterella* | *Parasutterella sp900552195* | GCA_002492145.1 | | 98.67 |
| DL3.bin.40 | d__Bacteria;p__Bacillota;c__Clostridia;o__Lachnospirales;f__Lachnospiraceae;g__Enterocloster;s__Enterocloster bolteae | Bacteria | Bacillota | Clostridia | Lachnospirales | Lachnospiraceae | *Enterocloster* | *Enterocloster bolteae* | GCF_002234575.2 | | 97.4 |
| DL3.bin.6 | d__Bacteria;p__Bacillota;c__Clostridia;o__Christensenellales;f__Borkfalkiaceae;g__Borkfalkia;s__Borkfalkia avicola | Bacteria | Bacillota | Clostridia | Christensenellales | Borkfalkiaceae | *Borkfalkia* | *Borkfalkia avicola* | GCA_019116115.1 | | 97.97 |
| DL3.bin.7 | d__Bacteria;p__Bacillota;c__Clostridia;o__Lachnospirales;f__Lachnospiraceae;g__Oliverpabstia;s__Oliverpabstia sp034114925 | Bacteria | Bacillota | Clostridia | Lachnospirales | Lachnospiraceae | *Oliverpabstia* | *Oliverpabstia sp034114925* | GCA_034114925.1 | | 96.29 |
| DM1.bin.10 | d__Bacteria;p__Bacillota;c__Clostridia;o__Lachnospirales;f__Lachnospiraceae;g__CAG-303;s__ | Bacteria | Bacillota | Clostridia | Lachnospirales | Lachnospiraceae | *CAG-303* |  | N/A |  | N/A |
| DM1.bin.102 | d__Bacteria;p__Bacteroidota;c__Bacteroidia;o__Bacteroidales;f__Tannerellaceae;g__Parabacteroides;s__ | Bacteria | Bacteroidota | Bacteroidia | Bacteroidales | Tannerellaceae | *Parabacteroides* | | N/A |  | N/A |
| DM1.bin.104 | d__Bacteria;p__Bacillota;c__Clostridia;o__Oscillospirales;f__Oscillospiraceae;g__Faecousia;s__ | Bacteria | Bacillota | Clostridia | Oscillospirales | Oscillospiraceae | *Faecousia* |  | N/A |  | N/A |
| DM1.bin.105 | d__Bacteria;p__Bacillota;c__Clostridia;o__Oscillospirales;f__CAG-382;g__UMGS1052;s__UMGS1052 sp015063785 | Bacteria | Bacillota | Clostridia | Oscillospirales | CAG-382 | *UMGS1052* | *UMGS1052 sp015063785* | GCA_015063785.1 | | 97.87 |
| DM1.bin.107 | d__Bacteria;p__Bacillota;c__Clostridia;o__Christensenellales;f__Aristaeellaceae;g__UBA11524;s__UBA11524 sp900769075 | Bacteria | Bacillota | Clostridia | Christensenellales | Aristaeellaceae | *UBA11524* | *UBA11524 sp900769075* | GCA_900769075.1 | | 97.59 |
| DM1.bin.112 | d__Bacteria;p__Spirochaetota;c__Spirochaetia;o__Treponematales;f__Treponemataceae;g__Treponema_D;s__Treponema_D sp905236565 | Bacteria | Spirochaetota | Spirochaetia | Treponematales | Treponemataceae | *Treponema_D* | *Treponema_D sp905236565* | GCF_905236565.1 | | 98.45 |
| DM1.bin.118 | d__Bacteria;p__Bacillota;c__Clostridia;o__Oscillospirales;f__Acutalibacteraceae;g__UBA6857;s__ | Bacteria | Bacillota | Clostridia | Oscillospirales | Acutalibacteraceae | *UBA6857* | | N/A |  | N/A |
| DM1.bin.12 | d__Bacteria;p__Bacteroidota;c__Bacteroidia;o__Bacteroidales;f__UBA932;g__Cryptobacteroides;s__Cryptobacteroides sp900545245 | Bacteria | Bacteroidota | Bacteroidia | Bacteroidales | UBA932 | *Cryptobacteroides* | *Cryptobacteroides sp900545245* | GCA_900545245.1 | | 97.21 |
| DM1.bin.127 | d__Bacteria;p__Bacillota;c__Clostridia;o__Oscillospirales;f__CAG-272;g__UMGS1002;s__UMGS1002 sp900547565 | Bacteria | Bacillota | Clostridia | Oscillospirales | CAG-272 | *UMGS1002* | *UMGS1002 sp900547565* | GCA_900547565.1 | | 97.39 |
| DM1.bin.13 | d__Bacteria;p__Bacteroidota;c__Bacteroidia;o__Bacteroidales;f__Bacteroidaceae;g__Prevotella;s__Prevotella sp004554665 | Bacteria | Bacteroidota | Bacteroidia | Bacteroidales | Bacteroidaceae | *Prevotella* | *Prevotella sp004554665* | GCA_004554665.1 | | 98.77 |
| DM1.bin.131 | d__Bacteria;p__Bacteroidota;c__Bacteroidia;o__Bacteroidales;f__Bacteroidaceae;g__Phocaeicola;s__Phocaeicola sp017416735 | Bacteria | Bacteroidota | Bacteroidia | Bacteroidales | Bacteroidaceae | *Phocaeicola* | *Phocaeicola sp017416735* | GCA_017416735.1 | | 97.08 |
| DM1.bin.133 | d__Bacteria;p__Bacillota;c__Clostridia;o__Lachnospirales;f__Anaerotignaceae;g__Anaerotignum_A;s__Anaerotignum_A sp945912105 | Bacteria | Bacillota | Clostridia | Lachnospirales | Anaerotignaceae | *Anaerotignum_A* | *Anaerotignum_A sp945912105* | GCA_945912105.1 | | 98.26 |
| DM1.bin.134 | d__Bacteria;p__Bacillota;c__Clostridia;o__Oscillospirales;f__Oscillospiraceae;g__Faecousia;s__ | Bacteria | Bacillota | Clostridia | Oscillospirales | Oscillospiraceae | *Faecousia* |  | N/A |  | N/A |
| DM1.bin.139 | d__Bacteria;p__Bacillota;c__Clostridia;o__Oscillospirales;f__CAG-272;g__RGIG2000;s__ | Bacteria | Bacillota | Clostridia | Oscillospirales | CAG-272 | *RGIG2000* | | N/A |  | N/A |
| DM1.bin.141 | d__Bacteria;p__Bacteroidota;c__Bacteroidia;o__Bacteroidales;f__Muribaculaceae;g__CAG-873;s__ | Bacteria | Bacteroidota | Bacteroidia | Bacteroidales | Muribaculaceae | *CAG-873* |  | N/A |  | N/A |
| DM1.bin.22 | d__Bacteria;p__Bacillota;c__Clostridia;o__Christensenellales;f__CAG-314;g__Heteroclostridium;s__ | Bacteria | Bacillota | Clostridia | Christensenellales | CAG-314 | *Heteroclostridium* | | N/A |  | N/A |
| DM1.bin.38 | d__Bacteria;p__Bacteroidota;c__Bacteroidia;o__Bacteroidales;f__Bacteroidaceae;g__Prevotella;s__ | Bacteria | Bacteroidota | Bacteroidia | Bacteroidales | Bacteroidaceae | *Prevotella* | | N/A |  | N/A |
| DM1.bin.39 | d__Bacteria;p__Bacillota;c__Clostridia;o__Oscillospirales;f__CAG-272;g__Flemingiibacterium;s__ | Bacteria | Bacillota | Clostridia | Oscillospirales | CAG-272 | *Flemingiibacterium* | | N/A |  | N/A |
| DM1.bin.44 | d__Bacteria;p__Bacillota;c__Clostridia;o__Oscillospirales;f__CAG-272;g__SIG701;s__ | Bacteria | Bacillota | Clostridia | Oscillospirales | CAG-272 | *SIG701* |  | N/A |  | N/A |
| DM1.bin.54 | d__Bacteria;p__Bacillota;c__Clostridia;o__Lachnospirales;f__Lachnospiraceae;g__SIG333;s__SIG333 sp021153185 | Bacteria | Bacillota | Clostridia | Lachnospirales | Lachnospiraceae | *SIG333* | *SIG333 sp021153185* | GCA_021153185.1 | | 98.86 |
| DM1.bin.60 | d__Bacteria;p__Bacteroidota;c__Bacteroidia;o__Bacteroidales;f__Bacteroidaceae;g__Minthosoma;s__Minthosoma sp900318775 | Bacteria | Bacteroidota | Bacteroidia | Bacteroidales | Bacteroidaceae | *Minthosoma* | *Minthosoma sp900318775* | GCA_900318775.1 | | 97.65 |
| DM1.bin.62 | d__Bacteria;p__Bacillota;c__Clostridia;o__Oscillospirales;f__Oscillospiraceae;g__F23-B02;s__F23-B02 sp945613045 | Bacteria | Bacillota | Clostridia | Oscillospirales | Oscillospiraceae | *F23-B02* | *F23-B02 sp945613045* | GCA_945613045.1 | | 98.75 |
| DM1.bin.68 | d__Bacteria;p__Bacillota;c__Clostridia;o__Oscillospirales;f__Acutalibacteraceae;g__RUG420;s__RUG420 sp900317085 | Bacteria | Bacillota | Clostridia | Oscillospirales | Acutalibacteraceae | *RUG420* | *RUG420 sp900317085* | GCA_900317085.1 | | 98.77 |
| DM1.bin.71 | d__Bacteria;p__Spirochaetota;c__Spirochaetia;o__Treponematales;f__Treponemataceae;g__Treponema_D;s__Treponema_D sp945873775 | Bacteria | Spirochaetota | Spirochaetia | Treponematales | Treponemataceae | *Treponema_D* | *Treponema_D sp945873775* | GCF_945873775.1 | | 98.63 |
| DM1.bin.73 | d__Bacteria;p__Bacillota;c__Clostridia;o__Christensenellales;f__Borkfalkiaceae;g__Scatosoma;s__Scatosoma sp017626135 | Bacteria | Bacillota | Clostridia | Christensenellales | Borkfalkiaceae | *Scatosoma* | *Scatosoma sp017626135* | GCA_017626135.1 | | 98.62 |
| DM1.bin.75 | d__Bacteria;p__Bacteroidota;c__Bacteroidia;o__Bacteroidales;f__Paludibacteraceae;g__UBA1723;s__UBA1723 sp002371265 | Bacteria | Bacteroidota | Bacteroidia | Bacteroidales | Paludibacteraceae | *UBA1723* | *UBA1723 sp002371265* | GCA_002371265.1 | | 95.3 |
| DM1.bin.77 | d__Bacteria;p__Bacillota;c__Clostridia;o__Oscillospirales;f__Oscillospiraceae;g__Vescimonas;s__ | Bacteria | Bacillota | Clostridia | Oscillospirales | Oscillospiraceae | *Vescimonas* | | N/A |  | N/A |
| DM1.bin.78 | d__Bacteria;p__Bacillota;c__Clostridia;o__Oscillospirales;f__CAG-272;g__Firm-07;s__ | Bacteria | Bacillota | Clostridia | Oscillospirales | CAG-272 | *Firm-07* |  | N/A |  | N/A |
| DM1.bin.83 | d__Bacteria;p__Bacteroidota;c__Bacteroidia;o__Bacteroidales;f__Muribaculaceae;g__CAG-873;s__ | Bacteria | Bacteroidota | Bacteroidia | Bacteroidales | Muribaculaceae | *CAG-873* |  | N/A |  | N/A |
| DM1.bin.85 | d__Bacteria;p__Bacteroidota;c__Bacteroidia;o__Bacteroidales;f__Bacteroidaceae;g__HGM04593;s__HGM04593 sp017467015 | Bacteria | Bacteroidota | Bacteroidia | Bacteroidales | Bacteroidaceae | *HGM04593* | *HGM04593 sp017467015* | GCA_017467015.1 | | 98.75 |
| DM1.bin.98 | d__Bacteria;p__Bacillota;c__Clostridia;o__Lachnospirales;f__Lachnospiraceae;g__Agathobacter;s__ | Bacteria | Bacillota | Clostridia | Lachnospirales | Lachnospiraceae | *Agathobacter* | | N/A |  | N/A |
| DM2.bin.100 | d__Bacteria;p__Bacillota;c__Clostridia;o__Christensenellales;f__Aristaeellaceae;g__SFMI01;s__ | Bacteria | Bacillota | Clostridia | Christensenellales | Aristaeellaceae | *SFMI01* |  | N/A |  | N/A |
| DM2.bin.104 | d__Bacteria;p__Bacteroidota;c__Bacteroidia;o__Bacteroidales;f__Bacteroidaceae;g__HGM04593;s__HGM04593 sp937906165 | Bacteria | Bacteroidota | Bacteroidia | Bacteroidales | Bacteroidaceae | *HGM04593* | *HGM04593 sp937906165* | GCA_937906165.1 | | 97.41 |
| DM2.bin.105 | d__Bacteria;p__Bacteroidota;c__Bacteroidia;o__Bacteroidales;f__UBA932;g__Cryptobacteroides;s__Cryptobacteroides sp900546445 | Bacteria | Bacteroidota | Bacteroidia | Bacteroidales | UBA932 | *Cryptobacteroides* | *Cryptobacteroides sp900546445* | GCA_900546445.1 | | 98.68 |
| DM2.bin.113 | d__Bacteria;p__Bacillota;c__Clostridia;o__Oscillospirales;f__UBA644;g__HGM12619;s__HGM12619 sp017621705 | Bacteria | Bacillota | Clostridia | Oscillospirales | UBA644 | *HGM12619* | *HGM12619 sp017621705* | GCA_017621705.1 | | 97.67 |
| DM2.bin.121 | d__Bacteria;p__Bacillota;c__Clostridia;o__Oscillospirales;f__Oscillospiraceae;g__Faecousia;s__ | Bacteria | Bacillota | Clostridia | Oscillospirales | Oscillospiraceae | *Faecousia* |  | N/A |  | N/A |
| DM2.bin.18 | d__Bacteria;p__Bacillota;c__Clostridia;o__Oscillospirales;f__Acutalibacteraceae;g__RUG420;s__RUG420 sp900317085 | Bacteria | Bacillota | Clostridia | Oscillospirales | Acutalibacteraceae | *RUG420* | *RUG420 sp900317085* | GCA_900317085.1 | | 98.59 |
| DM2.bin.23 | d__Bacteria;p__Bacteroidota;c__Bacteroidia;o__Bacteroidales;f__Bacteroidaceae;g__Prevotella;s__Prevotella ruminicola | Bacteria | Bacteroidota | Bacteroidia | Bacteroidales | Bacteroidaceae | *Prevotella* | *Prevotella ruminicola* | GCF_000025925.1 | | 97.16 |
| DM2.bin.28 | d__Bacteria;p__Bacillota;c__Clostridia;o__Oscillospirales;f__Acutalibacteraceae;g__SIG565;s__ | Bacteria | Bacillota | Clostridia | Oscillospirales | Acutalibacteraceae | *SIG565* |  | N/A |  | N/A |
| DM2.bin.36 | d__Bacteria;p__Bacillota;c__Clostridia;o__Oscillospirales;f__Acutalibacteraceae;g__DTU089;s__DTU089 sp017937865 | Bacteria | Bacillota | Clostridia | Oscillospirales | Acutalibacteraceae | *DTU089* | *DTU089 sp017937865* | GCA_017937865.1 | | 97.53 |
| DM2.bin.50 | d__Bacteria;p__Bacillota;c__Clostridia;o__Lachnospirales;f__Anaerotignaceae;g__Anaerotignum_A;s__Anaerotignum_A sp945912105 | Bacteria | Bacillota | Clostridia | Lachnospirales | Anaerotignaceae | *Anaerotignum_A* | *Anaerotignum_A sp945912105* | GCA_945912105.1 | | 98.21 |
| DM2.bin.52 | d__Bacteria;p__Bacillota;c__Clostridia;o__Oscillospirales;f__Acutalibacteraceae;g__DTU089;s__DTU089 sp017626295 | Bacteria | Bacillota | Clostridia | Oscillospirales | Acutalibacteraceae | *DTU089* | *DTU089 sp017626295* | GCA_017626295.1 | | 97.21 |
| DM2.bin.54 | d__Bacteria;p__Bacillota;c__Clostridia;o__Oscillospirales;f__Acutalibacteraceae;g__DTU089;s__DTU089 sp017621885 | Bacteria | Bacillota | Clostridia | Oscillospirales | Acutalibacteraceae | *DTU089* | *DTU089 sp017621885* | GCA_017621885.1 | | 99.16 |
| DM2.bin.61 | d__Bacteria;p__Bacillota;c__Clostridia;o__Oscillospirales;f__CAG-272;g__CAG-448;s__ | Bacteria | Bacillota | Clostridia | Oscillospirales | CAG-272 | *CAG-448* |  | N/A |  | N/A |
| DM2.bin.66 | d__Bacteria;p__Bacillota;c__Clostridia;o__Oscillospirales;f__CAG-272;g__CAG-272;s__ | Bacteria | Bacillota | Clostridia | Oscillospirales | CAG-272 | *CAG-272* |  | N/A |  | N/A |
| DM2.bin.72 | d__Bacteria;p__Campylobacterota;c__Campylobacteria;o__Campylobacterales;f__Campylobacteraceae;g__Campylobacter;s__Campylobacter vicugnae | Bacteria | Campylobacterota | Campylobacteria | Campylobacterales | Campylobacteraceae | *Campylobacter* | *Campylobacter vicugnae* | GCF_002139875.1 | | 97.48 |
| DM2.bin.81 | d__Bacteria;p__Bacillota;c__Clostridia;o__Lachnospirales;f__Lachnospiraceae;g__RGIG8767;s__ | Bacteria | Bacillota | Clostridia | Lachnospirales | Lachnospiraceae | *RGIG8767* | | N/A |  | N/A |
| DM2.bin.83 | d__Bacteria;p__Bacteroidota;c__Bacteroidia;o__Bacteroidales;f__Bacteroidaceae;g__Colenecus;s__Colenecus sp900318995 | Bacteria | Bacteroidota | Bacteroidia | Bacteroidales | Bacteroidaceae | *Colenecus* | *Colenecus sp900318995* | GCA_900318995.1 | | 95.85 |
| DM2.bin.84 | d__Bacteria;p__Bacteroidota;c__Bacteroidia;o__Bacteroidales;f__Rikenellaceae;g__Alistipes;s__Alistipes sp017474235 | Bacteria | Bacteroidota | Bacteroidia | Bacteroidales | Rikenellaceae | *Alistipes* | *Alistipes sp017474235* | GCA_017474235.1 | | 97.63 |
| DM2.bin.94 | d__Bacteria;p__Bacillota;c__Clostridia;o__Oscillospirales;f__Ruminococcaceae;g__SIG578;s__ | Bacteria | Bacillota | Clostridia | Oscillospirales | Ruminococcaceae | *SIG578* |  | N/A |  | N/A |
| DM2.bin.95 | d__Bacteria;p__Bacillota;c__Clostridia;o__Christensenellales;f__CAG-314;g__Heteroclostridium;s__ | Bacteria | Bacillota | Clostridia | Christensenellales | CAG-314 | *Heteroclostridium* | | N/A |  | N/A |
| DM3.bin.1 | d__Bacteria;p__Bacillota;c__Clostridia;o__Oscillospirales;f__Acutalibacteraceae;g__SIG588;s__ | Bacteria | Bacillota | Clostridia | Oscillospirales | Acutalibacteraceae | *SIG588* |  | N/A |  | N/A |
| DM3.bin.100 | d__Bacteria;p__Bacillota;c__Clostridia;o__Oscillospirales;f__Acutalibacteraceae;g__UMGS1976;s__ | Bacteria | Bacillota | Clostridia | Oscillospirales | Acutalibacteraceae | *UMGS1976* | | N/A |  | N/A |
| DM3.bin.101 | d__Bacteria;p__Bacillota;c__Clostridia;o__Oscillospirales;f__CAG-272;g__RGIG3925;s__ | Bacteria | Bacillota | Clostridia | Oscillospirales | CAG-272 | *RGIG3925* | | N/A |  | N/A |
| DM3.bin.102 | d__Bacteria;p__Bacillota;c__Clostridia;o__Oscillospirales;f__CAG-272;g__CAG-390;s__CAG-390 sp003523225 | Bacteria | Bacillota | Clostridia | Oscillospirales | CAG-272 | *CAG-390* | *CAG-390 sp003523225* | GCA_902461815.1 | | 98.54 |
| DM3.bin.104 | d__Bacteria;p__Bacillota;c__Clostridia;o__Oscillospirales;f__Acutalibacteraceae;g__UBA1081;s__ | Bacteria | Bacillota | Clostridia | Oscillospirales | Acutalibacteraceae | *UBA1081* | | N/A |  | N/A |
| DM3.bin.106 | d__Bacteria;p__Bacillota;c__Clostridia;o__Oscillospirales;f__CAG-272;g__CAG-448;s__ | Bacteria | Bacillota | Clostridia | Oscillospirales | CAG-272 | *CAG-448* |  | N/A |  | N/A |
| DM3.bin.110 | d__Bacteria;p__Verrucomicrobiota;c__Kiritimatiellia;o__RFP12;f__UBA1067;g__UBA1067;s__ | Bacteria | Verrucomicrobiota | Kiritimatiellia | RFP12 | UBA1067 | *UBA1067* | | N/A |  | N/A |
| DM3.bin.111 | d__Bacteria;p__Bacillota;c__Clostridia;o__Oscillospirales;f__CAG-272;g__Firm-07;s__ | Bacteria | Bacillota | Clostridia | Oscillospirales | CAG-272 | *Firm-07* |  | N/A |  | N/A |
| DM3.bin.113 | d__Bacteria;p__Campylobacterota;c__Campylobacteria;o__Campylobacterales;f__Campylobacteraceae;g__Campylobacter;s__Campylobacter sp017506845 | Bacteria | Campylobacterota | Campylobacteria | Campylobacterales | Campylobacteraceae | *Campylobacter* | *Campylobacter sp017506845* | GCA_017506845.1 | | 96.7 |
| DM3.bin.118 | d__Bacteria;p__Bacillota;c__Clostridia;o__Oscillospirales;f__Butyricicoccaceae;g__UBA4644;s__UBA4644 sp029099685 | Bacteria | Bacillota | Clostridia | Oscillospirales | Butyricicoccaceae | *UBA4644* | *UBA4644 sp029099685* | GCA_029099685.1 | | 96.5 |
| DM3.bin.12 | d__Bacteria;p__Cyanobacteriota;c__Vampirovibrionia;o__Gastranaerophilales;f__Gastranaerophilaceae;g__UMGS1585;s__ | Bacteria | Cyanobacteriota | Vampirovibrionia | Gastranaerophilales | Gastranaerophilaceae | *UMGS1585* | | N/A |  | N/A |
| DM3.bin.122 | d__Bacteria;p__Bacteroidota;c__Bacteroidia;o__Bacteroidales;f__Bacteroidaceae;g__Physcousia;s__ | Bacteria | Bacteroidota | Bacteroidia | Bacteroidales | Bacteroidaceae | *Physcousia* | | N/A |  | N/A |
| DM3.bin.124 | d__Bacteria;p__Elusimicrobiota;c__Elusimicrobia;o__Elusimicrobiales;f__Elusimicrobiaceae;g__Avelusimicrobium;s__ | Bacteria | Elusimicrobiota | Elusimicrobia | Elusimicrobiales | Elusimicrobiaceae | *Avelusimicrobium* | | N/A |  | N/A |
| DM3.bin.128 | d__Bacteria;p__Bacillota;c__Bacilli;o__ML615J-28;f__CAG-313;g__UMGS75;s__UMGS75 sp934140285 | Bacteria | Bacillota | Bacilli | ML615J-28 | CAG-313 | *UMGS75* | *UMGS75 sp934140285* | GCA_934140285.1 | | 98.85 |
| DM3.bin.129 | d__Bacteria;p__Bacillota;c__Clostridia;o__Christensenellales;f__Borkfalkiaceae;g__Scatosoma;s__Scatosoma sp017626135 | Bacteria | Bacillota | Clostridia | Christensenellales | Borkfalkiaceae | *Scatosoma* | *Scatosoma sp017626135* | GCA_017626135.1 | | 98.78 |
| DM3.bin.20 | d__Bacteria;p__Bacillota;c__Clostridia;o__Lachnospirales;f__Lachnospiraceae;g__HGM12587;s__HGM12587 sp945786935 | Bacteria | Bacillota | Clostridia | Lachnospirales | Lachnospiraceae | *HGM12587* | *HGM12587 sp945786935* | GCA_945786935.1 | | 97.12 |
| DM3.bin.21 | d__Bacteria;p__Bacillota;c__Clostridia;o__Lachnospirales;f__Lachnospiraceae;g__RGIG989;s__RGIG989 sp017390525 | Bacteria | Bacillota | Clostridia | Lachnospirales | Lachnospiraceae | *RGIG989* | *RGIG989 sp017390525* | GCA_017390525.1 | | 99.06 |
| DM3.bin.23 | d__Bacteria;p__Pseudomonadota;c__Alphaproteobacteria;o__RF32;f__CAG-239;g__MGBC133411;s__MGBC133411 sp900540425 | Bacteria | Pseudomonadota | Alphaproteobacteria | RF32 | CAG-239 | *MGBC133411* | *MGBC133411 sp900540425* | GCA_900540425.1 | | 98.97 |
| DM3.bin.26 | d__Bacteria;p__Bacteroidota;c__Bacteroidia;o__Bacteroidales;f__Muribaculaceae;g__CAG-873;s__ | Bacteria | Bacteroidota | Bacteroidia | Bacteroidales | Muribaculaceae | *CAG-873* |  | N/A |  | N/A |
| DM3.bin.31 | d__Bacteria;p__Bacillota;c__Clostridia;o__Oscillospirales;f__Oscillospiraceae;g__Vescimonas;s__Vescimonas sp004557655 | Bacteria | Bacillota | Clostridia | Oscillospirales | Oscillospiraceae | *Vescimonas* | *Vescimonas sp004557655* | GCA_004557655.1 | | 97.18 |
| DM3.bin.33 | d__Bacteria;p__Bacillota;c__Clostridia;o__Oscillospirales;f__Oscillospiraceae;g__Faecousia;s__Faecousia sp945836055 | Bacteria | Bacillota | Clostridia | Oscillospirales | Oscillospiraceae | *Faecousia* | *Faecousia sp945836055* | GCA_945836055.1 | | 96.07 |
| DM3.bin.35 | d__Bacteria;p__Bacillota;c__Clostridia;o__Christensenellales;f__Borkfalkiaceae;g__HGM11412;s__HGM11412 sp017504795 | Bacteria | Bacillota | Clostridia | Christensenellales | Borkfalkiaceae | *HGM11412* | *HGM11412 sp017504795* | GCA_017504795.1 | | 97.1 |
| DM3.bin.36 | d__Bacteria;p__Bacillota;c__Clostridia;o__Oscillospirales;f__Ruminococcaceae;g__UMGS363;s__ | Bacteria | Bacillota | Clostridia | Oscillospirales | Ruminococcaceae | *UMGS363* | | N/A |  | N/A |
| DM3.bin.37 | d__Bacteria;p__Bacillota;c__Clostridia;o__Lachnospirales;f__Lachnospiraceae;g__CAG-510;s__ | Bacteria | Bacillota | Clostridia | Lachnospirales | Lachnospiraceae | *CAG-510* |  | N/A |  | N/A |
| DM3.bin.38 | d__Bacteria;p__Bacillota;c__Clostridia;o__Lachnospirales;f__Lachnospiraceae;g__Schaedlerella;s__Schaedlerella sp004556565 | Bacteria | Bacillota | Clostridia | Lachnospirales | Lachnospiraceae | *Schaedlerella* | *Schaedlerella sp004556565* | GCA_945830165.1 | | 98.7 |
| DM3.bin.43 | d__Bacteria;p__Bacteroidota;c__Bacteroidia;o__Bacteroidales;f__Muribaculaceae;g__Lepagella;s__ | Bacteria | Bacteroidota | Bacteroidia | Bacteroidales | Muribaculaceae | *Lepagella* |  | N/A |  | N/A |
| DM3.bin.44 | d__Bacteria;p__Bacillota;c__Clostridia;o__Oscillospirales;f__CAG-272;g__CAG-448;s__ | Bacteria | Bacillota | Clostridia | Oscillospirales | CAG-272 | *CAG-448* |  | N/A |  | N/A |
| DM3.bin.46 | d__Bacteria;p__Bacteroidota;c__Bacteroidia;o__Bacteroidales;f__UBA932;g__Cryptobacteroides;s__Cryptobacteroides sp000434935 | Bacteria | Bacteroidota | Bacteroidia | Bacteroidales | UBA932 | *Cryptobacteroides* | *Cryptobacteroides sp000434935* | GCA_000434935.1 | | 96.85 |
| DM3.bin.49 | d__Bacteria;p__Bacillota;c__Clostridia;o__Christensenellales;f__Borkfalkiaceae;g__UBA1259;s__UBA1259 sp934271245 | Bacteria | Bacillota | Clostridia | Christensenellales | Borkfalkiaceae | *UBA1259* | *UBA1259 sp934271245* | GCA_934271245.1 | | 97.33 |
| DM3.bin.5 | d__Bacteria;p__Bacillota;c__Clostridia;o__Oscillospirales;f__Acutalibacteraceae;g__Ruminococcoides;s__Ruminococcoides sp002350765 | Bacteria | Bacillota | Clostridia | Oscillospirales | Acutalibacteraceae | *Ruminococcoides* | *Ruminococcoides sp002350765* | GCA_002350765.1 | | 96.55 |
| DM3.bin.53 | d__Bacteria;p__Bacillota;c__Clostridia;o__Oscillospirales;f__Acutalibacteraceae;g__CAG-177;s__CAG-177 sp016295895 | Bacteria | Bacillota | Clostridia | Oscillospirales | Acutalibacteraceae | *CAG-177* | *CAG-177 sp016295895* | GCA_016295895.1 | | 97.94 |
| DM3.bin.55 | d__Bacteria;p__Bacillota;c__Clostridia;o__Christensenellales;f__CAG-314;g__Fimimonas;s__ | Bacteria | Bacillota | Clostridia | Christensenellales | CAG-314 | *Fimimonas* | | N/A |  | N/A |
| DM3.bin.56 | d__Bacteria;p__Bacteroidota;c__Bacteroidia;o__Bacteroidales;f__Bacteroidaceae;g__Colenecus;s__ | Bacteria | Bacteroidota | Bacteroidia | Bacteroidales | Bacteroidaceae | *Colenecus* | | N/A |  | N/A |
| DM3.bin.57 | d__Bacteria;p__Bacillota;c__Clostridia;o__Oscillospirales;f__Acutalibacteraceae;g__DTU089;s__DTU089 sp017937865 | Bacteria | Bacillota | Clostridia | Oscillospirales | Acutalibacteraceae | *DTU089* | *DTU089 sp017937865* | GCA_017937865.1 | | 97.66 |
| DM3.bin.58 | d__Bacteria;p__Bacteroidota;c__Bacteroidia;o__Bacteroidales;f__Bacteroidaceae;g__Minthosoma;s__Minthosoma sp017540585 | Bacteria | Bacteroidota | Bacteroidia | Bacteroidales | Bacteroidaceae | *Minthosoma* | *Minthosoma sp017540585* | GCA_017540585.1 | | 96.57 |
| DM3.bin.60 | d__Bacteria;p__Bacillota;c__Clostridia;o__Oscillospirales;f__Oscillospiraceae;g__Limivicinus;s__Limivicinus sp004560305 | Bacteria | Bacillota | Clostridia | Oscillospirales | Oscillospiraceae | *Limivicinus* | *Limivicinus sp004560305* | GCA_004560305.1 | | 96.79 |
| DM3.bin.61 | d__Bacteria;p__Bacillota;c__Clostridia;o__Christensenellales;f__Borkfalkiaceae;g__UBA4636;s__UBA4636 sp945638575 | Bacteria | Bacillota | Clostridia | Christensenellales | Borkfalkiaceae | *UBA4636* | *UBA4636 sp945638575* | GCA_945638575.1 | | 99.57 |
| DM3.bin.63 | d__Bacteria;p__Bacillota;c__Clostridia;o__Oscillospirales;f__Oscillospiraceae;g__CAG-170;s__CAG-170 sp004555655 | Bacteria | Bacillota | Clostridia | Oscillospirales | Oscillospiraceae | *CAG-170* | *CAG-170 sp004555655* | GCA_004555655.1 | | 95.65 |
| DM3.bin.67 | d__Bacteria;p__Bacillota;c__Clostridia;o__Peptostreptococcales;f__Anaerovoracaceae;g__Crickella;s__Crickella sp902778585 | Bacteria | Bacillota | Clostridia | Peptostreptococcales | Anaerovoracaceae | *Crickella* | *Crickella sp902778585* | GCA_902778585.1 | | 97.05 |
| DM3.bin.68 | d__Bacteria;p__Bacteroidota;c__Bacteroidia;o__Bacteroidales;f__Bacteroidaceae;g__Phocaeicola;s__Phocaeicola faecalis | Bacteria | Bacteroidota | Bacteroidia | Bacteroidales | Bacteroidaceae | *Phocaeicola* | *Phocaeicola faecalis* | GCF_021730445.1 | | 98.82 |
| DM3.bin.69 | d__Bacteria;p__Bacillota;c__Clostridia;o__Oscillospirales;f__Ruminococcaceae;g__Ruminococcus;s__Ruminococcus flavefaciens_A | Bacteria | Bacillota | Clostridia | Oscillospirales | Ruminococcaceae | *Ruminococcus* | *Ruminococcus flavefaciens_A* | GCA_000174895.1 | | 96.85 |
| DM3.bin.7 | d__Bacteria;p__Spirochaetota;c__Spirochaetia;o__Treponematales;f__Treponemataceae;g__Treponema_D;s__ | Bacteria | Spirochaetota | Spirochaetia | Treponematales | Treponemataceae | *Treponema_D* | | N/A |  | N/A |
| DM3.bin.70 | d__Bacteria;p__Bacteroidota;c__Bacteroidia;o__Bacteroidales;f__Bacteroidaceae;g__Prevotella;s__Prevotella sp900119285 | Bacteria | Bacteroidota | Bacteroidia | Bacteroidales | Bacteroidaceae | *Prevotella* | *Prevotella sp900119285* | GCA_900119285.1 | | 97.22 |
| DM3.bin.75 | d__Bacteria;p__Bacillota;c__Bacilli;o__RFN20;f__CAG-826;g__UBA2450;s__UBA2450 sp946625285 | Bacteria | Bacillota | Bacilli | RFN20 | CAG-826 | *UBA2450* | *UBA2450 sp946625285* | GCA_946625285.1 | | 95.6 |
| DM3.bin.76 | d__Bacteria;p__Bacillota;c__Clostridia;o__Christensenellales;f__CAG-917;g__CAG-349;s__CAG-349 sp003539515 | Bacteria | Bacillota | Clostridia | Christensenellales | CAG-917 | *CAG-349* | *CAG-349 sp003539515* | GCA_022009995.1 | | 96.89 |
| DM3.bin.78 | d__Bacteria;p__Bacillota;c__Clostridia;o__Oscillospirales;f__CAG-272;g__HGM12713;s__ | Bacteria | Bacillota | Clostridia | Oscillospirales | CAG-272 | *HGM12713* | | N/A |  | N/A |
| DM3.bin.8 | d__Bacteria;p__Spirochaetota;c__Spirochaetia;o__Treponematales;f__Treponemataceae;g__Treponema_D;s__Treponema_D sp017392945 | Bacteria | Spirochaetota | Spirochaetia | Treponematales | Treponemataceae | *Treponema_D* | *Treponema_D sp017392945* | GCA_017392945.1 | | 98.18 |
| DM3.bin.85 | d__Bacteria;p__Bacillota;c__Clostridia;o__Lachnospirales;f__Anaerotignaceae;g__Anaerotignum_A;s__Anaerotignum_A sp945912105 | Bacteria | Bacillota | Clostridia | Lachnospirales | Anaerotignaceae | *Anaerotignum_A* | *Anaerotignum_A sp945912105* | GCA_945912105.1 | | 98.09 |
| DM3.bin.87 | d__Bacteria;p__Bacillota;c__Clostridia;o__Oscillospirales;f__Acutalibacteraceae;g__RUG420;s__RUG420 sp900317085 | Bacteria | Bacillota | Clostridia | Oscillospirales | Acutalibacteraceae | *RUG420* | *RUG420 sp900317085* | GCA_900317085.1 | | 98.8 |
| DM3.bin.88 | d__Bacteria;p__Pseudomonadota;c__Alphaproteobacteria;o__Rs-D84;f__Rs-D84;g__Enterousia;s__ | Bacteria | Pseudomonadota | Alphaproteobacteria | Rs-D84 | Rs-D84 | *Enterousia* | | N/A |  | N/A |
| DM3.bin.89 | d__Bacteria;p__Bacillota;c__Clostridia;o__Oscillospirales;f__Acutalibacteraceae;g__Fimenecus;s__ | Bacteria | Bacillota | Clostridia | Oscillospirales | Acutalibacteraceae | *Fimenecus* | | N/A |  | N/A |
| DM3.bin.90 | d__Bacteria;p__Bacillota;c__Clostridia;o__Oscillospirales;f__Acutalibacteraceae;g__RGIG426;s__RGIG426 sp017410585 | Bacteria | Bacillota | Clostridia | Oscillospirales | Acutalibacteraceae | *RGIG426* | *RGIG426 sp017410585* | GCA_017410585.1 | | 97.69 |
| DM3.bin.91 | d__Bacteria;p__Bacteroidota;c__Bacteroidia;o__Bacteroidales;f__Bacteroidaceae;g__Prevotella;s__Prevotella sp002351725 | Bacteria | Bacteroidota | Bacteroidia | Bacteroidales | Bacteroidaceae | *Prevotella* | *Prevotella sp002351725* | GCA_002351725.1 | | 96.9 |
| grpDL.bin.1 | d__Bacteria;p__Bacillota;c__Clostridia;o__Oscillospirales;f__Ruminococcaceae;g__Gemmiger;s__Gemmiger sp905214345 | Bacteria | Bacillota | Clostridia | Oscillospirales | Ruminococcaceae | *Gemmiger* | *Gemmiger sp905214345* | GCA_905214345.1 | | 98.69 |
| grpDL.bin.12 | d__Bacteria;p__Bacillota;c__Clostridia;o__Oscillospirales;f__Oscillospiraceae;g__Oscillibacter;s__Oscillibacter sp900548505 | Bacteria | Bacillota | Clostridia | Oscillospirales | Oscillospiraceae | *Oscillibacter* | *Oscillibacter sp900548505* | GCA_900548505.1 | | 96 |
| grpDL.bin.13 | d__Bacteria;p__Bacillota;c__Clostridia;o__Lachnospirales;f__Lachnospiraceae;g__Claveliimonas;s__Claveliimonas bilis | Bacteria | Bacillota | Clostridia | Lachnospirales | Lachnospiraceae | *Claveliimonas* | *Claveliimonas bilis* | GCF_030296775.1 | | 98.73 |
| grpDL.bin.16 | d__Bacteria;p__Bacillota;c__Bacilli;o__Erysipelotrichales;f__Erysipelotrichaceae;g__CAMQRS01;s__ | Bacteria | Bacillota | Bacilli | Erysipelotrichales | Erysipelotrichaceae | *CAMQRS01* | | N/A |  | N/A |
| grpDL.bin.21 | d__Bacteria;p__Bacillota;c__Clostridia;o__Christensenellales;f__HGM11417;g__CAJMQN01;s__CAJMQN01 sp905215695 | Bacteria | Bacillota | Clostridia | Christensenellales | HGM11417 | *CAJMQN01* | *CAJMQN01 sp905215695* | GCA_905215695.1 | | 97.81 |
| grpDL.bin.24 | d__Bacteria;p__Bacteroidota;c__Bacteroidia;o__Bacteroidales;f__Bacteroidaceae;g__Bacteroides;s__Bacteroides cutis | Bacteria | Bacteroidota | Bacteroidia | Bacteroidales | Bacteroidaceae | *Bacteroides* | *Bacteroides cutis* | GCF_900241005.1 | | 99.61 |
| grpDL.bin.27 | d__Bacteria;p__Bacillota;c__Bacilli;o__Erysipelotrichales;f__Erysipelotrichaceae;g__Clostridium_AQ;s__Clostridium_AQ innocuum | Bacteria | Bacillota | Bacilli | Erysipelotrichales | Erysipelotrichaceae | *Clostridium_AQ* | *Clostridium_AQ innocuum* | GCA_012317185.1 | | 97.69 |
| grpDL.bin.28 | d__Bacteria;p__Bacillota;c__Clostridia;o__Oscillospirales;f__Acutalibacteraceae;g__Hydrogeniiclostridium;s__ | Bacteria | Bacillota | Clostridia | Oscillospirales | Acutalibacteraceae | *Hydrogeniiclostridium* | | N/A |  | N/A |
| grpDL.bin.3 | d__Bacteria;p__Bacillota;c__Clostridia;o__Oscillospirales;f__Ruminococcaceae;g__Neobittarella;s__Neobittarella massiliensis | Bacteria | Bacillota | Clostridia | Oscillospirales | Ruminococcaceae | *Neobittarella* | *Neobittarella massiliensis* | GCF_900289145.1 | | 98.53 |
| grpDL.bin.32 | d__Bacteria;p__Bacteroidota;c__Bacteroidia;o__Bacteroidales;f__Marinifilaceae;g__Butyricimonas;s__Butyricimonas virosa | Bacteria | Bacteroidota | Bacteroidia | Bacteroidales | Marinifilaceae | *Butyricimonas* | *Butyricimonas virosa* | GCF_025148635.1 | | 98.02 |
| grpDL.bin.37 | d__Bacteria;p__Bacillota;c__Clostridia;o__Oscillospirales;f__Acutalibacteraceae;g__Avimonas_A;s__Avimonas_A narfae | Bacteria | Bacillota | Clostridia | Oscillospirales | Acutalibacteraceae | *Avimonas_A* | *Avimonas_A narfae* | GCA_904395335.1 | | 98.18 |
| grpDL.bin.4 | d__Bacteria;p__Bacillota;c__Clostridia;o__Lachnospirales;f__Lachnospiraceae;g__Blautia;s__Blautia celeris | Bacteria | Bacillota | Clostridia | Lachnospirales | Lachnospiraceae | *Blautia* | *Blautia celeris* | GCF_014287615.1 | | 99.06 |
| grpDL.bin.44 | d__Bacteria;p__Bacillota;c__Clostridia;o__Peptostreptococcales;f__Anaerovoracaceae;g__Fimisoma;s__Fimisoma sp002320005 | Bacteria | Bacillota | Clostridia | Peptostreptococcales | Anaerovoracaceae | *Fimisoma* | *Fimisoma sp002320005* | GCA_002320005.1 | | 98.85 |
| grpDL.bin.47 | d__Bacteria;p__Bacillota;c__Clostridia;o__Oscillospirales;f__Oscillospiraceae;g__Intestinimonas;s__Intestinimonas butyriciproducens | Bacteria | Bacillota | Clostridia | Oscillospirales | Oscillospiraceae | *Intestinimonas* | *Intestinimonas butyriciproducens* | GCF_004154955.1 | | 99.29 |
| grpDL.bin.48 | d__Bacteria;p__Bacillota;c__Clostridia;o__Lachnospirales;f__Lachnospiraceae;g__Sellimonas;s__Sellimonas caecigallum | Bacteria | Bacillota | Clostridia | Lachnospirales | Lachnospiraceae | *Sellimonas* | *Sellimonas caecigallum* | GCF_019754295.1 | | 99 |
| grpDL.bin.49 | d__Bacteria;p__Bacillota;c__Clostridia;o__Oscillospirales;f__Butyricicoccaceae;g__Butyricicoccus;s__Butyricicoccus sp959018045 | Bacteria | Bacillota | Clostridia | Oscillospirales | Butyricicoccaceae | *Butyricicoccus* | *Butyricicoccus sp959018045* | GCA_959018045.1 | | 97.38 |
| grpDL.bin.51 | d__Bacteria;p__Bacillota;c__Clostridia;o__Lachnospirales;f__Lachnospiraceae;g__Mediterraneibacter;s__Mediterraneibacter stercorigallinarum | Bacteria | Bacillota | Clostridia | Lachnospirales | Lachnospiraceae | *Mediterraneibacter* | *Mediterraneibacter stercorigallinarum* | GCA_019116185.1 | | 98.86 |
| grpDL.bin.6 | d__Bacteria;p__Pseudomonadota;c__Gammaproteobacteria;o__Burkholderiales;f__Burkholderiaceae;g__Comamonas;s__Comamonas kerstersii | Bacteria | Pseudomonadota | Gammaproteobacteria | Burkholderiales | Burkholderiaceae | *Comamonas* | *Comamonas kerstersii* | GCF_008801935.1 | | 98.77 |
| grpDL.bin.62 | d__Bacteria;p__Bacillota;c__Clostridia;o__Oscillospirales;f__Ruminococcaceae;g__Pseudoruminococcus_B;s__ | Bacteria | Bacillota | Clostridia | Oscillospirales | Ruminococcaceae | *Pseudoruminococcus_B* | | N/A |  | N/A |
| grpDL.bin.63 | d__Bacteria;p__Actinomycetota;c__Coriobacteriia;o__Coriobacteriales;f__Eggerthellaceae;g__Eggerthella;s__Eggerthella lenta | Bacteria | Actinomycetota | Coriobacteriia | Coriobacteriales | Eggerthellaceae | *Eggerthella* | *Eggerthella lenta* | GCF_000024265.1 | | 98.02 |
| grpDL.bin.66 | d__Bacteria;p__Bacillota;c__Bacilli;o__Erysipelotrichales;f__Coprobacillaceae;g__Thomasclavelia;s__Thomasclavelia spiroformis | Bacteria | Bacillota | Bacilli | Erysipelotrichales | Coprobacillaceae | *Thomasclavelia* | *Thomasclavelia spiroformis* | GCF_025149465.1 | | 98.91 |
| grpDL.bin.7 | d__Bacteria;p__Bacillota;c__Clostridia;o__Oscillospirales;f__Butyricicoccaceae;g__Agathobaculum;s__Agathobaculum intestinigallinarum | Bacteria | Bacillota | Clostridia | Oscillospirales | Butyricicoccaceae | *Agathobaculum* | *Agathobaculum intestinigallinarum* | GCA_019119515.1 | | 97.44 |
| grpDM.bin.106 | d__Bacteria;p__Fibrobacterota;c__Fibrobacteria;o__Fibrobacterales;f__Fibrobacteraceae;g__Fibrobacter;s__Fibrobacter sp900313675 | Bacteria | Fibrobacterota | Fibrobacteria | Fibrobacterales | Fibrobacteraceae | *Fibrobacter* | *Fibrobacter sp900313675* | GCA_900313675.1 | | 96.41 |
| grpDM.bin.112 | d__Bacteria;p__Bacteroidota;c__Bacteroidia;o__Bacteroidales;f__UBA932;g__Cryptobacteroides;s__Cryptobacteroides sp000432655 | Bacteria | Bacteroidota | Bacteroidia | Bacteroidales | UBA932 | *Cryptobacteroides* | *Cryptobacteroides sp000432655* | GCA_902472425.1 | | 97.97 |
| grpDM.bin.115 | d__Bacteria;p__Bacillota;c__Clostridia;o__Christensenellales;f__Borkfalkiaceae;g__Scatosoma;s__ | Bacteria | Bacillota | Clostridia | Christensenellales | Borkfalkiaceae | *Scatosoma* | | N/A |  | N/A |
| grpDM.bin.116 | d__Bacteria;p__Bacillota;c__Clostridia;o__Oscillospirales;f__Acutalibacteraceae;g__Fimenecus;s__Fimenecus sp937897215 | Bacteria | Bacillota | Clostridia | Oscillospirales | Acutalibacteraceae | *Fimenecus* | *Fimenecus sp937897215* | GCA_937897215.1 | | 97.58 |
| grpDM.bin.119 | d__Bacteria;p__Bacillota;c__Clostridia;o__UMGS1810;f__UMGS1810;g__Qingrenia;s__Qingrenia sp022769845 | Bacteria | Bacillota | Clostridia | UMGS1810 | UMGS1810 | *Qingrenia* | *Qingrenia sp022769845* | GCA_022769845.1 | | 97.71 |
| grpDM.bin.123 | d__Bacteria;p__Bacillota;c__Clostridia;o__Lachnospirales;f__Lachnospiraceae;g__CAG-411;s__ | Bacteria | Bacillota | Clostridia | Lachnospirales | Lachnospiraceae | *CAG-411* |  | N/A |  | N/A |
| grpDM.bin.126 | d__Bacteria;p__Bacteroidota;c__Bacteroidia;o__Bacteroidales;f__Bacteroidaceae;g__Prevotella;s__Prevotella sp030539375 | Bacteria | Bacteroidota | Bacteroidia | Bacteroidales | Bacteroidaceae | *Prevotella* | *Prevotella sp030539375* | GCA_030539375.1 | | 96.13 |
| grpDM.bin.13 | d__Bacteria;p__Bacillota;c__Clostridia;o__Oscillospirales;f__CAG-272;g__UMGS1696;s__ | Bacteria | Bacillota | Clostridia | Oscillospirales | CAG-272 | *UMGS1696* | | N/A |  | N/A |
| grpDM.bin.132 | d__Bacteria;p__Bacillota;c__Clostridia;o__Oscillospirales;f__Oscillospiraceae;g__Vescimonas;s__ | Bacteria | Bacillota | Clostridia | Oscillospirales | Oscillospiraceae | *Vescimonas* | | N/A |  | N/A |
| grpDM.bin.133 | d__Bacteria;p__Bacillota;c__Clostridia;o__Oscillospirales;f__Acutalibacteraceae;g__CAG-177;s__CAG-177 sp003514385 | Bacteria | Bacillota | Clostridia | Oscillospirales | Acutalibacteraceae | *CAG-177* | *CAG-177 sp003514385* | GCA_003514385.1 | | 96.02 |
| grpDM.bin.138 | d__Bacteria;p__Bacillota;c__Clostridia;o__Monoglobales;f__Firm-18;g__UBA1775;s__ | Bacteria | Bacillota | Clostridia | Monoglobales | Firm-18 | *UBA1775* | | N/A |  | N/A |
| grpDM.bin.139 | d__Bacteria;p__Verrucomicrobiota;c__Verrucomicrobiia;o__Opitutales;f__Intestinicryptomonadaceae;g__Merdousia;s__Merdousia sp021641325 | Bacteria | Verrucomicrobiota | Verrucomicrobiia | Opitutales | Intestinicryptomonadaceae | *Merdousia* | *Merdousia sp021641325* | GCA_029269845.2 | | 98.28 |
| grpDM.bin.14 | d__Bacteria;p__Bacteroidota;c__Bacteroidia;o__Flavobacteriales;f__UBA1820;g__Merdimorpha;s__Merdimorpha sp003150615 | Bacteria | Bacteroidota | Bacteroidia | Flavobacteriales | UBA1820 | *Merdimorpha* | *Merdimorpha sp003150615* | GCA_003150615.1 | | 99.07 |
| grpDM.bin.146 | d__Bacteria;p__Verrucomicrobiota;c__Lentisphaeria;o__Victivallales;f__UBA1829;g__UBA1829;s__ | Bacteria | Verrucomicrobiota | Lentisphaeria | Victivallales | UBA1829 | *UBA1829* | | N/A |  | N/A |
| grpDM.bin.149 | d__Bacteria;p__Bacteroidota;c__Bacteroidia;o__Bacteroidales;f__Muribaculaceae;g__Paramuribaculum;s__Paramuribaculum sp900551515 | Bacteria | Bacteroidota | Bacteroidia | Bacteroidales | Muribaculaceae | *Paramuribaculum* | *Paramuribaculum sp900551515* | GCA_902489785.1 | | 99.04 |
| grpDM.bin.150 | d__Bacteria;p__Bacillota;c__Clostridia;o__Oscillospirales;f__Ruminococcaceae;g__CAG-115;s__CAG-115 sp017847835 | Bacteria | Bacillota | Clostridia | Oscillospirales | Ruminococcaceae | *CAG-115* | *CAG-115 sp017847835* | GCA_017847835.1 | | 97.95 |
| grpDM.bin.151 | d__Bacteria;p__Bacillota;c__Clostridia;o__Oscillospirales;f__CAG-272;g__UBA1740;s__ | Bacteria | Bacillota | Clostridia | Oscillospirales | CAG-272 | *UBA1740* | | N/A |  | N/A |
| grpDM.bin.155 | d__Bacteria;p__Bacillota;c__Clostridia;o__Lachnospirales;f__Lachnospiraceae;g__Falcatimonas;s__Falcatimonas sp018918265 | Bacteria | Bacillota | Clostridia | Lachnospirales | Lachnospiraceae | *Falcatimonas* | *Falcatimonas sp018918265* | GCF_018918265.1 | | 98.47 |
| grpDM.bin.156 | d__Bacteria;p__Bacillota;c__Clostridia;o__Oscillospirales;f__Acutalibacteraceae;g__RGIG2206;s__ | Bacteria | Bacillota | Clostridia | Oscillospirales | Acutalibacteraceae | *RGIG2206* | | N/A |  | N/A |
| grpDM.bin.157 | d__Bacteria;p__Bacillota;c__Clostridia;o__Oscillospirales;f__Acutalibacteraceae;g__UBA737;s__ | Bacteria | Bacillota | Clostridia | Oscillospirales | Acutalibacteraceae | *UBA737* |  | N/A |  | N/A |
| grpDM.bin.158 | d__Bacteria;p__Bacillota;c__Clostridia;o__Christensenellales;f__Borkfalkiaceae;g__Scatosoma;s__ | Bacteria | Bacillota | Clostridia | Christensenellales | Borkfalkiaceae | *Scatosoma* | | N/A |  | N/A |
| grpDM.bin.159 | d__Bacteria;p__Bacillota;c__Clostridia;o__Oscillospirales;f__Acutalibacteraceae;g__UMGS1279;s__ | Bacteria | Bacillota | Clostridia | Oscillospirales | Acutalibacteraceae | *UMGS1279* | | N/A |  | N/A |
| grpDM.bin.16 | d__Bacteria;p__Bacillota;c__Clostridia;o__Oscillospirales;f__CAG-382;g__Coliplasma;s__Coliplasma sp017516825 | Bacteria | Bacillota | Clostridia | Oscillospirales | CAG-382 | *Coliplasma* | *Coliplasma sp017516825* | GCA_017516825.1 | | 97.3 |
| grpDM.bin.160 | d__Bacteria;p__Bacillota;c__Clostridia;o__Lachnospirales;f__Lachnospiraceae;g__VSOB01;s__VSOB01 sp022779165 | Bacteria | Bacillota | Clostridia | Lachnospirales | Lachnospiraceae | *VSOB01* | *VSOB01 sp022779165* | GCA_022779165.1 | | 97.92 |
| grpDM.bin.164 | d__Bacteria;p__Desulfobacterota_I;c__Desulfovibrionia;o__Desulfovibrionales;f__Desulfovibrionaceae;g__Desulfovibrio;s__Desulfovibrio sp900556755 | Bacteria | Desulfobacterota_I | Desulfovibrionia | Desulfovibrionales | Desulfovibrionaceae | *Desulfovibrio* | *Desulfovibrio sp900556755* | GCF_951793255.1 | | 98.72 |
| grpDM.bin.169 | d__Bacteria;p__Bacteroidota;c__Bacteroidia;o__Bacteroidales;f__UBA932;g__Egerieousia;s__Egerieousia sp017935505 | Bacteria | Bacteroidota | Bacteroidia | Bacteroidales | UBA932 | *Egerieousia* | *Egerieousia sp017935505* | GCA_017935505.1 | | 97.74 |
| grpDM.bin.173 | d__Bacteria;p__Bacillota;c__Clostridia;o__HGM11327;f__HGM11327;g__SIG351;s__SIG351 sp017935525 | Bacteria | Bacillota | Clostridia | HGM11327 | HGM11327 | *SIG351* | *SIG351 sp017935525* | GCA_017935525.1 | | 97.43 |
| grpDM.bin.174 | d__Bacteria;p__Bacteroidota;c__Bacteroidia;o__Bacteroidales;f__Barnesiellaceae;g__Barnesiella;s__Barnesiella sp937891845 | Bacteria | Bacteroidota | Bacteroidia | Bacteroidales | Barnesiellaceae | *Barnesiella* | *Barnesiella sp937891845* | GCA_937891845.1 | | 98.77 |
| grpDM.bin.180 | d__Bacteria;p__Bacillota;c__Clostridia;o__Oscillospirales;f__CAG-272;g__UBA11512;s__UBA11512 sp003522145 | Bacteria | Bacillota | Clostridia | Oscillospirales | CAG-272 | *UBA11512* | *UBA11512 sp003522145* | GCA_003522145.1 | | 97.28 |
| grpDM.bin.20 | d__Bacteria;p__Bacillota;c__Clostridia;o__Oscillospirales;f__Ruminococcaceae;g__UMGS363;s__ | Bacteria | Bacillota | Clostridia | Oscillospirales | Ruminococcaceae | *UMGS363* | | N/A |  | N/A |
| grpDM.bin.200 | d__Bacteria;p__Bacillota;c__Clostridia;o__Lachnospirales;f__Lachnospiraceae;g__Acetatifactor;s__Acetatifactor sp016303085 | Bacteria | Bacillota | Clostridia | Lachnospirales | Lachnospiraceae | *Acetatifactor* | *Acetatifactor sp016303085* | GCA_016303085.1 | | 98.82 |
| grpDM.bin.201 | d__Bacteria;p__Bacillota;c__Clostridia;o__Peptostreptococcales;f__Anaerovoracaceae;g__RGIG9096;s__RGIG9096 sp017936025 | Bacteria | Bacillota | Clostridia | Peptostreptococcales | Anaerovoracaceae | *RGIG9096* | *RGIG9096 sp017936025* | GCA_017936025.1 | | 98.39 |
| grpDM.bin.202 | d__Bacteria;p__Bacillota;c__Clostridia;o__Christensenellales;f__CAG-314;g__SFEB01;s__SFEB01 sp004558105 | Bacteria | Bacillota | Clostridia | Christensenellales | CAG-314 | *SFEB01* | *SFEB01 sp004558105* | GCA_004558105.1 | | 97.94 |
| grpDM.bin.203 | d__Bacteria;p__Bacillota;c__Clostridia;o__Peptostreptococcales;f__Anaerovoracaceae;g__RGIG446;s__RGIG446 sp017621195 | Bacteria | Bacillota | Clostridia | Peptostreptococcales | Anaerovoracaceae | *RGIG446* | *RGIG446 sp017621195* | GCA_017621195.1 | | 99.41 |
| grpDM.bin.206 | d__Bacteria;p__Bacillota;c__Clostridia;o__Oscillospirales;f__Ruminococcaceae;g__Ruminococcus;s__Ruminococcus sp017441505 | Bacteria | Bacillota | Clostridia | Oscillospirales | Ruminococcaceae | *Ruminococcus* | *Ruminococcus sp017441505* | GCA_017441505.1 | | 98.4 |
| grpDM.bin.207 | d__Bacteria;p__Bacillota;c__Clostridia;o__Oscillospirales;f__CAG-272;g__UMGS1865;s__ | Bacteria | Bacillota | Clostridia | Oscillospirales | CAG-272 | *UMGS1865* | | N/A |  | N/A |
| grpDM.bin.215 | d__Bacteria;p__Bacillota;c__Clostridia;o__Lachnospirales;f__Lachnospiraceae;g__RGIG4057;s__RGIG4057 sp945836355 | Bacteria | Bacillota | Clostridia | Lachnospirales | Lachnospiraceae | *RGIG4057* | *RGIG4057 sp945836355* | GCA_945836355.1 | | 95.47 |
| grpDM.bin.216 | d__Bacteria;p__Bacillota;c__Clostridia;o__Oscillospirales;f__Oscillospiraceae;g__Apopatocola;s__Apopatocola sp029009095 | Bacteria | Bacillota | Clostridia | Oscillospirales | Oscillospiraceae | *Apopatocola* | *Apopatocola sp029009095* | GCA_029009095.1 | | 98.86 |
| grpDM.bin.218 | d__Bacteria;p__Bacillota;c__Clostridia;o__Lachnospirales;f__Lachnospiraceae;g__CALZKI01;s__CALZKI01 sp945787995 | Bacteria | Bacillota | Clostridia | Lachnospirales | Lachnospiraceae | *CALZKI01* | *CALZKI01 sp945787995* | GCA_945787995.1 | | 98.74 |
| grpDM.bin.22 | d__Bacteria;p__Bacillota;c__Clostridia;o__RUG12999;f__RUG12999;g__RUG12999;s__ | Bacteria | Bacillota | Clostridia | RUG12999 | RUG12999 | *RUG12999* | | N/A |  | N/A |
| grpDM.bin.222 | d__Bacteria;p__Bacillota;c__Clostridia;o__Oscillospirales;f__Acutalibacteraceae;g__RUG592;s__ | Bacteria | Bacillota | Clostridia | Oscillospirales | Acutalibacteraceae | *RUG592* |  | N/A |  | N/A |
| grpDM.bin.225 | d__Bacteria;p__Bacillota;c__Clostridia;o__Oscillospirales;f__CAG-272;g__RGIG2000;s__RGIG2000 sp017625915 | Bacteria | Bacillota | Clostridia | Oscillospirales | CAG-272 | *RGIG2000* | *RGIG2000 sp017625915* | GCA_017625915.1 | | 98.72 |
| grpDM.bin.226 | d__Bacteria;p__Bacillota;c__Clostridia;o__Christensenellales;f__CAG-917;g__CAG-1138;s__CAG-1138 sp000434675 | Bacteria | Bacillota | Clostridia | Christensenellales | CAG-917 | *CAG-1138* | *CAG-1138 sp000434675* | GCA_000434675.1 | | 98.34 |
| grpDM.bin.23 | d__Bacteria;p__Pseudomonadota;c__Gammaproteobacteria;o__Enterobacterales;f__Succinivibrionaceae;g__Succinivibrio;s__Succinivibrio sp003456415 | Bacteria | Pseudomonadota | Gammaproteobacteria | Enterobacterales | Succinivibrionaceae | *Succinivibrio* | *Succinivibrio sp003456415* | GCA_902785325.1 | | 98.71 |
| grpDM.bin.230 | d__Bacteria;p__Bacillota;c__Clostridia;o__Oscillospirales;f__Acutalibacteraceae;g__DTU089;s__ | Bacteria | Bacillota | Clostridia | Oscillospirales | Acutalibacteraceae | *DTU089* |  | N/A |  | N/A |
| grpDM.bin.232 | d__Bacteria;p__Bacillota;c__Bacilli;o__Acholeplasmatales;f__Anaeroplasmataceae;g__Anaeroplasma;s__Anaeroplasma sp017413585 | Bacteria | Bacillota | Bacilli | Acholeplasmatales | Anaeroplasmataceae | *Anaeroplasma* | *Anaeroplasma sp017413585* | GCA_017413585.1 | | 97.85 |
| grpDM.bin.233 | d__Bacteria;p__Bacillota;c__Clostridia;o__Oscillospirales;f__Ruminococcaceae;g__RGIG8773;s__RGIG8773 sp017847885 | Bacteria | Bacillota | Clostridia | Oscillospirales | Ruminococcaceae | *RGIG8773* | *RGIG8773 sp017847885* | GCA_017847885.1 | | 97.92 |
| grpDM.bin.236 | d__Bacteria;p__Bacillota;c__Clostridia;o__Lachnospirales;f__Lachnospiraceae;g__Eubacterium_Q;s__Eubacterium_Q sp002349225 | Bacteria | Bacillota | Clostridia | Lachnospirales | Lachnospiraceae | *Eubacterium_Q* | *Eubacterium_Q sp002349225* | GCA_902760415.1 | | 98.16 |
| grpDM.bin.244 | d__Bacteria;p__Bacillota;c__Clostridia;o__Lachnospirales;f__Lachnospiraceae;g__CAMAHO01;s__CAMAHO01 sp945834545 | Bacteria | Bacillota | Clostridia | Lachnospirales | Lachnospiraceae | *CAMAHO01* | *CAMAHO01 sp945834545* | GCA_945834545.1 | | 98.16 |
| grpDM.bin.253 | d__Bacteria;p__Bacillota;c__Clostridia;o__Christensenellales;f__Borkfalkiaceae;g__Scatosoma;s__ | Bacteria | Bacillota | Clostridia | Christensenellales | Borkfalkiaceae | *Scatosoma* | | N/A |  | N/A |
| grpDM.bin.256 | d__Bacteria;p__Elusimicrobiota;c__Elusimicrobia;o__Elusimicrobiales;f__Elusimicrobiaceae;g__Avelusimicrobium;s__Avelusimicrobium sp900762815 | Bacteria | Elusimicrobiota | Elusimicrobia | Elusimicrobiales | Elusimicrobiaceae | *Avelusimicrobium* | *Avelusimicrobium sp900762815* | GCA_900762815.1 | | 98.78 |
| grpDM.bin.258 | d__Bacteria;p__Bacillota;c__Clostridia;o__Oscillospirales;f__Ruminococcaceae;g__UMGS1668;s__UMGS1668 sp017468365 | Bacteria | Bacillota | Clostridia | Oscillospirales | Ruminococcaceae | *UMGS1668* | *UMGS1668 sp017468365* | GCA_017468365.1 | | 98.5 |
| grpDM.bin.261 | d__Bacteria;p__Bacteroidota;c__Bacteroidia;o__Bacteroidales;f__Bacteroidaceae;g__Prevotella;s__Prevotella sp900315545 | Bacteria | Bacteroidota | Bacteroidia | Bacteroidales | Bacteroidaceae | *Prevotella* | *Prevotella sp900315545* | GCA_900315545.1 | | 98.58 |
| grpDM.bin.264 | d__Bacteria;p__Bacillota;c__Clostridia;o__Peptostreptococcales;f__Anaerovoracaceae;g__Fimisoma;s__Fimisoma sp002320005 | Bacteria | Bacillota | Clostridia | Peptostreptococcales | Anaerovoracaceae | *Fimisoma* | *Fimisoma sp002320005* | GCA_002320005.1 | | 98 |
| grpDM.bin.269 | d__Bacteria;p__Spirochaetota;c__Spirochaetia;o__Treponematales;f__Treponemataceae;g__Treponema_D;s__Treponema_D berlinense | Bacteria | Spirochaetota | Spirochaetia | Treponematales | Treponemataceae | *Treponema_D* | *Treponema_D berlinense* | GCF_900167025.1 | | 98.88 |
| grpDM.bin.27 | d__Bacteria;p__Bacillota;c__Clostridia;o__Oscillospirales;f__Acutalibacteraceae;g__UBA8612;s__ | Bacteria | Bacillota | Clostridia | Oscillospirales | Acutalibacteraceae | *UBA8612* | | N/A |  | N/A |
| grpDM.bin.274 | d__Bacteria;p__Bacillota;c__Clostridia;o__Oscillospirales;f__Acutalibacteraceae;g__DTU089;s__ | Bacteria | Bacillota | Clostridia | Oscillospirales | Acutalibacteraceae | *DTU089* |  | N/A |  | N/A |
| grpDM.bin.283 | d__Bacteria;p__Bacillota;c__Clostridia;o__Lachnospirales;f__Lachnospiraceae;g__Pilosibacter;s__ | Bacteria | Bacillota | Clostridia | Lachnospirales | Lachnospiraceae | *Pilosibacter* | | N/A |  | N/A |
| grpDM.bin.284 | d__Bacteria;p__Bacteroidota;c__Bacteroidia;o__Bacteroidales;f__Rikenellaceae;g__Alistipes;s__Alistipes senegalensis | Bacteria | Bacteroidota | Bacteroidia | Bacteroidales | Rikenellaceae | *Alistipes* | *Alistipes senegalensis* | GCF_025145645.1 | | 98.18 |
| grpDM.bin.285 | d__Bacteria;p__Bacillota;c__Clostridia;o__Lachnospirales;f__Lachnospiraceae;g__SIG307;s__ | Bacteria | Bacillota | Clostridia | Lachnospirales | Lachnospiraceae | *SIG307* |  | N/A |  | N/A |
| grpDM.bin.29 | d__Bacteria;p__Bacillota;c__Clostridia;o__Oscillospirales;f__Acutalibacteraceae;g__Ruminococcoides;s__ | Bacteria | Bacillota | Clostridia | Oscillospirales | Acutalibacteraceae | *Ruminococcoides* | | N/A |  | N/A |
| grpDM.bin.294 | d__Bacteria;p__Bacillota;c__Bacilli;o__ML615J-28;f__CAG-313;g__UMGS75;s__ | Bacteria | Bacillota | Bacilli | ML615J-28 | CAG-313 | *UMGS75* |  | N/A |  | N/A |
| grpDM.bin.295 | d__Bacteria;p__Bacillota;c__Clostridia;o__Lachnospirales;f__Lachnospiraceae;g__Acetatifactor;s__Acetatifactor sp017621075 | Bacteria | Bacillota | Clostridia | Lachnospirales | Lachnospiraceae | *Acetatifactor* | *Acetatifactor sp017621075* | GCA_017621075.1 | | 99.64 |
| grpDM.bin.296 | d__Bacteria;p__Bacteroidota;c__Bacteroidia;o__Bacteroidales;f__Barnesiellaceae;g__Barnesiella;s__Barnesiella sp017410245 | Bacteria | Bacteroidota | Bacteroidia | Bacteroidales | Barnesiellaceae | *Barnesiella* | *Barnesiella sp017410245* | GCA_017410245.1 | | 98.76 |
| grpDM.bin.301 | d__Bacteria;p__Bacillota;c__Clostridia;o__Oscillospirales;f__Acutalibacteraceae;g__;s__ | Bacteria | Bacillota | Clostridia | Oscillospirales | *Acutalibacteraceae* | |  | N/A |  | N/A |
| grpDM.bin.308 | d__Bacteria;p__Bacillota;c__Clostridia;o__Christensenellales;f__Pumilibacteraceae;g__RUG12826;s__ | Bacteria | Bacillota | Clostridia | Christensenellales | Pumilibacteraceae | *RUG12826* | | N/A |  | N/A |
| grpDM.bin.31 | d__Bacteria;p__Bacillota;c__Clostridia;o__Christensenellales;f__UBA1242;g__UMGS687;s__UMGS687 sp900544595 | Bacteria | Bacillota | Clostridia | Christensenellales | UBA1242 | *UMGS687* | *UMGS687 sp900544595* | GCA_900544595.1 | | 98.48 |
| grpDM.bin.310 | d__Bacteria;p__Bacteroidota;c__Bacteroidia;o__Bacteroidales;f__Rikenellaceae;g__Alistipes;s__Alistipes sp017465055 | Bacteria | Bacteroidota | Bacteroidia | Bacteroidales | Rikenellaceae | *Alistipes* | *Alistipes sp017465055* | GCA_017465055.1 | | 97.13 |
| grpDM.bin.314 | d__Bacteria;p__Bacteroidota;c__Bacteroidia;o__Bacteroidales;f__UBA932;g__Cryptobacteroides;s__Cryptobacteroides sp900316045 | Bacteria | Bacteroidota | Bacteroidia | Bacteroidales | UBA932 | *Cryptobacteroides* | *Cryptobacteroides sp900316045* | GCA_900316045.1 | | 98.78 |
| grpDM.bin.318 | d__Bacteria;p__Bacillota;c__Clostridia;o__Lachnospirales;f__Lachnospiraceae;g__Butyrivibrio_A;s__ | Bacteria | Bacillota | Clostridia | Lachnospirales | Lachnospiraceae | *Butyrivibrio_A* | | N/A |  | N/A |
| grpDM.bin.32 | d__Bacteria;p__Bacillota;c__Clostridia;o__Oscillospirales;f__Ruminococcaceae;g__HUN007;s__HUN007 sp945876405 | Bacteria | Bacillota | Clostridia | Oscillospirales | Ruminococcaceae | *HUN007* | *HUN007 sp945876405* | GCA_945876405.1 | | 98.25 |
| grpDM.bin.33 | d__Bacteria;p__Bacillota;c__Clostridia;o__Christensenellales;f__Aristaeellaceae;g__SFMI01;s__SFMI01 sp004556155 | Bacteria | Bacillota | Clostridia | Christensenellales | Aristaeellaceae | *SFMI01* | *SFMI01 sp004556155* | GCA_004556155.1 | | 98.76 |
| grpDM.bin.34 | d__Bacteria;p__Bacillota;c__Clostridia;o__Christensenellales;f__Pumilibacteraceae;g__UBA10677;s__UBA10677 sp017623435 | Bacteria | Bacillota | Clostridia | Christensenellales | Pumilibacteraceae | *UBA10677* | *UBA10677 sp017623435* | GCA_017623435.1 | | 97.81 |
| grpDM.bin.37 | d__Bacteria;p__Bacillota;c__Clostridia;o__Lachnospirales;f__Anaerotignaceae;g__Anaerotignum_A;s__Anaerotignum_A sp945912105 | Bacteria | Bacillota | Clostridia | Lachnospirales | Anaerotignaceae | *Anaerotignum_A* | *Anaerotignum_A sp945912105* | GCA_945912105.1 | | 98.25 |
| grpDM.bin.38 | d__Bacteria;p__Bacillota;c__Clostridia;o__Oscillospirales;f__CAG-272;g__CAG-448;s__ | Bacteria | Bacillota | Clostridia | Oscillospirales | CAG-272 | *CAG-448* |  | N/A |  | N/A |
| grpDM.bin.39 | d__Bacteria;p__Cyanobacteriota;c__Vampirovibrionia;o__Gastranaerophilales;f__Gastranaerophilaceae;g__CAG-196;s__ | Bacteria | Cyanobacteriota | Vampirovibrionia | Gastranaerophilales | Gastranaerophilaceae | *CAG-196* |  | N/A |  | N/A |
| grpDM.bin.4 | d__Bacteria;p__Bacteroidota;c__Bacteroidia;o__Bacteroidales;f__Muribaculaceae;g__Sodaliphilus;s__Sodaliphilus sp004557565 | Bacteria | Bacteroidota | Bacteroidia | Bacteroidales | Muribaculaceae | *Sodaliphilus* | *Sodaliphilus sp004557565* | GCA_004557565.1 | | 97.96 |
| grpDM.bin.42 | d__Bacteria;p__Bacteroidota;c__Bacteroidia;o__Bacteroidales;f__Rikenellaceae;g__Alistipes;s__Alistipes sp017399485 | Bacteria | Bacteroidota | Bacteroidia | Bacteroidales | Rikenellaceae | *Alistipes* | *Alistipes sp017399485* | GCA_017399485.1 | | 96.04 |
| grpDM.bin.44 | d__Bacteria;p__Bacillota;c__Clostridia;o__Oscillospirales;f__Acutalibacteraceae;g__UBA737;s__UBA737 sp902762875 | Bacteria | Bacillota | Clostridia | Oscillospirales | Acutalibacteraceae | *UBA737* | *UBA737 sp902762875* | GCA_902762875.1 | | 95.65 |
| grpDM.bin.48 | d__Bacteria;p__Bacillota;c__Clostridia;o__Oscillospirales;f__CAG-272;g__UBA1740;s__UBA1740 sp017938425 | Bacteria | Bacillota | Clostridia | Oscillospirales | CAG-272 | *UBA1740* | *UBA1740 sp017938425* | GCA_017938425.1 | | 97.8 |
| grpDM.bin.5 | d__Bacteria;p__Bacillota;c__Clostridia;o__Lachnospirales;f__Lachnospiraceae;g__JALENY01;s__ | Bacteria | Bacillota | Clostridia | Lachnospirales | Lachnospiraceae | *JALENY01* | | N/A |  | N/A |
| grpDM.bin.52 | d__Bacteria;p__Bacillota;c__Clostridia;o__Christensenellales;f__Borkfalkiaceae;g__Scatosoma;s__ | Bacteria | Bacillota | Clostridia | Christensenellales | Borkfalkiaceae | *Scatosoma* | | N/A |  | N/A |
| grpDM.bin.6 | d__Bacteria;p__Elusimicrobiota;c__Elusimicrobia;o__Elusimicrobiales;f__Elusimicrobiaceae;g__Avelusimicrobium;s__Avelusimicrobium sp002329395 | Bacteria | Elusimicrobiota | Elusimicrobia | Elusimicrobiales | Elusimicrobiaceae | *Avelusimicrobium* | *Avelusimicrobium sp002329395* | GCA_002329395.1 | | 98.71 |
| grpDM.bin.64 | d__Bacteria;p__Bacillota;c__Clostridia;o__Christensenellales;f__CAG-314;g__Heteroclostridium;s__ | Bacteria | Bacillota | Clostridia | Christensenellales | CAG-314 | *Heteroclostridium* | | N/A |  | N/A |
| grpDM.bin.67 | d__Bacteria;p__Bacillota;c__Clostridia;o__Oscillospirales;f__Acutalibacteraceae;g__RGIG3009;s__RGIG3009 sp017503785 | Bacteria | Bacillota | Clostridia | Oscillospirales | Acutalibacteraceae | *RGIG3009* | *RGIG3009 sp017503785* | GCA_017503785.1 | | 98.52 |
| grpDM.bin.7 | d__Bacteria;p__Pseudomonadota;c__Alphaproteobacteria;o__RF32;f__CAG-239;g__;s__ | Bacteria | Pseudomonadota | Alphaproteobacteria | RF32 | CAG-239 |  |  | N/A |  | N/A |
| grpDM.bin.71 | d__Bacteria;p__Bacillota;c__Clostridia;o__Lachnospirales;f__Lachnospiraceae;g__RGIG1668;s__ | Bacteria | Bacillota | Clostridia | Lachnospirales | Lachnospiraceae | *RGIG1668* | | N/A |  | N/A |
| grpDM.bin.72 | d__Bacteria;p__Bacillota;c__Clostridia;o__Oscillospirales;f__Ruminococcaceae;g__Ruminococcus;s__Ruminococcus sp017534835 | Bacteria | Bacillota | Clostridia | Oscillospirales | Ruminococcaceae | *Ruminococcus* | *Ruminococcus sp017534835* | GCA_017534835.1 | | 97.91 |
| grpDM.bin.87 | d__Bacteria;p__Bacillota;c__Clostridia;o__Oscillospirales;f__Acutalibacteraceae;g__Ruminococcoides;s__Ruminococcoides sp029007475 | Bacteria | Bacillota | Clostridia | Oscillospirales | Acutalibacteraceae | *Ruminococcoides* | *Ruminococcoides sp029007475* | GCA_029007475.1 | | 97.82 |
| grpDM.bin.89 | d__Bacteria;p__Elusimicrobiota;c__Elusimicrobia;o__Elusimicrobiales;f__Elusimicrobiaceae;g__UBA1174;s__UBA1174 sp900556855 | Bacteria | Elusimicrobiota | Elusimicrobia | Elusimicrobiales | Elusimicrobiaceae | *UBA1174* | *UBA1174 sp900556855* | GCA_934643455.1 | | 97.34 |
| grpDM.bin.92 | d__Bacteria;p__Bacillota;c__Clostridia;o__Oscillospirales;f__CAG-272;g__HGM12713;s__HGM12713 sp017504545 | Bacteria | Bacillota | Clostridia | Oscillospirales | CAG-272 | *HGM12713* | *HGM12713 sp017504545* | GCA_017504545.1 | | 97.35 |
| grpDM.bin.93 | d__Bacteria;p__Bacillota;c__Clostridia;o__Lachnospirales;f__Lachnospiraceae;g__SFDP01;s__ | Bacteria | Bacillota | Clostridia | Lachnospirales | Lachnospiraceae | *SFDP01* |  | N/A |  | N/A |
| grpDM.bin.94 | d__Bacteria;p__Spirochaetota;c__Spirochaetia;o__Treponematales;f__Treponemataceae;g__Treponema_D;s__Treponema_D sp002373205 | Bacteria | Spirochaetota | Spirochaetia | Treponematales | Treponemataceae | *Treponema_D* | *Treponema_D sp002373205* | GCA_002373205.1 | | 96.69 |
| grpDM.bin.96 | d__Bacteria;p__Verrucomicrobiota;c__Lentisphaeria;o__Victivallales;f__UBA1829;g__UBA11452;s__UBA11452 sp003526375 | Bacteria | Verrucomicrobiota | Lentisphaeria | Victivallales | UBA1829 | *UBA11452* | *UBA11452 sp003526375* | GCA_003526375.1 | | 98.92 |
| grpH.bin.10 | d__Bacteria;p__Pseudomonadota;c__Alphaproteobacteria;o__RF32;f__CAG-239;g__RUG410;s__RUG410 sp030523245 | Bacteria | Pseudomonadota | Alphaproteobacteria | RF32 | CAG-239 | *RUG410* | *RUG410 sp030523245* | GCA_030523245.1 | | 99.21 |
| grpH.bin.100 | d__Bacteria;p__Bacteroidota;c__Bacteroidia;o__Bacteroidales;f__Muribaculaceae;g__UBA7173;s__UBA7173 sp945931475 | Bacteria | Bacteroidota | Bacteroidia | Bacteroidales | Muribaculaceae | *UBA7173* | *UBA7173 sp945931475* | GCA_945931475.1 | | 99.09 |
| grpH.bin.102 | d__Bacteria;p__Bacillota;c__Clostridia;o__Oscillospirales;f__CAG-272;g__UMGS1002;s__UMGS1002 sp900547565 | Bacteria | Bacillota | Clostridia | Oscillospirales | CAG-272 | *UMGS1002* | *UMGS1002 sp900547565* | GCA_900547565.1 | | 97.37 |
| grpH.bin.122 | d__Bacteria;p__Bacteroidota;c__Bacteroidia;o__Bacteroidales;f__Bacteroidaceae;g__Prevotella;s__Prevotella sp900547005 | Bacteria | Bacteroidota | Bacteroidia | Bacteroidales | Bacteroidaceae | *Prevotella* | *Prevotella sp900547005* | GCA_900547005.1 | | 96.32 |
| grpH.bin.126 | d__Bacteria;p__Bacillota;c__Clostridia;o__Oscillospirales;f__Acutalibacteraceae;g__UBA6857;s__ | Bacteria | Bacillota | Clostridia | Oscillospirales | Acutalibacteraceae | *UBA6857* | | N/A |  | N/A |
| grpH.bin.129 | d__Bacteria;p__Bacteroidota;c__Bacteroidia;o__Bacteroidales;f__Muribaculaceae;g__CAG-873;s__ | Bacteria | Bacteroidota | Bacteroidia | Bacteroidales | Muribaculaceae | *CAG-873* |  | N/A |  | N/A |
| grpH.bin.132 | d__Bacteria;p__Bacillota;c__Clostridia;o__Lachnospirales;f__Lachnospiraceae;g__Lachnospira;s__Lachnospira sp017626275 | Bacteria | Bacillota | Clostridia | Lachnospirales | Lachnospiraceae | *Lachnospira* | *Lachnospira sp017626275* | GCA_017626275.1 | | 98.32 |
| grpH.bin.135 | d__Bacteria;p__Bacteroidota;c__Bacteroidia;o__Bacteroidales;f__Muribaculaceae;g__Lepagella;s__Lepagella sp945873625 | Bacteria | Bacteroidota | Bacteroidia | Bacteroidales | Muribaculaceae | *Lepagella* | *Lepagella sp945873625* | GCA_945873625.1 | | 99.13 |
| grpH.bin.136 | d__Bacteria;p__Pseudomonadota;c__Gammaproteobacteria;o__Enterobacterales;f__Succinivibrionaceae;g__HGM20899;s__HGM20899 sp900767005 | Bacteria | Pseudomonadota | Gammaproteobacteria | Enterobacterales | Succinivibrionaceae | *HGM20899* | *HGM20899 sp900767005* | GCA_900767005.1 | | 99.48 |
| grpH.bin.143 | d__Bacteria;p__Bacteroidota;c__Bacteroidia;o__Bacteroidales;f__Muribaculaceae;g__Duncaniella;s__Duncaniella sp019416285 | Bacteria | Bacteroidota | Bacteroidia | Bacteroidales | Muribaculaceae | *Duncaniella* | *Duncaniella sp019416285* | GCA_019416285.1 | | 96.92 |
| grpH.bin.152 | d__Bacteria;p__Bacillota;c__Clostridia;o__UMGS1840;f__UMGS1840;g__SIG452;s__SIG452 sp017468465 | Bacteria | Bacillota | Clostridia | UMGS1840 | UMGS1840 | *SIG452* | *SIG452 sp017468465* | GCA_017468465.1 | | 98.01 |
| grpH.bin.156 | d__Bacteria;p__Bacteroidota;c__Bacteroidia;o__Bacteroidales;f__P3;g__Colimorpha;s__Colimorpha sp017522105 | Bacteria | Bacteroidota | Bacteroidia | Bacteroidales | P3 | *Colimorpha* | *Colimorpha sp017522105* | GCA_017522105.1 | | 96.81 |
| grpH.bin.160 | d__Bacteria;p__Bacillota;c__Clostridia;o__Lachnospirales;f__Lachnospiraceae;g__Wujia;s__Wujia sp945492935 | Bacteria | Bacillota | Clostridia | Lachnospirales | Lachnospiraceae | *Wujia* | *Wujia sp945492935* | GCA_945492935.1 | | 98.91 |
| grpH.bin.171 | d__Bacteria;p__Bacillota;c__Clostridia;o__Oscillospirales;f__Acutalibacteraceae;g__RUG420;s__RUG420 sp900317085 | Bacteria | Bacillota | Clostridia | Oscillospirales | Acutalibacteraceae | *RUG420* | *RUG420 sp900317085* | GCA_900317085.1 | | 98.9 |
| grpH.bin.175 | d__Bacteria;p__Bacillota;c__Bacilli;o__RFN20;f__CAG-826;g__Onthovivens;s__Onthovivens sp016302065 | Bacteria | Bacillota | Bacilli | RFN20 | CAG-826 | *Onthovivens* | *Onthovivens sp016302065* | GCA_016302065.1 | | 98.05 |
| grpH.bin.18 | d__Bacteria;p__Bacteroidota;c__Bacteroidia;o__Bacteroidales;f__UBA932;g__Cryptobacteroides;s__Cryptobacteroides sp900546925 | Bacteria | Bacteroidota | Bacteroidia | Bacteroidales | UBA932 | *Cryptobacteroides* | *Cryptobacteroides sp900546925* | GCA_900546925.1 | | 98.48 |
| grpH.bin.181 | d__Bacteria;p__Bacillota;c__Clostridia;o__Oscillospirales;f__CAG-272;g__RGIG2000;s__ | Bacteria | Bacillota | Clostridia | Oscillospirales | CAG-272 | *RGIG2000* | | N/A |  | N/A |
| grpH.bin.185 | d__Bacteria;p__Bacillota;c__Bacilli;o__Erysipelotrichales;f__Coprobacillaceae;g__Faecalibacillus;s__ | Bacteria | Bacillota | Bacilli | Erysipelotrichales | Coprobacillaceae | *Faecalibacillus* | | N/A |  | N/A |
| grpH.bin.190 | d__Bacteria;p__Bacillota;c__Clostridia;o__Oscillospirales;f__Ruminococcaceae;g__Ruminococcus;s__Ruminococcus sp015066935 | Bacteria | Bacillota | Clostridia | Oscillospirales | Ruminococcaceae | *Ruminococcus* | *Ruminococcus sp015066935* | GCA_015066935.1 | | 98.47 |
| grpH.bin.192 | d__Bacteria;p__Bacillota;c__Clostridia;o__Oscillospirales;f__Oscillospiraceae;g__Faecousia;s__ | Bacteria | Bacillota | Clostridia | Oscillospirales | Oscillospiraceae | *Faecousia* |  | N/A |  | N/A |
| grpH.bin.194 | d__Bacteria;p__Bacillota;c__Clostridia;o__Oscillospirales;f__Acutalibacteraceae;g__UBA6857;s__ | Bacteria | Bacillota | Clostridia | Oscillospirales | Acutalibacteraceae | *UBA6857* | | N/A |  | N/A |
| grpH.bin.196 | d__Bacteria;p__Bacteroidota;c__Bacteroidia;o__Bacteroidales;f__Muribaculaceae;g__RGIG4079;s__RGIG4079 sp017399915 | Bacteria | Bacteroidota | Bacteroidia | Bacteroidales | Muribaculaceae | *RGIG4079* | *RGIG4079 sp017399915* | GCA_017399915.1 | | 96.83 |
| grpH.bin.20 | d__Bacteria;p__Bacteroidota;c__Bacteroidia;o__Bacteroidales;f__Bacteroidaceae;g__HGM04593;s__HGM04593 sp017938245 | Bacteria | Bacteroidota | Bacteroidia | Bacteroidales | Bacteroidaceae | *HGM04593* | *HGM04593 sp017938245* | GCA_017938245.1 | | 98.34 |
| grpH.bin.202 | d__Bacteria;p__Bacillota;c__Clostridia;o__Oscillospirales;f__Acutalibacteraceae;g__RGIG3009;s__RGIG3009 sp017503785 | Bacteria | Bacillota | Clostridia | Oscillospirales | Acutalibacteraceae | *RGIG3009* | *RGIG3009 sp017503785* | GCA_017503785.1 | | 98.43 |
| grpH.bin.21 | d__Bacteria;p__Bacillota;c__Clostridia;o__Oscillospirales;f__Oscillospiraceae;g__F23-B02;s__F23-B02 sp016292445 | Bacteria | Bacillota | Clostridia | Oscillospirales | Oscillospiraceae | *F23-B02* | *F23-B02 sp016292445* | GCA_016292445.1 | | 98.52 |
| grpH.bin.210 | d__Bacteria;p__Bacillota;c__Clostridia;o__Oscillospirales;f__CAG-272;g__UBA4717;s__ | Bacteria | Bacillota | Clostridia | Oscillospirales | CAG-272 | *UBA4717* | | N/A |  | N/A |
| grpH.bin.212 | d__Bacteria;p__Bacteroidota;c__Bacteroidia;o__Bacteroidales;f__Bacteroidaceae;g__HGM04593;s__HGM04593 sp017416705 | Bacteria | Bacteroidota | Bacteroidia | Bacteroidales | Bacteroidaceae | *HGM04593* | *HGM04593 sp017416705* | GCA_017416705.1 | | 97.95 |
| grpH.bin.218 | d__Bacteria;p__Bacillota;c__Clostridia;o__Oscillospirales;f__CAG-272;g__RGIG3925;s__ | Bacteria | Bacillota | Clostridia | Oscillospirales | CAG-272 | *RGIG3925* | | N/A |  | N/A |
| grpH.bin.225 | d__Bacteria;p__Bacillota;c__Clostridia;o__Oscillospirales;f__CAG-382;g__UMGS1052;s__ | Bacteria | Bacillota | Clostridia | Oscillospirales | CAG-382 | *UMGS1052* | | N/A |  | N/A |
| grpH.bin.229 | d__Bacteria;p__Pseudomonadota;c__Alphaproteobacteria;o__RF32;f__CAG-239;g__CAZU01;s__ | Bacteria | Pseudomonadota | Alphaproteobacteria | RF32 | CAG-239 | *CAZU01* |  | N/A |  | N/A |
| grpH.bin.23 | d__Bacteria;p__Bacillota;c__Clostridia;o__Oscillospirales;f__Acutalibacteraceae;g__DTU089;s__DTU089 sp017937865 | Bacteria | Bacillota | Clostridia | Oscillospirales | Acutalibacteraceae | *DTU089* | *DTU089 sp017937865* | GCA_017937865.1 | | 97.67 |
| grpH.bin.236 | d__Bacteria;p__Bacillota;c__Clostridia;o__Oscillospirales;f__CAG-272;g__CAG-272;s__ | Bacteria | Bacillota | Clostridia | Oscillospirales | CAG-272 | *CAG-272* |  | N/A |  | N/A |
| grpH.bin.237 | d__Bacteria;p__Bacillota;c__Clostridia;o__Oscillospirales;f__Ruminococcaceae;g__UMGS1668;s__UMGS1668 sp017847875 | Bacteria | Bacillota | Clostridia | Oscillospirales | Ruminococcaceae | *UMGS1668* | *UMGS1668 sp017847875* | GCA_017847875.1 | | 97.92 |
| grpH.bin.238 | d__Bacteria;p__Bacteroidota;c__Bacteroidia;o__Bacteroidales;f__Bacteroidaceae;g__UBA6398;s__UBA6398 sp003150315 | Bacteria | Bacteroidota | Bacteroidia | Bacteroidales | Bacteroidaceae | *UBA6398* | *UBA6398 sp003150315* | GCA_028724165.1 | | 99.41 |
| grpH.bin.243 | d__Bacteria;p__Bacteroidota;c__Bacteroidia;o__Bacteroidales;f__Bacteroidaceae;g__Prevotella;s__Prevotella sp002351725 | Bacteria | Bacteroidota | Bacteroidia | Bacteroidales | Bacteroidaceae | *Prevotella* | *Prevotella sp002351725* | GCA_002351725.1 | | 96.79 |
| grpH.bin.245 | d__Bacteria;p__Bacillota;c__Clostridia;o__Christensenellales;f__Borkfalkiaceae;g__UBA10281;s__UBA10281 sp945835865 | Bacteria | Bacillota | Clostridia | Christensenellales | Borkfalkiaceae | *UBA10281* | *UBA10281 sp945835865* | GCA_945835865.1 | | 98.92 |
| grpH.bin.249 | d__Bacteria;p__Bacillota;c__Clostridia;o__Oscillospirales;f__CAG-272;g__UMGS1696;s__ | Bacteria | Bacillota | Clostridia | Oscillospirales | CAG-272 | *UMGS1696* | | N/A |  | N/A |
| grpH.bin.256 | d__Bacteria;p__Bacillota;c__Clostridia;o__Lachnospirales;f__Lachnospiraceae;g__Gallintestinimicrobium;s__ | Bacteria | Bacillota | Clostridia | Lachnospirales | Lachnospiraceae | *Gallintestinimicrobium* | | N/A |  | N/A |
| grpH.bin.266 | d__Bacteria;p__Bacillota;c__Clostridia;o__Oscillospirales;f__CAG-272;g__RUG12395;s__RUG12395 sp015064505 | Bacteria | Bacillota | Clostridia | Oscillospirales | CAG-272 | *RUG12395* | *RUG12395 sp015064505* | GCA_015064505.1 | | 97.82 |
| grpH.bin.271 | d__Bacteria;p__Bacteroidota;c__Bacteroidia;o__Bacteroidales;f__UBA932;g__Cryptobacteroides;s__ | Bacteria | Bacteroidota | Bacteroidia | Bacteroidales | UBA932 | *Cryptobacteroides* | | N/A |  | N/A |
| grpH.bin.275 | d__Bacteria;p__Bacteroidota;c__Bacteroidia;o__Bacteroidales;f__Muribaculaceae;g__Lepagella;s__Lepagella sp000437495 | Bacteria | Bacteroidota | Bacteroidia | Bacteroidales | Muribaculaceae | *Lepagella* | *Lepagella sp000437495* | GCA_000437495.1 | | 99.47 |
| grpH.bin.276 | d__Bacteria;p__Bacillota;c__Clostridia;o__Oscillospirales;f__Ruminococcaceae;g__Ruminococcus;s__Ruminococcus flavefaciens_V | Bacteria | Bacillota | Clostridia | Oscillospirales | Ruminococcaceae | *Ruminococcus* | *Ruminococcus flavefaciens_V* | GCA_902762855.1 | | 98.48 |
| grpH.bin.28 | d__Bacteria;p__Cyanobacteriota;c__Vampirovibrionia;o__Gastranaerophilales;f__Gastranaerophilaceae;g__Zag111;s__Zag111 sp017626055 | Bacteria | Cyanobacteriota | Vampirovibrionia | Gastranaerophilales | Gastranaerophilaceae | *Zag111* | *Zag111 sp017626055* | GCA_017626055.1 | | 98.31 |
| grpH.bin.281 | d__Bacteria;p__Bacillota;c__Clostridia;o__UMGS1810;f__UMGS1810;g__Qingrenia;s__Qingrenia sp022769845 | Bacteria | Bacillota | Clostridia | UMGS1810 | UMGS1810 | *Qingrenia* | *Qingrenia sp022769845* | GCA_022769845.1 | | 98.58 |
| grpH.bin.288 | d__Bacteria;p__Fibrobacterota;c__Fibrobacteria;o__Fibrobacterales;f__Fibrobacteraceae;g__Hallerella;s__Hallerella sp002390045 | Bacteria | Fibrobacterota | Fibrobacteria | Fibrobacterales | Fibrobacteraceae | *Hallerella* | *Hallerella sp002390045* | GCA_002390045.1 | | 98.46 |
| grpH.bin.294 | d__Bacteria;p__Bacillota;c__Bacilli;o__RF39;f__UBA660;g__CAKPTA01;s__ | Bacteria | Bacillota | Bacilli | RF39 | UBA660 | *CAKPTA01* | | N/A |  | N/A |
| grpH.bin.298 | d__Bacteria;p__Bacillota;c__Clostridia;o__Lachnospirales;f__Lachnospiraceae;g__Butyrivibrio_A;s__ | Bacteria | Bacillota | Clostridia | Lachnospirales | Lachnospiraceae | *Butyrivibrio_A* | | N/A |  | N/A |
| grpH.bin.302 | d__Bacteria;p__Bacteroidota;c__Bacteroidia;o__Bacteroidales;f__Muribaculaceae;g__Lepagella;s__ | Bacteria | Bacteroidota | Bacteroidia | Bacteroidales | Muribaculaceae | *Lepagella* |  | N/A |  | N/A |
| grpH.bin.31 | d__Bacteria;p__Bacteroidota;c__Bacteroidia;o__Bacteroidales;f__Bacteroidaceae;g__Colenecus;s__Colenecus sp900318995 | Bacteria | Bacteroidota | Bacteroidia | Bacteroidales | Bacteroidaceae | *Colenecus* | *Colenecus sp900318995* | GCA_900318995.1 | | 95.93 |
| grpH.bin.310 | d__Bacteria;p__Bacillota;c__Clostridia;o__Oscillospirales;f__CAG-272;g__HGM12713;s__ | Bacteria | Bacillota | Clostridia | Oscillospirales | CAG-272 | *HGM12713* | | N/A |  | N/A |
| grpH.bin.319 | d__Bacteria;p__Bacillota;c__Clostridia;o__Oscillospirales;f__CAG-272;g__HGM12713;s__ | Bacteria | Bacillota | Clostridia | Oscillospirales | CAG-272 | *HGM12713* | | N/A |  | N/A |
| grpH.bin.32 | d__Bacteria;p__Bacillota;c__Clostridia;o__Oscillospirales;f__Ruminococcaceae;g__Ruminococcus;s__Ruminococcus sp017935865 | Bacteria | Bacillota | Clostridia | Oscillospirales | Ruminococcaceae | *Ruminococcus* | *Ruminococcus sp017935865* | GCA_017935865.1 | | 99.79 |
| grpH.bin.323 | d__Bacteria;p__Verrucomicrobiota;c__Verrucomicrobiia;o__Verrucomicrobiales;f__Akkermansiaceae;g__Akkermansia;s__Akkermansia muciniphila | Bacteria | Verrucomicrobiota | Verrucomicrobiia | Verrucomicrobiales | Akkermansiaceae | *Akkermansia* | *Akkermansia muciniphila* | GCF_000020225.1 | | 98.59 |
| grpH.bin.324 | d__Bacteria;p__Bacillota;c__Clostridia;o__Lachnospirales;f__Lachnospiraceae;g__Bovifimicola;s__ | Bacteria | Bacillota | Clostridia | Lachnospirales | Lachnospiraceae | *Bovifimicola* | | N/A |  | N/A |
| grpH.bin.36 | d__Bacteria;p__Bacteroidota;c__Bacteroidia;o__Bacteroidales;f__Muribaculaceae;g__CAG-1031;s__CAG-1031 sp000431215 | Bacteria | Bacteroidota | Bacteroidia | Bacteroidales | Muribaculaceae | *CAG-1031* | *CAG-1031 sp000431215* | GCA_000431215.1 | | 98.6 |
| grpH.bin.39 | d__Bacteria;p__Bacillota;c__Clostridia;o__Oscillospirales;f__Butyricicoccaceae;g__RGIG1902;s__RGIG1902 sp945947915 | Bacteria | Bacillota | Clostridia | Oscillospirales | Butyricicoccaceae | *RGIG1902* | *RGIG1902 sp945947915* | GCA_945947915.1 | | 95.97 |
| grpH.bin.42 | d__Bacteria;p__Bacteroidota;c__Bacteroidia;o__Bacteroidales;f__Bacteroidaceae;g__Alloprevotella;s__Alloprevotella sp004552155 | Bacteria | Bacteroidota | Bacteroidia | Bacteroidales | Bacteroidaceae | *Alloprevotella* | *Alloprevotella sp004552155* | GCA_004552155.1 | | 96.57 |
| grpH.bin.46 | d__Bacteria;p__Bacillota;c__Negativicutes;o__Selenomonadales;f__Selenomonadaceae;g__Anaerovibrio;s__Anaerovibrio slackiae | Bacteria | Bacillota | Negativicutes | Selenomonadales | Selenomonadaceae | *Anaerovibrio* | *Anaerovibrio slackiae* | GCF_009695585.1 | | 98.03 |
| grpH.bin.50 | d__Bacteria;p__Bacillota;c__Clostridia;o__Oscillospirales;f__Acutalibacteraceae;g__UBA737;s__ | Bacteria | Bacillota | Clostridia | Oscillospirales | Acutalibacteraceae | *UBA737* |  | N/A |  | N/A |
| grpH.bin.53 | d__Bacteria;p__Bacteroidota;c__Bacteroidia;o__Bacteroidales;f__Rikenellaceae;g__Alistipes;s__Alistipes sp017403125 | Bacteria | Bacteroidota | Bacteroidia | Bacteroidales | Rikenellaceae | *Alistipes* | *Alistipes sp017403125* | GCA_017403125.1 | | 98.54 |
| grpH.bin.57 | d__Bacteria;p__Bacteroidota;c__Bacteroidia;o__Bacteroidales;f__Bacteroidaceae;g__Prevotella;s__Prevotella communis | Bacteria | Bacteroidota | Bacteroidia | Bacteroidales | Bacteroidaceae | *Prevotella* | *Prevotella communis* | GCF_022024115.1 | | 95.79 |
| grpH.bin.65 | d__Bacteria;p__Bacillota;c__Clostridia;o__Oscillospirales;f__CAG-272;g__Firm-07;s__Firm-07 sp017462615 | Bacteria | Bacillota | Clostridia | Oscillospirales | CAG-272 | *Firm-07* | *Firm-07 sp017462615* | GCA_017462615.1 | | 97.9 |
| grpH.bin.66 | d__Bacteria;p__Bacteroidota;c__Bacteroidia;o__Bacteroidales;f__Rikenellaceae;g__Alistipes;s__Alistipes sp015059955 | Bacteria | Bacteroidota | Bacteroidia | Bacteroidales | Rikenellaceae | *Alistipes* | *Alistipes sp015059955* | GCA_015059955.1 | | 97.12 |
| grpH.bin.71 | d__Bacteria;p__Bacteroidota;c__Bacteroidia;o__Bacteroidales;f__Rikenellaceae;g__Alistipes;s__Alistipes sp959604655 | Bacteria | Bacteroidota | Bacteroidia | Bacteroidales | Rikenellaceae | *Alistipes* | *Alistipes sp959604655* | GCA_959604655.1 | | 97.26 |
| grpH.bin.74 | d__Bacteria;p__Bacteroidota;c__Bacteroidia;o__Bacteroidales;f__Muribaculaceae;g__Lepagella;s__Lepagella sp905214735 | Bacteria | Bacteroidota | Bacteroidia | Bacteroidales | Muribaculaceae | *Lepagella* | *Lepagella sp905214735* | GCA_905214735.1 | | 98.48 |
| grpH.bin.80 | d__Bacteria;p__Bacillota;c__Clostridia;o__Lachnospirales;f__Anaerotignaceae;g__Anaerotignum_A;s__Anaerotignum_A sp945912105 | Bacteria | Bacillota | Clostridia | Lachnospirales | Anaerotignaceae | *Anaerotignum_A* | *Anaerotignum_A sp945912105* | GCA_945912105.1 | | 98.16 |
| grpH.bin.81 | d__Bacteria;p__Bacillota;c__Clostridia;o__Christensenellales;f__Borkfalkiaceae;g__Scatosoma;s__ | Bacteria | Bacillota | Clostridia | Christensenellales | Borkfalkiaceae | *Scatosoma* | | N/A |  | N/A |
| grpH.bin.84 | d__Bacteria;p__Bacteroidota;c__Bacteroidia;o__Bacteroidales;f__Tannerellaceae;g__Parabacteroides;s__Parabacteroides distasonis | Bacteria | Bacteroidota | Bacteroidia | Bacteroidales | Tannerellaceae | *Parabacteroides* | *Parabacteroides distasonis* | GCF_000012845.1 | | 97.88 |
| grpH.bin.95 | d__Bacteria;p__Bacillota;c__Clostridia;o__Oscillospirales;f__Ruminococcaceae;g__RGIG3102;s__ | Bacteria | Bacillota | Clostridia | Oscillospirales | Ruminococcaceae | *RGIG3102* | | N/A |  | N/A |
| grpH.bin.98 | d__Bacteria;p__Bacillota;c__Clostridia;o__Oscillospirales;f__Oscillospiraceae;g__Limivicinus;s__Limivicinus sp004560305 | Bacteria | Bacillota | Clostridia | Oscillospirales | Oscillospiraceae | *Limivicinus* | *Limivicinus sp004560305* | GCA_004560305.1 | | 96.78 |
| grpH.bin.99 | d__Bacteria;p__Bacillota;c__Clostridia;o__Lachnospirales;f__Lachnospiraceae;g__CAG-303;s__ | Bacteria | Bacillota | Clostridia | Lachnospirales | Lachnospiraceae | *CAG-303* |  | N/A |  | N/A |
| H1.bin.10 | d__Bacteria;p__Bacillota;c__Clostridia;o__Oscillospirales;f__Acutalibacteraceae;g__CAG-177;s__CAG-177 sp900771185 | Bacteria | Bacillota | Clostridia | Oscillospirales | Acutalibacteraceae | *CAG-177* | *CAG-177 sp900771185* | GCA_900771185.1 | | 95.49 |
| H1.bin.101 | d__Bacteria;p__Bacillota;c__Clostridia;o__Oscillospirales;f__Acutalibacteraceae;g__CAG-964;s__ | Bacteria | Bacillota | Clostridia | Oscillospirales | Acutalibacteraceae | *CAG-964* |  | N/A |  | N/A |
| H1.bin.103 | d__Bacteria;p__Bacillota;c__Clostridia;o__Lachnospirales;f__Lachnospiraceae;g__CAG-632;s__CAG-632 sp017623675 | Bacteria | Bacillota | Clostridia | Lachnospirales | Lachnospiraceae | *CAG-632* | *CAG-632 sp017623675* | GCA_017623675.1 | | 98.88 |
| H1.bin.108 | d__Bacteria;p__Elusimicrobiota;c__Elusimicrobia;o__Elusimicrobiales;f__Elusimicrobiaceae;g__UBA1174;s__UBA1174 sp900556855 | Bacteria | Elusimicrobiota | Elusimicrobia | Elusimicrobiales | Elusimicrobiaceae | *UBA1174* | *UBA1174 sp900556855* | GCA_934643455.1 | | 97.06 |
| H1.bin.109 | d__Bacteria;p__Bacillota;c__Clostridia;o__Oscillospirales;f__CAG-272;g__RGIG8775;s__ | Bacteria | Bacillota | Clostridia | Oscillospirales | CAG-272 | *RGIG8775* | | N/A |  | N/A |
| H1.bin.113 | d__Bacteria;p__Bacteroidota;c__Bacteroidia;o__Bacteroidales;f__Bacteroidaceae;g__Bacteroides;s__Bacteroides uniformis | Bacteria | Bacteroidota | Bacteroidia | Bacteroidales | Bacteroidaceae | *Bacteroides* | *Bacteroides uniformis* | GCF_025147485.1 | | 98.83 |
| H1.bin.114 | d__Bacteria;p__Bacillota;c__Bacilli;o__RF39;f__UBA660;g__CAG-1000;s__ | Bacteria | Bacillota | Bacilli | RF39 | UBA660 | *CAG-1000* | | N/A |  | N/A |
| H1.bin.117 | d__Bacteria;p__Bacteroidota;c__Bacteroidia;o__Bacteroidales;f__Paludibacteraceae;g__Colicola;s__Colicola sp900542355 | Bacteria | Bacteroidota | Bacteroidia | Bacteroidales | Paludibacteraceae | *Colicola* | *Colicola sp900542355* | GCA_900542355.1 | | 98.62 |
| H1.bin.120 | d__Bacteria;p__Spirochaetota;c__Spirochaetia;o__Treponematales;f__Treponemataceae;g__Treponema_D;s__ | Bacteria | Spirochaetota | Spirochaetia | Treponematales | Treponemataceae | *Treponema_D* | | N/A |  | N/A |
| H1.bin.125 | d__Bacteria;p__Bacillota;c__Clostridia;o__Lachnospirales;f__Lachnospiraceae;g__Coprococcus;s__ | Bacteria | Bacillota | Clostridia | Lachnospirales | Lachnospiraceae | *Coprococcus* | | N/A |  | N/A |
| H1.bin.13 | d__Bacteria;p__Bacillota;c__Clostridia;o__Christensenellales;f__Borkfalkiaceae;g__Coproplasma;s__ | Bacteria | Bacillota | Clostridia | Christensenellales | Borkfalkiaceae | *Coproplasma* | | N/A |  | N/A |
| H1.bin.15 | d__Bacteria;p__Pseudomonadota;c__Gammaproteobacteria;o__Enterobacterales;f__Succinivibrionaceae;g__Succinivibrio;s__Succinivibrio sp003456415 | Bacteria | Pseudomonadota | Gammaproteobacteria | Enterobacterales | Succinivibrionaceae | *Succinivibrio* | *Succinivibrio sp003456415* | GCA_902785325.1 | | 96.94 |
| H1.bin.16 | d__Bacteria;p__Bacillota;c__Clostridia;o__Oscillospirales;f__Oscillospiraceae;g__Faecousia;s__ | Bacteria | Bacillota | Clostridia | Oscillospirales | Oscillospiraceae | *Faecousia* |  | N/A |  | N/A |
| H1.bin.17 | d__Bacteria;p__Bacillota;c__Clostridia;o__Oscillospirales;f__Oscillospiraceae;g__Oscillibacter;s__ | Bacteria | Bacillota | Clostridia | Oscillospirales | Oscillospiraceae | *Oscillibacter* | | N/A |  | N/A |
| H1.bin.2 | d__Bacteria;p__Bacillota;c__Clostridia;o__Lachnospirales;f__Lachnospiraceae;g__JALENY01;s__JALENY01 sp036310185 | Bacteria | Bacillota | Clostridia | Lachnospirales | Lachnospiraceae | *JALENY01* | *JALENY01 sp036310185* | GCA_036310185.1 | | 99.12 |
| H1.bin.20 | d__Bacteria;p__Bacillota;c__Clostridia;o__Oscillospirales;f__Ruminococcaceae;g__Ruminococcus;s__Ruminococcus sp017543335 | Bacteria | Bacillota | Clostridia | Oscillospirales | Ruminococcaceae | *Ruminococcus* | *Ruminococcus sp017543335* | GCA_017543335.1 | | 97.93 |
| H1.bin.21 | d__Bacteria;p__Bacteroidota;c__Bacteroidia;o__Bacteroidales;f__Bacteroidaceae;g__UBA6382;s__UBA6382 sp900557555 | Bacteria | Bacteroidota | Bacteroidia | Bacteroidales | Bacteroidaceae | *UBA6382* | *UBA6382 sp900557555* | GCA_900557555.1 | | 98.24 |
| H1.bin.22 | d__Bacteria;p__Bacillota;c__Clostridia;o__Lachnospirales;f__Lachnospiraceae;g__Bovifimicola;s__ | Bacteria | Bacillota | Clostridia | Lachnospirales | Lachnospiraceae | *Bovifimicola* | | N/A |  | N/A |
| H1.bin.23 | d__Bacteria;p__Bacillota;c__Clostridia;o__Oscillospirales;f__Acutalibacteraceae;g__Fimenecus;s__Fimenecus sp937897215 | Bacteria | Bacillota | Clostridia | Oscillospirales | Acutalibacteraceae | *Fimenecus* | *Fimenecus sp937897215* | GCA_937897215.1 | | 97.49 |
| H1.bin.24 | d__Bacteria;p__Bacillota;c__Clostridia;o__Christensenellales;f__QALW01;g__UMGS1322;s__ | Bacteria | Bacillota | Clostridia | Christensenellales | QALW01 | *UMGS1322* | | N/A |  | N/A |
| H1.bin.25 | d__Bacteria;p__Bacteroidota;c__Bacteroidia;o__Bacteroidales;f__Muribaculaceae;g__JAGBWK01;s__JAGBWK01 sp001915385 | Bacteria | Bacteroidota | Bacteroidia | Bacteroidales | Muribaculaceae | *JAGBWK01* | *JAGBWK01 sp001915385* | GCA_001915385.1 | | 96.04 |
| H1.bin.26 | d__Bacteria;p__Bacillota;c__Clostridia;o__Oscillospirales;f__Acutalibacteraceae;g__Ruminococcoides;s__ | Bacteria | Bacillota | Clostridia | Oscillospirales | Acutalibacteraceae | *Ruminococcoides* | | N/A |  | N/A |
| H1.bin.29 | d__Bacteria;p__Bacillota;c__Clostridia;o__Oscillospirales;f__Ruminococcaceae;g__Ruminococcus;s__Ruminococcus sp002362135 | Bacteria | Bacillota | Clostridia | Oscillospirales | Ruminococcaceae | *Ruminococcus* | *Ruminococcus sp002362135* | GCA_002362135.1 | | 98.41 |
| H1.bin.35 | d__Bacteria;p__Verrucomicrobiota;c__Kiritimatiellia;o__RFP12;f__UBA1067;g__RUG572;s__RUG572 sp900547945 | Bacteria | Verrucomicrobiota | Kiritimatiellia | RFP12 | UBA1067 | *RUG572* | *RUG572 sp900547945* | GCA_900547945.1 | | 96.7 |
| H1.bin.36 | d__Bacteria;p__Bacillota;c__Clostridia;o__HGM11327;f__HGM11327;g__SIG351;s__SIG351 sp017935525 | Bacteria | Bacillota | Clostridia | HGM11327 | HGM11327 | *SIG351* | *SIG351 sp017935525* | GCA_017935525.1 | | 97.87 |
| H1.bin.38 | d__Bacteria;p__Bacillota;c__Clostridia;o__Lachnospirales;f__Lachnospiraceae;g__RGIG423;s__ | Bacteria | Bacillota | Clostridia | Lachnospirales | Lachnospiraceae | *RGIG423* |  | N/A |  | N/A |
| H1.bin.4 | d__Bacteria;p__Bacillota;c__Clostridia;o__Oscillospirales;f__Oscillospiraceae;g__Faecousia;s__ | Bacteria | Bacillota | Clostridia | Oscillospirales | Oscillospiraceae | *Faecousia* |  | N/A |  | N/A |
| H1.bin.40 | d__Bacteria;p__Bacteroidota;c__Bacteroidia;o__Bacteroidales;f__UBA932;g__Cryptobacteroides;s__Cryptobacteroides sp900316045 | Bacteria | Bacteroidota | Bacteroidia | Bacteroidales | UBA932 | *Cryptobacteroides* | *Cryptobacteroides sp900316045* | GCA_900316045.1 | | 98.63 |
| H1.bin.43 | d__Bacteria;p__Bacteroidota;c__Bacteroidia;o__Bacteroidales;f__Muribaculaceae;g__CAG-873;s__ | Bacteria | Bacteroidota | Bacteroidia | Bacteroidales | Muribaculaceae | *CAG-873* |  | N/A |  | N/A |
| H1.bin.44 | d__Bacteria;p__Bacillota;c__Clostridia;o__Oscillospirales;f__Acutalibacteraceae;g__Ruminococcoides;s__ | Bacteria | Bacillota | Clostridia | Oscillospirales | Acutalibacteraceae | *Ruminococcoides* | | N/A |  | N/A |
| H1.bin.46 | d__Bacteria;p__Bacillota;c__Clostridia;o__Oscillospirales;f__Acutalibacteraceae;g__UBA737;s__ | Bacteria | Bacillota | Clostridia | Oscillospirales | Acutalibacteraceae | *UBA737* |  | N/A |  | N/A |
| H1.bin.50 | d__Bacteria;p__Spirochaetota;c__Spirochaetia;o__Treponematales;f__Treponemataceae;g__Treponema_D;s__Treponema_D sp945873775 | Bacteria | Spirochaetota | Spirochaetia | Treponematales | Treponemataceae | *Treponema_D* | *Treponema_D sp945873775* | GCF_945873775.1 | | 98.41 |
| H1.bin.53 | d__Bacteria;p__Bacteroidota;c__Bacteroidia;o__Bacteroidales;f__UBA932;g__Cryptobacteroides;s__Cryptobacteroides sp900546445 | Bacteria | Bacteroidota | Bacteroidia | Bacteroidales | UBA932 | *Cryptobacteroides* | *Cryptobacteroides sp900546445* | GCA_900546445.1 | | 98.56 |
| H1.bin.54 | d__Bacteria;p__Bacillota;c__Clostridia;o__Lachnospirales;f__Lachnospiraceae;g__Roseburia_C;s__Roseburia_C amylophila | Bacteria | Bacillota | Clostridia | Lachnospirales | Lachnospiraceae | *Roseburia_C* | *Roseburia_C amylophila* | GCF_025567465.1 | | 96.54 |
| H1.bin.60 | d__Bacteria;p__Bacillota;c__Clostridia;o__Oscillospirales;f__CAG-272;g__Avispirillum;s__Avispirillum sp017623255 | Bacteria | Bacillota | Clostridia | Oscillospirales | CAG-272 | *Avispirillum* | *Avispirillum sp017623255* | GCA_017623255.1 | | 98.99 |
| H1.bin.62 | d__Bacteria;p__Cyanobacteriota;c__Vampirovibrionia;o__Gastranaerophilales;f__Gastranaerophilaceae;g__Scatousia;s__Scatousia sp900321895 | Bacteria | Cyanobacteriota | Vampirovibrionia | Gastranaerophilales | Gastranaerophilaceae | *Scatousia* | *Scatousia sp900321895* | GCA_900321895.1 | | 98.73 |
| H1.bin.64 | d__Bacteria;p__Bacillota;c__Clostridia;o__Oscillospirales;f__Acutalibacteraceae;g__DTU089;s__DTU089 sp017626295 | Bacteria | Bacillota | Clostridia | Oscillospirales | Acutalibacteraceae | *DTU089* | *DTU089 sp017626295* | GCA_017626295.1 | | 97.64 |
| H1.bin.7 | d__Bacteria;p__Bacillota;c__Clostridia;o__Oscillospirales;f__CAG-382;g__UMGS1052;s__ | Bacteria | Bacillota | Clostridia | Oscillospirales | CAG-382 | *UMGS1052* | | N/A |  | N/A |
| H1.bin.77 | d__Bacteria;p__Campylobacterota;c__Campylobacteria;o__Campylobacterales;f__Campylobacteraceae;g__Campylobacter;s__Campylobacter sp017506845 | Bacteria | Campylobacterota | Campylobacteria | Campylobacterales | Campylobacteraceae | *Campylobacter* | *Campylobacter sp017506845* | GCA_017506845.1 | | 97.14 |
| H1.bin.79 | d__Bacteria;p__Bacillota;c__Clostridia;o__Christensenellales;f__UBA1242;g__UBA6345;s__UBA6345 sp934746645 | Bacteria | Bacillota | Clostridia | Christensenellales | UBA1242 | *UBA6345* | *UBA6345 sp934746645* | GCA_934746645.1 | | 97.15 |
| H1.bin.8 | d__Bacteria;p__Bacillota;c__Clostridia;o__Oscillospirales;f__CAG-272;g__UBA1740;s__ | Bacteria | Bacillota | Clostridia | Oscillospirales | CAG-272 | *UBA1740* | | N/A |  | N/A |
| H1.bin.80 | d__Bacteria;p__Bacillota;c__Clostridia;o__Lachnospirales;f__Lachnospiraceae;g__Acetatifactor;s__Acetatifactor sp018385425 | Bacteria | Bacillota | Clostridia | Lachnospirales | Lachnospiraceae | *Acetatifactor* | *Acetatifactor sp018385425* | GCA_018385425.1 | | 99 |
| H1.bin.81 | d__Bacteria;p__Bacteroidota;c__Bacteroidia;o__Bacteroidales;f__Bacteroidaceae;g__Phocaeicola;s__Phocaeicola vulgatus | Bacteria | Bacteroidota | Bacteroidia | Bacteroidales | Bacteroidaceae | *Phocaeicola* | *Phocaeicola vulgatus* | GCF_964248265.1 | | 99.09 |
| H1.bin.82 | d__Bacteria;p__Bacillota;c__Clostridia;o__UMGS1883;f__UMGS1883;g__UMGS1883;s__ | Bacteria | Bacillota | Clostridia | UMGS1883 | UMGS1883 | *UMGS1883* | | N/A |  | N/A |
| H1.bin.85 | d__Bacteria;p__Bacillota;c__Clostridia;o__Oscillospirales;f__CAG-272;g__UMGS1865;s__ | Bacteria | Bacillota | Clostridia | Oscillospirales | CAG-272 | *UMGS1865* | | N/A |  | N/A |
| H1.bin.89 | d__Bacteria;p__Bacillota;c__Clostridia;o__Oscillospirales;f__CAG-272;g__QALR01;s__QALR01 sp017937845 | Bacteria | Bacillota | Clostridia | Oscillospirales | CAG-272 | *QALR01* | *QALR01 sp017937845* | GCA_017937845.1 | | 98.77 |
| H1.bin.91 | d__Bacteria;p__Bacteroidota;c__Bacteroidia;o__Bacteroidales;f__Muribaculaceae;g__CAG-873;s__CAG-873 sp017937625 | Bacteria | Bacteroidota | Bacteroidia | Bacteroidales | Muribaculaceae | *CAG-873* | *CAG-873 sp017937625* | GCA_017937625.1 | | 98.07 |
| H1.bin.94 | d__Bacteria;p__Bacillota;c__Clostridia;o__Oscillospirales;f__Acutalibacteraceae;g__RUG420;s__RUG420 sp900317085 | Bacteria | Bacillota | Clostridia | Oscillospirales | Acutalibacteraceae | *RUG420* | *RUG420 sp900317085* | GCA_900317085.1 | | 98.55 |
| H1.bin.95 | d__Bacteria;p__Bacillota;c__Clostridia;o__Oscillospirales;f__Oscillospiraceae;g__Oscillibacter;s__ | Bacteria | Bacillota | Clostridia | Oscillospirales | Oscillospiraceae | *Oscillibacter* | | N/A |  | N/A |
| H1.bin.96 | d__Bacteria;p__Bacillota;c__Clostridia;o__Oscillospirales;f__CAG-272;g__CAG-448;s__ | Bacteria | Bacillota | Clostridia | Oscillospirales | CAG-272 | *CAG-448* |  | N/A |  | N/A |
| H1.bin.97 | d__Bacteria;p__Bacillota;c__Peptococcia;o__Peptococcales;f__Peptococcaceae;g__UBA7185;s__UBA7185 sp945949655 | Bacteria | Bacillota | Peptococcia | Peptococcales | Peptococcaceae | *UBA7185* | *UBA7185 sp945949655* | GCA_945949655.1 | | 97.88 |
| H1.bin.98 | d__Bacteria;p__Bacillota;c__Clostridia;o__Oscillospirales;f__Oscillospiraceae;g__Faecousia;s__ | Bacteria | Bacillota | Clostridia | Oscillospirales | Oscillospiraceae | *Faecousia* |  | N/A |  | N/A |
| H2.bin.1 | d__Bacteria;p__Bacillota;c__Clostridia;o__Oscillospirales;f__CAG-272;g__HGM12713;s__ | Bacteria | Bacillota | Clostridia | Oscillospirales | CAG-272 | *HGM12713* | | N/A |  | N/A |
| H2.bin.10 | d__Bacteria;p__Bacillota;c__Clostridia;o__Lachnospirales;f__Anaerotignaceae;g__Anaerotignum_A;s__Anaerotignum_A sp945912105 | Bacteria | Bacillota | Clostridia | Lachnospirales | Anaerotignaceae | *Anaerotignum_A* | *Anaerotignum_A sp945912105* | GCA_945912105.1 | | 98.06 |
| H2.bin.105 | d__Bacteria;p__Bacillota;c__Clostridia;o__Lachnospirales;f__Lachnospiraceae;g__Bovifimicola;s__ | Bacteria | Bacillota | Clostridia | Lachnospirales | Lachnospiraceae | *Bovifimicola* | | N/A |  | N/A |
| H2.bin.106 | d__Bacteria;p__Bacillota;c__Clostridia;o__Christensenellales;f__Borkfalkiaceae;g__Scatosoma;s__ | Bacteria | Bacillota | Clostridia | Christensenellales | Borkfalkiaceae | *Scatosoma* | | N/A |  | N/A |
| H2.bin.109 | d__Bacteria;p__Bacillota;c__Clostridia;o__Oscillospirales;f__Oscillospiraceae;g__Faecousia;s__Faecousia sp945486035 | Bacteria | Bacillota | Clostridia | Oscillospirales | Oscillospiraceae | *Faecousia* | *Faecousia sp945486035* | GCA_945486035.1 | | 98.2 |
| H2.bin.11 | d__Bacteria;p__Bacillota;c__Clostridia;o__Oscillospirales;f__Oscillospiraceae;g__Faecousia;s__Faecousia sp017409645 | Bacteria | Bacillota | Clostridia | Oscillospirales | Oscillospiraceae | *Faecousia* | *Faecousia sp017409645* | GCA_017409645.1 | | 96.09 |
| H2.bin.113 | d__Bacteria;p__Bacteroidota;c__Bacteroidia;o__Bacteroidales;f__Bacteroidaceae;g__Prevotella;s__Prevotella sp017623655 | Bacteria | Bacteroidota | Bacteroidia | Bacteroidales | Bacteroidaceae | *Prevotella* | *Prevotella sp017623655* | GCA_017623655.1 | | 97.75 |
| H2.bin.115 | d__Bacteria;p__Bacillota;c__Clostridia;o__Oscillospirales;f__Acutalibacteraceae;g__DTU089;s__DTU089 sp017626295 | Bacteria | Bacillota | Clostridia | Oscillospirales | Acutalibacteraceae | *DTU089* | *DTU089 sp017626295* | GCA_017626295.1 | | 98.43 |
| H2.bin.116 | d__Bacteria;p__Bacteroidota;c__Bacteroidia;o__Bacteroidales;f__UBA932;g__Cryptobacteroides;s__Cryptobacteroides sp017938925 | Bacteria | Bacteroidota | Bacteroidia | Bacteroidales | UBA932 | *Cryptobacteroides* | *Cryptobacteroides sp017938925* | GCA_017938925.1 | | 97.11 |
| H2.bin.117 | d__Bacteria;p__Bacillota;c__Clostridia;o__Lachnospirales;f__Lachnospiraceae;g__Butyrivibrio_A;s__ | Bacteria | Bacillota | Clostridia | Lachnospirales | Lachnospiraceae | *Butyrivibrio_A* | | N/A |  | N/A |
| H2.bin.118 | d__Bacteria;p__Bacillota;c__Clostridia;o__Lachnospirales;f__Lachnospiraceae;g__SIG299;s__SIG299 sp017622795 | Bacteria | Bacillota | Clostridia | Lachnospirales | Lachnospiraceae | *SIG299* | *SIG299 sp017622795* | GCA_017622795.1 | | 98.46 |
| H2.bin.12 | d__Bacteria;p__Bacteroidota;c__Bacteroidia;o__Bacteroidales;f__UBA932;g__Cryptobacteroides;s__ | Bacteria | Bacteroidota | Bacteroidia | Bacteroidales | UBA932 | *Cryptobacteroides* | | N/A |  | N/A |
| H2.bin.127 | d__Bacteria;p__Bacillota;c__Clostridia;o__Oscillospirales;f__Oscillospiraceae;g__Faecousia;s__ | Bacteria | Bacillota | Clostridia | Oscillospirales | Oscillospiraceae | *Faecousia* |  | N/A |  | N/A |
| H2.bin.128 | d__Bacteria;p__Bacillota;c__Bacilli;o__RF39;f__UBA660;g__MGBC108787;s__ | Bacteria | Bacillota | Bacilli | RF39 | UBA660 | *MGBC108787* | | N/A |  | N/A |
| H2.bin.13 | d__Bacteria;p__Bacillota;c__Clostridia;o__Oscillospirales;f__Acutalibacteraceae;g__RGIG2206;s__ | Bacteria | Bacillota | Clostridia | Oscillospirales | Acutalibacteraceae | *RGIG2206* | | N/A |  | N/A |
| H2.bin.132 | d__Bacteria;p__Bacillota;c__Clostridia;o__Oscillospirales;f__Oscillospiraceae;g__Faecousia;s__ | Bacteria | Bacillota | Clostridia | Oscillospirales | Oscillospiraceae | *Faecousia* |  | N/A |  | N/A |
| H2.bin.133 | d__Bacteria;p__Bacillota;c__Clostridia;o__Lachnospirales;f__Lachnospiraceae;g__COE1;s__ | Bacteria | Bacillota | Clostridia | Lachnospirales | Lachnospiraceae | *COE1* |  | N/A |  | N/A |
| H2.bin.18 | d__Bacteria;p__Bacillota;c__Clostridia;o__Oscillospirales;f__Oscillospiraceae;g__Faecousia;s__ | Bacteria | Bacillota | Clostridia | Oscillospirales | Oscillospiraceae | *Faecousia* |  | N/A |  | N/A |
| H2.bin.19 | d__Bacteria;p__Bacteroidota;c__Bacteroidia;o__Bacteroidales;f__G3-4614;g__Caccoplasma_A;s__Caccoplasma_A sp017416695 | Bacteria | Bacteroidota | Bacteroidia | Bacteroidales | G3-4614 | *Caccoplasma_A* | *Caccoplasma_A sp017416695* | GCA_017416695.1 | | 97.9 |
| H2.bin.20 | d__Bacteria;p__Bacillota;c__Clostridia;o__Lachnospirales;f__Lachnospiraceae;g__CAG-303;s__ | Bacteria | Bacillota | Clostridia | Lachnospirales | Lachnospiraceae | *CAG-303* |  | N/A |  | N/A |
| H2.bin.26 | d__Bacteria;p__Bacteroidota;c__Bacteroidia;o__Bacteroidales;f__Rikenellaceae;g__Alistipes;s__Alistipes sp017442365 | Bacteria | Bacteroidota | Bacteroidia | Bacteroidales | Rikenellaceae | *Alistipes* | *Alistipes sp017442365* | GCA_017442365.1 | | 97.62 |
| H2.bin.28 | d__Bacteria;p__Bacillota;c__Clostridia;o__Lachnospirales;f__Lachnospiraceae;g__UBA3766;s__ | Bacteria | Bacillota | Clostridia | Lachnospirales | Lachnospiraceae | *UBA3766* | | N/A |  | N/A |
| H2.bin.29 | d__Bacteria;p__Bacillota;c__Clostridia;o__Lachnospirales;f__Lachnospiraceae;g__RGIG8767;s__ | Bacteria | Bacillota | Clostridia | Lachnospirales | Lachnospiraceae | *RGIG8767* | | N/A |  | N/A |
| H2.bin.30 | d__Bacteria;p__Bacillota;c__Clostridia;o__Oscillospirales;f__Acutalibacteraceae;g__CAG-177;s__ | Bacteria | Bacillota | Clostridia | Oscillospirales | Acutalibacteraceae | *CAG-177* |  | N/A |  | N/A |
| H2.bin.32 | d__Bacteria;p__Bacillota;c__Clostridia;o__Oscillospirales;f__Acutalibacteraceae;g__UBA7067;s__UBA7067 sp002493755 | Bacteria | Bacillota | Clostridia | Oscillospirales | Acutalibacteraceae | *UBA7067* | *UBA7067 sp002493755* | GCA_002493755.1 | | 97.31 |
| H2.bin.34 | d__Bacteria;p__Bacillota;c__Clostridia;o__Oscillospirales;f__CAG-272;g__SIG701;s__ | Bacteria | Bacillota | Clostridia | Oscillospirales | CAG-272 | *SIG701* |  | N/A |  | N/A |
| H2.bin.35 | d__Bacteria;p__Bacillota;c__Clostridia;o__Christensenellales;f__CAG-917;g__CAG-475;s__CAG-475 sp000434435 | Bacteria | Bacillota | Clostridia | Christensenellales | CAG-917 | *CAG-475* | *CAG-475 sp000434435* | GCA_000434435.1 | | 98.54 |
| H2.bin.36 | d__Bacteria;p__Bacillota;c__Clostridia;o__Oscillospirales;f__Oscillospiraceae;g__Faecousia;s__Faecousia sp946011705 | Bacteria | Bacillota | Clostridia | Oscillospirales | Oscillospiraceae | *Faecousia* | *Faecousia sp946011705* | GCA_946011705.1 | | 95.92 |
| H2.bin.4 | d__Bacteria;p__Bacillota;c__Clostridia;o__Lachnospirales;f__Lachnospiraceae;g__Agathobacter;s__Agathobacter sp029008875 | Bacteria | Bacillota | Clostridia | Lachnospirales | Lachnospiraceae | *Agathobacter* | *Agathobacter sp029008875* | GCA_029008875.1 | | 96.06 |
| H2.bin.40 | d__Bacteria;p__Campylobacterota;c__Campylobacteria;o__Campylobacterales;f__Helicobacteraceae;g__Helicobacter_F;s__Helicobacter_F sp002287135 | Bacteria | Campylobacterota | Campylobacteria | Campylobacterales | Helicobacteraceae | *Helicobacter_F* | *Helicobacter_F sp002287135* | GCF_002287135.1 | | 96.99 |
| H2.bin.43 | d__Bacteria;p__Bacillota;c__Clostridia;o__Oscillospirales;f__Acutalibacteraceae;g__Ruminococcoides;s__Ruminococcoides sp900755995 | Bacteria | Bacillota | Clostridia | Oscillospirales | Acutalibacteraceae | *Ruminococcoides* | *Ruminococcoides sp900755995* | GCA_900755995.1 | | 98.81 |
| H2.bin.44 | d__Bacteria;p__Campylobacterota;c__Campylobacteria;o__Campylobacterales;f__Campylobacteraceae;g__Campylobacter;s__Campylobacter sp017506845 | Bacteria | Campylobacterota | Campylobacteria | Campylobacterales | Campylobacteraceae | *Campylobacter* | *Campylobacter sp017506845* | GCA_017506845.1 | | 97.37 |
| H2.bin.46 | d__Bacteria;p__Bacillota;c__Clostridia;o__Lachnospirales;f__Lachnospiraceae;g__UBA2942;s__ | Bacteria | Bacillota | Clostridia | Lachnospirales | Lachnospiraceae | *UBA2942* | | N/A |  | N/A |
| H2.bin.48 | d__Bacteria;p__Bacillota;c__Clostridia;o__Oscillospirales;f__Ruminococcaceae;g__Ruminococcus;s__Ruminococcus sp900545125 | Bacteria | Bacillota | Clostridia | Oscillospirales | Ruminococcaceae | *Ruminococcus* | *Ruminococcus sp900545125* | GCA_900545125.1 | | 97.91 |
| H2.bin.53 | d__Bacteria;p__Bacillota;c__Clostridia;o__Oscillospirales;f__Ruminococcaceae;g__CAG-115;s__CAG-115 sp017465665 | Bacteria | Bacillota | Clostridia | Oscillospirales | Ruminococcaceae | *CAG-115* | *CAG-115 sp017465665* | GCA_017465665.1 | | 97.07 |
| H2.bin.56 | d__Bacteria;p__Bacillota;c__Clostridia;o__Monoglobales;f__Monoglobaceae;g__UMGS1820;s__ | Bacteria | Bacillota | Clostridia | Monoglobales | Monoglobaceae | *UMGS1820* | | N/A |  | N/A |
| H2.bin.57 | d__Bacteria;p__Bacillota;c__Clostridia;o__Oscillospirales;f__CAG-272;g__CAG-448;s__CAG-448 sp017621695 | Bacteria | Bacillota | Clostridia | Oscillospirales | CAG-272 | *CAG-448* | *CAG-448 sp017621695* | GCA_017621695.1 | | 98.12 |
| H2.bin.59 | d__Bacteria;p__Bacillota;c__Negativicutes;o__Acidaminococcales;f__Acidaminococcaceae;g__Phascolarctobacterium_A;s__Phascolarctobacterium_A sp900553055 | Bacteria | Bacillota | Negativicutes | Acidaminococcales | Acidaminococcaceae | *Phascolarctobacterium_A* | *Phascolarctobacterium_A sp900553055* | GCA_900553055.1 | | 95.18 |
| H2.bin.6 | d__Bacteria;p__Bacteroidota;c__Bacteroidia;o__Bacteroidales;f__Paludibacteraceae;g__Colicola;s__Colicola sp900320865 | Bacteria | Bacteroidota | Bacteroidia | Bacteroidales | Paludibacteraceae | *Colicola* | *Colicola sp900320865* | GCA_900320865.1 | | 96.99 |
| H2.bin.7 | d__Bacteria;p__Cyanobacteriota;c__Vampirovibrionia;o__Gastranaerophilales;f__Gastranaerophilaceae;g__Zag111;s__Zag111 sp017626055 | Bacteria | Cyanobacteriota | Vampirovibrionia | Gastranaerophilales | Gastranaerophilaceae | *Zag111* | *Zag111 sp017626055* | GCA_017626055.1 | | 98.3 |
| H2.bin.71 | d__Bacteria;p__Bacillota;c__Clostridia;o__Christensenellales;f__Borkfalkiaceae;g__Scatosoma;s__Scatosoma sp017626135 | Bacteria | Bacillota | Clostridia | Christensenellales | Borkfalkiaceae | *Scatosoma* | *Scatosoma sp017626135* | GCA_017626135.1 | | 98.79 |
| H2.bin.73 | d__Bacteria;p__Bacillota;c__Clostridia;o__Oscillospirales;f__Ruminococcaceae;g__Ruminococcus;s__ | Bacteria | Bacillota | Clostridia | Oscillospirales | Ruminococcaceae | *Ruminococcus* | | N/A |  | N/A |
| H2.bin.75 | d__Bacteria;p__Bacillota;c__Clostridia;o__Lachnospirales;f__Lachnospiraceae;g__Agathobacter;s__Agathobacter sp945971355 | Bacteria | Bacillota | Clostridia | Lachnospirales | Lachnospiraceae | *Agathobacter* | *Agathobacter sp945971355* | GCA_945971355.1 | | 97.8 |
| H2.bin.76 | d__Bacteria;p__Bacillota;c__Clostridia;o__Lachnospirales;f__Lachnospiraceae;g__RUG11237;s__RUG11237 sp902767135 | Bacteria | Bacillota | Clostridia | Lachnospirales | Lachnospiraceae | *RUG11237* | *RUG11237 sp902767135* | GCA_902767135.1 | | 96.99 |
| H2.bin.79 | d__Bacteria;p__Bacillota;c__Clostridia;o__Oscillospirales;f__Oscillospiraceae;g__Oscillibacter;s__ | Bacteria | Bacillota | Clostridia | Oscillospirales | Oscillospiraceae | *Oscillibacter* | | N/A |  | N/A |
| H2.bin.8 | d__Bacteria;p__Bacillota;c__Clostridia;o__Lachnospirales;f__Lachnospiraceae;g__Agathobacter;s__Agathobacter sp034100525 | Bacteria | Bacillota | Clostridia | Lachnospirales | Lachnospiraceae | *Agathobacter* | *Agathobacter sp034100525* | GCA_034100525.1 | | 95.52 |
| H2.bin.80 | d__Bacteria;p__Bacteroidota;c__Bacteroidia;o__Bacteroidales;f__Rikenellaceae;g__Alistipes;s__Alistipes sp015059845 | Bacteria | Bacteroidota | Bacteroidia | Bacteroidales | Rikenellaceae | *Alistipes* | *Alistipes sp015059845* | GCA_015059845.1 | | 97.78 |
| H2.bin.81 | d__Bacteria;p__Verrucomicrobiota;c__Verrucomicrobiia;o__Verrucomicrobiales;f__Akkermansiaceae;g__Akkermansia;s__Akkermansia sp017435365 | Bacteria | Verrucomicrobiota | Verrucomicrobiia | Verrucomicrobiales | Akkermansiaceae | *Akkermansia* | *Akkermansia sp017435365* | GCA_017435365.1 | | 96.9 |
| H2.bin.84 | d__Bacteria;p__Bacillota;c__Clostridia;o__Oscillospirales;f__Oscillospiraceae;g__Faecousia;s__ | Bacteria | Bacillota | Clostridia | Oscillospirales | Oscillospiraceae | *Faecousia* |  | N/A |  | N/A |
| H2.bin.86 | d__Bacteria;p__Bacteroidota;c__Bacteroidia;o__Bacteroidales;f__Rikenellaceae;g__Alistipes;s__Alistipes sp017398555 | Bacteria | Bacteroidota | Bacteroidia | Bacteroidales | Rikenellaceae | *Alistipes* | *Alistipes sp017398555* | GCA_017398555.1 | | 97.09 |
| H2.bin.87 | d__Bacteria;p__Bacillota;c__Clostridia;o__Oscillospirales;f__Oscillospiraceae;g__F23-B02;s__F23-B02 sp946405205 | Bacteria | Bacillota | Clostridia | Oscillospirales | Oscillospiraceae | *F23-B02* | *F23-B02 sp946405205* | GCA_946405205.1 | | 96.92 |
| H2.bin.89 | d__Bacteria;p__Bacillota;c__Clostridia;o__Oscillospirales;f__Acutalibacteraceae;g__UBA737;s__UBA737 sp017481865 | Bacteria | Bacillota | Clostridia | Oscillospirales | Acutalibacteraceae | *UBA737* | *UBA737 sp017481865* | GCA_017481865.1 | | 99.41 |
| H2.bin.90 | d__Bacteria;p__Bacillota;c__Clostridia;o__Oscillospirales;f__Oscillospiraceae;g__Faecousia;s__Faecousia sp945873295 | Bacteria | Bacillota | Clostridia | Oscillospirales | Oscillospiraceae | *Faecousia* | *Faecousia sp945873295* | GCA_945873295.1 | | 96.01 |
| H2.bin.95 | d__Bacteria;p__Bacillota;c__Clostridia;o__Oscillospirales;f__Oscillospiraceae;g__Faecousia;s__ | Bacteria | Bacillota | Clostridia | Oscillospirales | Oscillospiraceae | *Faecousia* |  | N/A |  | N/A |
| H3.bin.102 | d__Bacteria;p__Campylobacterota;c__Campylobacteria;o__Campylobacterales;f__Helicobacteraceae;g__Helicobacter_B;s__Helicobacter_B sp017502485 | Bacteria | Campylobacterota | Campylobacteria | Campylobacterales | Helicobacteraceae | *Helicobacter_B* | *Helicobacter_B sp017502485* | GCF_017502485.1 | | 99.37 |
| H3.bin.104 | d__Bacteria;p__Spirochaetota;c__Spirochaetia;o__Treponematales;f__Treponemataceae;g__Treponema_D;s__ | Bacteria | Spirochaetota | Spirochaetia | Treponematales | Treponemataceae | *Treponema_D* | | N/A |  | N/A |
| H3.bin.107 | d__Bacteria;p__Bacillota;c__Clostridia;o__Lachnospirales;f__Lachnospiraceae;g__MGBC100174;s__ | Bacteria | Bacillota | Clostridia | Lachnospirales | Lachnospiraceae | *MGBC100174* | | N/A |  | N/A |
| H3.bin.117 | d__Bacteria;p__Bacillota;c__Clostridia;o__Oscillospirales;f__CAG-272;g__CAG-448;s__CAG-448 sp017483125 | Bacteria | Bacillota | Clostridia | Oscillospirales | CAG-272 | *CAG-448* | *CAG-448 sp017483125* | GCA_017483125.1 | | 96.53 |
| H3.bin.119 | d__Bacteria;p__Bacillota;c__Clostridia;o__Lachnospirales;f__Lachnospiraceae;g__RGIG8767;s__ | Bacteria | Bacillota | Clostridia | Lachnospirales | Lachnospiraceae | *RGIG8767* | | N/A |  | N/A |
| H3.bin.121 | d__Bacteria;p__Bacillota;c__Clostridia;o__Oscillospirales;f__CAG-382;g__UMGS1052;s__ | Bacteria | Bacillota | Clostridia | Oscillospirales | CAG-382 | *UMGS1052* | | N/A |  | N/A |
| H3.bin.125 | d__Bacteria;p__Bacillota;c__Clostridia;o__Oscillospirales;f__CAG-382;g__UMGS1387;s__UMGS1387 sp017620655 | Bacteria | Bacillota | Clostridia | Oscillospirales | CAG-382 | *UMGS1387* | *UMGS1387 sp017620655* | GCA_017620655.1 | | 98.12 |
| H3.bin.127 | d__Bacteria;p__Bacillota;c__Clostridia;o__Oscillospirales;f__Ruminococcaceae;g__CAG-115;s__ | Bacteria | Bacillota | Clostridia | Oscillospirales | Ruminococcaceae | *CAG-115* |  | N/A |  | N/A |
| H3.bin.128 | d__Bacteria;p__Bacillota;c__Clostridia;o__Oscillospirales;f__Acutalibacteraceae;g__CAG-964;s__ | Bacteria | Bacillota | Clostridia | Oscillospirales | Acutalibacteraceae | *CAG-964* |  | N/A |  | N/A |
| H3.bin.130 | d__Bacteria;p__Bacillota;c__Clostridia;o__Monoglobales;f__Monoglobaceae;g__;s__ | Bacteria | Bacillota | Clostridia | Monoglobales | *Monoglobaceae* | |  | N/A |  | N/A |
| H3.bin.134 | d__Bacteria;p__Bacillota;c__Clostridia;o__Lachnospirales;f__Lachnospiraceae;g__UBA3282;s__UBA3282 sp022781395 | Bacteria | Bacillota | Clostridia | Lachnospirales | Lachnospiraceae | *UBA3282* | *UBA3282 sp022781395* | GCA_022781395.1 | | 98.2 |
| H3.bin.135 | d__Bacteria;p__Bacillota;c__Clostridia;o__Oscillospirales;f__Acutalibacteraceae;g__CAG-488;s__ | Bacteria | Bacillota | Clostridia | Oscillospirales | Acutalibacteraceae | *CAG-488* |  | N/A |  | N/A |
| H3.bin.14 | d__Bacteria;p__Bacillota;c__Clostridia;o__Oscillospirales;f__Acutalibacteraceae;g__RUG806;s__ | Bacteria | Bacillota | Clostridia | Oscillospirales | Acutalibacteraceae | *RUG806* |  | N/A |  | N/A |
| H3.bin.150 | d__Bacteria;p__Bacteroidota;c__Bacteroidia;o__Bacteroidales;f__UBA932;g__Cryptobacteroides;s__Cryptobacteroides sp017938925 | Bacteria | Bacteroidota | Bacteroidia | Bacteroidales | UBA932 | *Cryptobacteroides* | *Cryptobacteroides sp017938925* | GCA_017938925.1 | | 96.33 |
| H3.bin.154 | d__Bacteria;p__Bacteroidota;c__Bacteroidia;o__Bacteroidales;f__Muribaculaceae;g__Duncaniella;s__Duncaniella sp910588795 | Bacteria | Bacteroidota | Bacteroidia | Bacteroidales | Muribaculaceae | *Duncaniella* | *Duncaniella sp910588795* | GCF_910588795.1 | | 99.05 |
| H3.bin.156 | d__Bacteria;p__Bacillota;c__Clostridia;o__Peptostreptococcales;f__Anaerovoracaceae;g__RGIG9096;s__RGIG9096 sp017936025 | Bacteria | Bacillota | Clostridia | Peptostreptococcales | Anaerovoracaceae | *RGIG9096* | *RGIG9096 sp017936025* | GCA_017936025.1 | | 98.77 |
| H3.bin.157 | d__Bacteria;p__Bacteroidota;c__Bacteroidia;o__Bacteroidales;f__Bacteroidaceae;g__Phocaeicola;s__ | Bacteria | Bacteroidota | Bacteroidia | Bacteroidales | Bacteroidaceae | *Phocaeicola* | | N/A |  | N/A |
| H3.bin.158 | d__Bacteria;p__Bacillota;c__Clostridia;o__Lachnospirales;f__Lachnospiraceae;g__UBA2882;s__ | Bacteria | Bacillota | Clostridia | Lachnospirales | Lachnospiraceae | *UBA2882* | | N/A |  | N/A |
| H3.bin.161 | d__Bacteria;p__Pseudomonadota;c__Alphaproteobacteria;o__Rickettsiales;f__UBA1997;g__RGIG8782;s__RGIG8782 sp017620845 | Bacteria | Pseudomonadota | Alphaproteobacteria | Rickettsiales | UBA1997 | *RGIG8782* | *RGIG8782 sp017620845* | GCA_017620845.1 | | 97.92 |
| H3.bin.162 | d__Bacteria;p__Bacteroidota;c__Bacteroidia;o__Bacteroidales;f__Bacteroidaceae;g__Prevotella;s__Prevotella sp902798615 | Bacteria | Bacteroidota | Bacteroidia | Bacteroidales | Bacteroidaceae | *Prevotella* | *Prevotella sp902798615* | GCA_902798615.1 | | 96.82 |
| H3.bin.163 | d__Bacteria;p__Bacillota;c__Clostridia;o__Christensenellales;f__Aristaeellaceae;g__Limiplasma;s__ | Bacteria | Bacillota | Clostridia | Christensenellales | Aristaeellaceae | *Limiplasma* | | N/A |  | N/A |
| H3.bin.166 | d__Bacteria;p__Bacillota;c__Clostridia;o__Oscillospirales;f__Oscillospiraceae;g__Faecousia;s__ | Bacteria | Bacillota | Clostridia | Oscillospirales | Oscillospiraceae | *Faecousia* |  | N/A |  | N/A |
| H3.bin.168 | d__Bacteria;p__Bacillota;c__Bacilli;o__RF39;f__UBA660;g__RGIG4266;s__RGIG4266 sp017461465 | Bacteria | Bacillota | Bacilli | RF39 | UBA660 | *RGIG4266* | *RGIG4266 sp017461465* | GCA_017461465.1 | | 98.73 |
| H3.bin.172 | d__Bacteria;p__Spirochaetota;c__Spirochaetia;o__Treponematales;f__Treponemataceae;g__Treponema_D;s__Treponema_D porcinum | Bacteria | Spirochaetota | Spirochaetia | Treponematales | Treponemataceae | *Treponema_D* | *Treponema_D porcinum* | GCF_900167145.1 | | 98.58 |
| H3.bin.19 | d__Bacteria;p__Bacillota;c__Clostridia;o__Christensenellales;f__Aristaeellaceae;g__Limiplasma;s__ | Bacteria | Bacillota | Clostridia | Christensenellales | Aristaeellaceae | *Limiplasma* | | N/A |  | N/A |
| H3.bin.21 | d__Bacteria;p__Bacillota;c__Clostridia;o__Lachnospirales;f__Anaerotignaceae;g__Anaerotignum_A;s__Anaerotignum_A sp945912105 | Bacteria | Bacillota | Clostridia | Lachnospirales | Anaerotignaceae | *Anaerotignum_A* | *Anaerotignum_A sp945912105* | GCA_945912105.1 | | 98.14 |
| H3.bin.23 | d__Bacteria;p__Bacillota;c__Clostridia;o__Christensenellales;f__Borkfalkiaceae;g__Scatosoma;s__ | Bacteria | Bacillota | Clostridia | Christensenellales | Borkfalkiaceae | *Scatosoma* | | N/A |  | N/A |
| H3.bin.26 | d__Bacteria;p__Bacillota;c__Clostridia;o__Oscillospirales;f__CAG-272;g__HGM12713;s__HGM12713 sp017504545 | Bacteria | Bacillota | Clostridia | Oscillospirales | CAG-272 | *HGM12713* | *HGM12713 sp017504545* | GCA_017504545.1 | | 97.24 |
| H3.bin.28 | d__Bacteria;p__Bacteroidota;c__Bacteroidia;o__Bacteroidales;f__UBA932;g__Egerieousia;s__Egerieousia sp015060445 | Bacteria | Bacteroidota | Bacteroidia | Bacteroidales | UBA932 | *Egerieousia* | *Egerieousia sp015060445* | GCA_015060445.1 | | 97.79 |
| H3.bin.30 | d__Bacteria;p__Bacillota;c__Clostridia;o__Lachnospirales;f__Lachnospiraceae;g__RGIG989;s__RGIG989 sp017390525 | Bacteria | Bacillota | Clostridia | Lachnospirales | Lachnospiraceae | *RGIG989* | *RGIG989 sp017390525* | GCA_017390525.1 | | 99.11 |
| H3.bin.34 | d__Bacteria;p__Bacillota;c__Clostridia;o__Oscillospirales;f__Oscillospiraceae;g__Vescimonas;s__ | Bacteria | Bacillota | Clostridia | Oscillospirales | Oscillospiraceae | *Vescimonas* | | N/A |  | N/A |
| H3.bin.36 | d__Bacteria;p__Bacillota;c__Clostridia;o__Oscillospirales;f__Acutalibacteraceae;g__UBA1081;s__ | Bacteria | Bacillota | Clostridia | Oscillospirales | Acutalibacteraceae | *UBA1081* | | N/A |  | N/A |
| H3.bin.39 | d__Bacteria;p__Bacillota;c__Clostridia;o__Oscillospirales;f__Ruminococcaceae;g__Ruminiclostridium_E;s__ | Bacteria | Bacillota | Clostridia | Oscillospirales | Ruminococcaceae | *Ruminiclostridium_E* | | N/A |  | N/A |
| H3.bin.4 | d__Bacteria;p__Bacteroidota;c__Bacteroidia;o__Bacteroidales;f__UBA932;g__Egerieousia;s__ | Bacteria | Bacteroidota | Bacteroidia | Bacteroidales | UBA932 | *Egerieousia* | | N/A |  | N/A |
| H3.bin.43 | d__Bacteria;p__Bacillota;c__Negativicutes;o__Acidaminococcales;f__Acidaminococcaceae;g__Phascolarctobacterium_A;s__Phascolarctobacterium_A sp030540395 | Bacteria | Bacillota | Negativicutes | Acidaminococcales | Acidaminococcaceae | *Phascolarctobacterium_A* | *Phascolarctobacterium_A sp030540395* | GCA_030540395.1 | | 98.65 |
| H3.bin.45 | d__Bacteria;p__Bacillota;c__Clostridia;o__Oscillospirales;f__CAG-272;g__UMGS1696;s__ | Bacteria | Bacillota | Clostridia | Oscillospirales | CAG-272 | *UMGS1696* | | N/A |  | N/A |
| H3.bin.50 | d__Bacteria;p__Bacteroidota;c__Bacteroidia;o__Bacteroidales;f__Muribaculaceae;g__JAJPXC01;s__JAJPXC01 sp947625935 | Bacteria | Bacteroidota | Bacteroidia | Bacteroidales | Muribaculaceae | *JAJPXC01* | *JAJPXC01 sp947625935* | GCA_947625935.1 | | 98.21 |
| H3.bin.53 | d__Bacteria;p__Bacteroidota;c__Bacteroidia;o__Bacteroidales;f__Muribaculaceae;g__Amulumruptor;s__Amulumruptor sp900539915 | Bacteria | Bacteroidota | Bacteroidia | Bacteroidales | Muribaculaceae | *Amulumruptor* | *Amulumruptor sp900539915* | GCA_900539915.1 | | 98.65 |
| H3.bin.54 | d__Bacteria;p__Bacillota;c__Clostridia;o__Oscillospirales;f__CAG-272;g__SIG701;s__ | Bacteria | Bacillota | Clostridia | Oscillospirales | CAG-272 | *SIG701* |  | N/A |  | N/A |
| H3.bin.56 | d__Bacteria;p__Bacillota;c__Clostridia;o__Oscillospirales;f__CAG-272;g__Firm-07;s__Firm-07 sp015065135 | Bacteria | Bacillota | Clostridia | Oscillospirales | CAG-272 | *Firm-07* | *Firm-07 sp015065135* | GCA_015065135.1 | | 97.59 |
| H3.bin.57 | d__Bacteria;p__Bacillota;c__Clostridia;o__Lachnospirales;f__Lachnospiraceae;g__NK4A136;s__NK4A136 sp000687675 | Bacteria | Bacillota | Clostridia | Lachnospirales | Lachnospiraceae | *NK4A136* | *NK4A136 sp000687675* | GCA_000687675.1 | | 96.55 |
| H3.bin.6 | d__Bacteria;p__Bacillota;c__Clostridia;o__Oscillospirales;f__CAG-272;g__RGIG3947;s__ | Bacteria | Bacillota | Clostridia | Oscillospirales | CAG-272 | *RGIG3947* | | N/A |  | N/A |
| H3.bin.60 | d__Bacteria;p__Bacillota;c__Clostridia;o__Lachnospirales;f__Lachnospiraceae;g__Laedolimicola;s__ | Bacteria | Bacillota | Clostridia | Lachnospirales | Lachnospiraceae | *Laedolimicola* | | N/A |  | N/A |
| H3.bin.61 | d__Bacteria;p__Bacillota;c__Clostridia;o__Oscillospirales;f__CAG-272;g__QALR01;s__QALR01 sp017937845 | Bacteria | Bacillota | Clostridia | Oscillospirales | CAG-272 | *QALR01* | *QALR01 sp017937845* | GCA_017937845.1 | | 98.55 |
| H3.bin.62 | d__Bacteria;p__Bacillota;c__Clostridia;o__Oscillospirales;f__CAG-272;g__UBA1740;s__UBA1740 sp017504405 | Bacteria | Bacillota | Clostridia | Oscillospirales | CAG-272 | *UBA1740* | *UBA1740 sp017504405* | GCA_017504405.1 | | 97.33 |
| H3.bin.65 | d__Bacteria;p__Bacillota;c__Bacilli;o__RF39;f__UBA660;g__CAG-533;s__CAG-533 sp900553855 | Bacteria | Bacillota | Bacilli | RF39 | UBA660 | *CAG-533* | *CAG-533 sp900553855* | GCA_900553855.1 | | 96.88 |
| H3.bin.68 | d__Bacteria;p__Bacteroidota;c__Bacteroidia;o__Bacteroidales;f__Tannerellaceae;g__Parabacteroides;s__Parabacteroides sp900760525 | Bacteria | Bacteroidota | Bacteroidia | Bacteroidales | Tannerellaceae | *Parabacteroides* | *Parabacteroides sp900760525* | GCA_041225035.1 | | 98.87 |
| H3.bin.7 | d__Bacteria;p__Bacteroidota;c__Bacteroidia;o__Bacteroidales;f__Bacteroidaceae;g__Prevotella;s__Prevotella sp002251295 | Bacteria | Bacteroidota | Bacteroidia | Bacteroidales | Bacteroidaceae | *Prevotella* | *Prevotella sp002251295* | GCF_002251295.1 | | 98.71 |
| H3.bin.70 | d__Bacteria;p__Bacillota;c__Clostridia;o__Oscillospirales;f__CAG-272;g__RGIG2000;s__RGIG2000 sp017416885 | Bacteria | Bacillota | Clostridia | Oscillospirales | CAG-272 | *RGIG2000* | *RGIG2000 sp017416885* | GCA_017416885.1 | | 97.28 |
| H3.bin.8 | d__Bacteria;p__Bacteroidota;c__Bacteroidia;o__Bacteroidales;f__Rikenellaceae;g__Alistipes;s__Alistipes sp900548155 | Bacteria | Bacteroidota | Bacteroidia | Bacteroidales | Rikenellaceae | *Alistipes* | *Alistipes sp900548155* | GCA_905213895.1 | | 96.41 |
| H3.bin.81 | d__Bacteria;p__Bacteroidota;c__Bacteroidia;o__Bacteroidales;f__Muribaculaceae;g__CAG-873;s__CAG-873 sp900554265 | Bacteria | Bacteroidota | Bacteroidia | Bacteroidales | Muribaculaceae | *CAG-873* | *CAG-873 sp900554265* | GCA_900554265.1 | | 96.64 |
| H3.bin.83 | d__Bacteria;p__Bacillota;c__Clostridia;o__Lachnospirales;f__Lachnospiraceae;g__Suilimivivens;s__Suilimivivens sp017622575 | Bacteria | Bacillota | Clostridia | Lachnospirales | Lachnospiraceae | *Suilimivivens* | *Suilimivivens sp017622575* | GCA_017622575.1 | | 99.28 |
| H3.bin.84 | d__Bacteria;p__Bacillota;c__Clostridia;o__Oscillospirales;f__Acutalibacteraceae;g__UBA1213;s__ | Bacteria | Bacillota | Clostridia | Oscillospirales | Acutalibacteraceae | *UBA1213* | | N/A |  | N/A |
| H3.bin.85 | d__Bacteria;p__Bacteroidota;c__Bacteroidia;o__Bacteroidales;f__Muribaculaceae;g__CAG-873;s__CAG-873 sp948665475 | Bacteria | Bacteroidota | Bacteroidia | Bacteroidales | Muribaculaceae | *CAG-873* | *CAG-873 sp948665475* | GCA_948665475.1 | | 96.33 |
| H3.bin.88 | d__Bacteria;p__Bacillota;c__Clostridia;o__TANB77;f__CAG-508;g__CAG-492;s__ | Bacteria | Bacillota | Clostridia | TANB77 | CAG-508 | *CAG-492* |  | N/A |  | N/A |
| H3.bin.89 | d__Bacteria;p__Bacillota;c__Clostridia;o__Oscillospirales;f__Ruminococcaceae;g__Ruminococcus_C;s__ | Bacteria | Bacillota | Clostridia | Oscillospirales | Ruminococcaceae | *Ruminococcus_C* | | N/A |  | N/A |
| H3.bin.90 | d__Bacteria;p__Bacteroidota;c__Bacteroidia;o__Bacteroidales;f__Muribaculaceae;g__CAG-873;s__ | Bacteria | Bacteroidota | Bacteroidia | Bacteroidales | Muribaculaceae | *CAG-873* |  | N/A |  | N/A |
| H3.bin.92 | d__Bacteria;p__Pseudomonadota;c__Gammaproteobacteria;o__Enterobacterales;f__Succinivibrionaceae;g__Succinivibrio;s__ | Bacteria | Pseudomonadota | Gammaproteobacteria | Enterobacterales | Succinivibrionaceae | *Succinivibrio* | | N/A |  | N/A |
| H3.bin.95 | d__Bacteria;p__Bacillota;c__Clostridia;o__Oscillospirales;f__Acutalibacteraceae;g__UMGS1976;s__ | Bacteria | Bacillota | Clostridia | Oscillospirales | Acutalibacteraceae | *UMGS1976* | | N/A |  | N/A |
| H3.bin.97 | d__Bacteria;p__Verrucomicrobiota;c__Lentisphaeria;o__Victivallales;f__UBA1829;g__SIG77;s__ | Bacteria | Verrucomicrobiota | Lentisphaeria | Victivallales | UBA1829 | *SIG77* |  | N/A |  | N/A |
