# Supplementary material for: Effects of diarrhea and antibiotic-induced microbial elimination on dynamic changes in fecal microbial communities and antibiotic resistance of Hu sheep lambs (Ovis aries)
Source: PeerJ. 2026 Jul 31;14:e21574. doi: 10.7717/peerj.21574 (PMC13431306; doi:10.7717/peerj.21574)
Supplement: Supplemental Information 5 [file peerj-14-21574-s005.docx]

| Supplementary Table S4. The MAGs with significant difference in hu sheep fecal metagenomes. | | | | | | | | | |
| --- | --- | --- | --- | --- | --- | --- | --- | --- | --- |
| MAGs | DL_mean | DM_mean | H_mean | DL_vs_DM_p | DL_vs_DM_p significance | DL_vs_H_p | DL_vs_H_p significance | DM_vs_H_p | DM_vs_H_p significance |
| DL1.bin.17 | 292861 | 1323.39 | 2141.64 | 0.00023 | *** | 0.00019 | *** | 0.40444 |  |
| DL1.bin.23 | 39025.3 | 0 | 0 | 0.00059 | *** | 0.00059 | *** |  |  |
| DL1.bin.28 | 10518.2 | 0 | 0 | 0.00087 | *** | 0.00087 | *** |  |  |
| DL1.bin.35 | 15691.6 | 0 | 0 | 0.00252 | ** | 0.00252 | ** |  |  |
| DL2.bin.13 | 6348.63 | 0 | 0 | 5.8E-05 | *** | 5.8E-05 | *** |  |  |
| DL2.bin.18 | 31184.9 | 123.484 | 92.8452 | 0.00371 | ** | 0.00375 | ** | 0.85318 |  |
| DL2.bin.21 | 14526.3 | 718.484 | 824.637 | 6.6E-06 | *** | 1.3E-05 | *** | 0.74302 |  |
| DL2.bin.22 | 17092.2 | 2684.57 | 305.384 | 0.00062 | *** | 0.0013 | ** | 0.14719 |  |
| DL2.bin.26 | 8738.69 | 1118.33 | 1151.67 | 0.00618 | ** | 0.0051 | ** | 0.78654 |  |
| DL2.bin.28 | 31146 | 189.731 | 292.281 | 0.00302 | ** | 0.00311 | ** | 0.39879 |  |
| DL2.bin.29 | 32136.8 | 536.551 | 552.612 | 0.00186 | ** | 0.00187 | ** | 0.83794 |  |
| DL2.bin.3 | 10816.4 | 0 | 0 | 0.00142 | ** | 0.00142 | ** |  |  |
| DL2.bin.30 | 21555.4 | 0 | 96.9054 | 0.00846 | ** | 0.00842 | ** | 0.42265 |  |
| DL2.bin.33 | 105585 | 1633.09 | 1537.23 | 0.00031 | *** | 0.00025 | *** | 0.93135 |  |
| DL2.bin.36 | 4731.52 | 0 | 0 | 0.00111 | ** | 0.00111 | ** |  |  |
| DL2.bin.38 | 5591.61 | 0 | 101.582 | 0.0044 | ** | 0.00274 | ** | 0.42265 |  |
| DL2.bin.6 | 15998.5 | 0 | 0 | 0.0433 | * | 0.0433 | * |  |  |
| DL3.bin.10 | 20304.2 | 792.97 | 688.381 | 0.00683 | ** | 0.00688 | ** | 0.40876 |  |
| DL3.bin.24 | 23876.7 | 168.827 | 307.948 | 2E-07 | *** | 6E-08 | *** | 0.50428 |  |
| DL3.bin.25 | 4836.08 | 0 | 0 | 0.00162 | ** | 0.00162 | ** |  |  |
| DL3.bin.35 | 16696 | 0 | 0 | 5.1E-05 | *** | 5.1E-05 | *** |  |  |
| DL3.bin.38 | 66754.6 | 200.904 | 277.836 | 0.00092 | *** | 0.00091 | *** | 0.29248 |  |
| DL3.bin.40 | 6756.67 | 0 | 0 | 0.00472 | ** | 0.00472 | ** |  |  |
| DL3.bin.6 | 4044.29 | 0 | 0 | 9.8E-05 | *** | 9.8E-05 | *** |  |  |
| DL3.bin.7 | 15037.3 | 967.507 | 1169.35 | 0.00282 | ** | 0.00305 | ** | 0.28089 |  |
| DM1.bin.10 | 0 | 2393.67 | 1304.84 | 0.1392 |  | 0.01639 | * | 0.39042 |  |
| DM1.bin.102 | 0 | 7637.19 | 0 | 0.05634 |  |  |  | 0.05634 |  |
| DM1.bin.104 | 0 | 3605.14 | 1085.8 | 0.11749 |  | 0.02943 | * | 0.20288 |  |
| DM1.bin.105 | 0 | 1211.88 | 108.909 | 0.21593 |  | 0.42265 |  | 0.24359 |  |
| DM1.bin.107 | 0 | 5405.93 | 2396.98 | 0.09846 |  | 0.04237 | * | 0.23834 |  |
| DM1.bin.112 | 0 | 1657.83 | 0 | 0.19024 |  |  |  | 0.19024 |  |
| DM1.bin.118 | 0 | 14903.3 | 3891.34 | 0.19248 |  | 0.36007 |  | 0.28873 |  |
| DM1.bin.12 | 0 | 1988.34 | 881.102 | 0.05602 |  | 0.18598 |  | 0.17081 |  |
| DM1.bin.127 | 0 | 1797.26 | 200.23 | 0.0968 |  | 0.42265 |  | 0.10649 |  |
| DM1.bin.13 | 254.837 | 3991.09 | 0 | 0.00072 | *** | 0.00088 | *** | 0.00066 | *** |
| DM1.bin.131 | 0 | 2633.11 | 1228.41 | 0.18412 |  | 0.34623 |  | 0.44793 |  |
| DM1.bin.133 | 0 | 293.881 | 146.025 | 0.04974 | * | 0.01569 | * | 0.15462 |  |
| DM1.bin.134 | 0 | 6595.13 | 2071.57 | 0.10844 |  | 0.07475 |  | 0.1908 |  |
| DM1.bin.139 | 0 | 12362.4 | 862.855 | 0.1752 |  | 0.00041 | *** | 0.19499 |  |
| DM1.bin.141 | 0 | 1522.75 | 1516.02 | 0.16467 |  | 0.29388 |  | 0.99611 |  |
| DM1.bin.22 | 0 | 408.98 | 105.764 | 0.19025 |  | 0.2037 |  | 0.28241 |  |
| DM1.bin.38 | 0 | 24078.2 | 12277.3 | 0.19544 |  | 0.18144 |  | 0.46232 |  |
| DM1.bin.39 | 0 | 1913.84 | 1074.85 | 0.00276 | ** | 0.27836 |  | 0.36873 |  |
| DM1.bin.44 | 0 | 5548.11 | 1350.91 | 0.07967 |  | 0.11062 |  | 0.11854 |  |
| DM1.bin.54 | 0 | 2460.09 | 402.347 | 0.18188 |  | 0.01211 | * | 0.23429 |  |
| DM1.bin.60 | 0 | 5990.22 | 3517.62 | 0.0263 | * | 0.17175 |  | 0.2889 |  |
| DM1.bin.62 | 363.193 | 4462.57 | 728.368 | 0.12802 |  | 0.27993 |  | 0.14575 |  |
| DM1.bin.68 | 0 | 317.962 | 201.058 | 0.0666 |  | 0.09347 |  | 0.34718 |  |
| DM1.bin.71 | 0 | 10459.1 | 2777.91 | 0.11896 |  | 0.20683 |  | 0.18363 |  |
| DM1.bin.73 | 0 | 987.908 | 488.864 | 0.05716 |  | 0.14684 |  | 0.20114 |  |
| DM1.bin.75 | 0 | 3976.94 | 1550.75 | 0.20126 |  | 0.42265 |  | 0.41212 |  |
| DM1.bin.77 | 0 | 1271.67 | 1066.86 | 0.02394 | * | 0.13292 |  | 0.69863 |  |
| DM1.bin.78 | 0 | 1031.84 | 82.2319 | 0.06726 |  | 0.42265 |  | 0.06805 |  |
| DM1.bin.83 | 0 | 6179.42 | 2480.79 | 0.08 |  | 0.23983 |  | 0.19984 |  |
| DM1.bin.85 | 0 | 4887.62 | 283.714 | 0.18425 |  | 0.19073 |  | 0.20068 |  |
| DM1.bin.98 | 0 | 6719.04 | 6877.87 | 0.07571 |  | 0.16398 |  | 0.96861 |  |
| DM2.bin.100 | 0 | 2956.15 | 476.138 | 0.13647 |  | 0.03971 | * | 0.17851 |  |
| DM2.bin.104 | 0 | 3287.49 | 135.846 | 0.19109 |  | 0.42265 |  | 0.20245 |  |
| DM2.bin.105 | 0 | 8079.88 | 5786.49 | 0.02096 | * | 0.26882 |  | 0.61578 |  |
| DM2.bin.113 | 0 | 1299.89 | 455.106 | 0.20333 |  | 0.10706 |  | 0.34917 |  |
| DM2.bin.121 | 0 | 18951.3 | 2009.73 | 0.1728 |  | 0.06474 |  | 0.20342 |  |
| DM2.bin.18 | 0 | 239.615 | 130.102 | 0.00525 | ** | 0.22774 |  | 0.2827 |  |
| DM2.bin.23 | 0 | 3252.19 | 4982.86 | 0.07305 |  | 0.19191 |  | 0.57922 |  |
| DM2.bin.28 | 0 | 7474.81 | 1232.8 | 0.16436 |  | 0.03135 | * | 0.21381 |  |
| DM2.bin.36 | 0 | 1102.83 | 96.1457 | 0.04123 | * | 0.42265 |  | 0.03421 | * |
| DM2.bin.50 | 0 | 249.713 | 122.696 | 0.06384 |  | 0.04347 | * | 0.18641 |  |
| DM2.bin.52 | 0 | 2676.25 | 470.05 | 0.06463 |  | 0.09245 |  | 0.085 |  |
| DM2.bin.54 | 0 | 4074.86 | 2335.72 | 0.16345 |  | 0.00827 | ** | 0.45439 |  |
| DM2.bin.61 | 0 | 368.52 | 252.413 | 0.07726 |  | 0.30406 |  | 0.62238 |  |
| DM2.bin.66 | 0 | 1438.3 | 885.411 | 0.10205 |  | 0.14934 |  | 0.43323 |  |
| DM2.bin.72 | 0 | 18984.4 | 4885.58 | 0.27746 |  | 0.42265 |  | 0.39172 |  |
| DM2.bin.81 | 0 | 3995.13 | 782.703 | 0.18364 |  | 0.23904 |  | 0.24589 |  |
| DM2.bin.83 | 0 | 1041.88 | 0 | 0.18398 |  |  |  | 0.18398 |  |
| DM2.bin.84 | 0 | 34336.3 | 957.575 | 0.18976 |  | 0.25917 |  | 0.19761 |  |
| DM2.bin.94 | 0 | 1286.6 | 388.406 | 0.21789 |  | 0.01837 | * | 0.34085 |  |
| DM2.bin.95 | 0 | 338.966 | 92.0093 | 0.20557 |  | 0.18637 |  | 0.30869 |  |
| DM3.bin.1 | 0 | 1032.55 | 107.306 | 0.28455 |  | 0.42265 |  | 0.32325 |  |
| DM3.bin.100 | 0 | 2777.75 | 2113.08 | 0.00586 | ** | 0.42265 |  | 0.78341 |  |
| DM3.bin.101 | 0 | 1255.05 | 844.619 | 0.05614 |  | 0.03045 | * | 0.32294 |  |
| DM3.bin.102 | 0 | 4389.03 | 3185.19 | 0.1528 |  | 0.18541 |  | 0.65888 |  |
| DM3.bin.104 | 0 | 854.119 | 215.931 | 0.42265 |  | 0.42265 |  | 0.53659 |  |
| DM3.bin.106 | 0 | 1693.39 | 121.463 | 0.39768 |  | 0.42265 |  | 0.42639 |  |
| DM3.bin.110 | 0 | 7220.5 | 422.821 | 0.38708 |  | 0.19097 |  | 0.41025 |  |
| DM3.bin.111 | 0 | 1734.24 | 449.077 | 0.25068 |  | 0.28149 |  | 0.35796 |  |
| DM3.bin.113 | 0 | 3244.63 | 0 | 0.42265 |  |  |  | 0.42265 |  |
| DM3.bin.118 | 0 | 2903.66 | 2176.94 | 0.07608 |  | 0.07563 |  | 0.53397 |  |
| DM3.bin.12 | 0 | 2827.48 | 1510.72 | 0.33581 |  | 0.1804 |  | 0.62549 |  |
| DM3.bin.122 | 0 | 5538.14 | 0 | 0.32833 |  |  |  | 0.32833 |  |
| DM3.bin.124 | 0 | 801.852 | 73.2938 | 0.42265 |  | 0.42265 |  | 0.45971 |  |
| DM3.bin.128 | 0 | 3682.5 | 729.645 | 0.42265 |  | 0.33174 |  | 0.50789 |  |
| DM3.bin.129 | 0 | 932.946 | 564.731 | 0.17465 |  | 0.01057 | * | 0.50099 |  |
| DM3.bin.20 | 0 | 5611.96 | 9125.62 | 0.20225 |  | 0.18638 |  | 0.56305 |  |
| DM3.bin.21 | 0 | 2339.29 | 925.838 | 0.17013 |  | 0.42265 |  | 0.38569 |  |
| DM3.bin.23 | 0 | 1871.46 | 91.8149 | 0.3079 |  | 0.42265 |  | 0.32608 |  |
| DM3.bin.26 | 0 | 1572.16 | 649.272 | 0.42265 |  | 0.13744 |  | 0.61848 |  |
| DM3.bin.31 | 0 | 3009.42 | 1258.62 | 0.25908 |  | 0.00476 | ** | 0.45991 |  |
| DM3.bin.33 | 410.301 | 6063.09 | 2475.31 | 0.20124 |  | 0.08926 |  | 0.35545 |  |
| DM3.bin.35 | 0 | 2552.06 | 380.954 | 0.16645 |  | 0.20243 |  | 0.20847 |  |
| DM3.bin.36 | 0 | 2452.14 | 1059.01 | 0.01698 | * | 0.05064 |  | 0.02977 | * |
| DM3.bin.37 | 0 | 5069.4 | 864.346 | 0.30536 |  | 0.00882 | ** | 0.37483 |  |
| DM3.bin.38 | 417.419 | 2288.87 | 1338.95 | 0.32417 |  | 0.03786 | * | 0.579 |  |
| DM3.bin.43 | 0 | 460.379 | 422.269 | 0.42265 |  | 0.11024 |  | 0.94338 |  |
| DM3.bin.44 | 0 | 441.239 | 306.617 | 0.13116 |  | 0.31362 |  | 0.66857 |  |
| DM3.bin.46 | 0 | 5967.5 | 10069.4 | 0.01132 | * | 0.32564 |  | 0.65173 |  |
| DM3.bin.49 | 0 | 3203.44 | 198.142 | 0.42265 |  | 0.19519 |  | 0.44723 |  |
| DM3.bin.5 | 0 | 1318.31 | 222.702 | 0.35346 |  | 0.24478 |  | 0.42443 |  |
| DM3.bin.53 | 0 | 1464.08 | 376.981 | 0.29428 |  | 0.06006 |  | 0.40543 |  |
| DM3.bin.55 | 0 | 1347.23 | 272.289 | 0.26454 |  | 0.14302 |  | 0.34501 |  |
| DM3.bin.56 | 0 | 1520.78 | 449.438 | 0.42265 |  | 0.42265 |  | 0.55979 |  |
| DM3.bin.57 | 0 | 8234.67 | 966.593 | 0.36619 |  | 0.04808 | * | 0.41398 |  |
| DM3.bin.58 | 0 | 1543.63 | 0 | 0.42265 |  |  |  | 0.42265 |  |
| DM3.bin.60 | 357.018 | 2819.42 | 1704.62 | 0.16657 |  | 0.08015 |  | 0.44183 |  |
| DM3.bin.61 | 0 | 3620.62 | 1784.04 | 0.38664 |  | 0.30909 |  | 0.64539 |  |
| DM3.bin.63 | 0 | 4257.94 | 1534.19 | 0.0324 | * | 0.00984 | ** | 0.06907 |  |
| DM3.bin.67 | 474.312 | 1942.8 | 644.222 | 0.36265 |  | 0.51834 |  | 0.41011 |  |
| DM3.bin.68 | 0 | 2778.9 | 1775.09 | 0.07264 |  | 0.20583 |  | 0.46694 |  |
| DM3.bin.69 | 0 | 3715.33 | 515.841 | 0.36878 |  | 0.19163 |  | 0.42618 |  |
| DM3.bin.7 | 0 | 4692.53 | 550.199 | 0.1612 |  | 0.19809 |  | 0.19244 |  |
| DM3.bin.70 | 0 | 14079.1 | 866.642 | 0.33955 |  | 0.14039 |  | 0.3634 |  |
| DM3.bin.75 | 0 | 1379.28 | 174.097 | 0.39733 |  | 0.42265 |  | 0.44971 |  |
| DM3.bin.76 | 233.846 | 7375.38 | 326.201 | 0.09999 |  | 0.63269 |  | 0.10157 |  |
| DM3.bin.78 | 0 | 973.737 | 435.092 | 0.3194 |  | 0.09663 |  | 0.54513 |  |
| DM3.bin.8 | 0 | 4034.36 | 1285.75 | 0.42265 |  | 0.22213 |  | 0.56794 |  |
| DM3.bin.85 | 0 | 387.416 | 164.045 | 0.33166 |  | 0.19991 |  | 0.54508 |  |
| DM3.bin.87 | 0 | 259.316 | 240.928 | 0.18626 |  | 0.09656 |  | 0.91175 |  |
| DM3.bin.88 | 0 | 843.677 | 105.758 | 0.42265 |  | 0.42265 |  | 0.47455 |  |
| DM3.bin.89 | 0 | 1616.08 | 0 | 0.42265 |  |  |  | 0.42265 |  |
| DM3.bin.90 | 0 | 927.18 | 67.4653 | 0.42265 |  | 0.42265 |  | 0.45182 |  |
| DM3.bin.91 | 0 | 5617.11 | 414.283 | 0.42265 |  | 0.19149 |  | 0.45214 |  |
| grpDL.bin.1 | 11169.3 | 573.93 | 576.23 | 0.00562 | ** | 0.00571 | ** | 0.97949 |  |
| grpDL.bin.12 | 2325.88 | 2097.13 | 2124.53 | 0.35854 |  | 0.50623 |  | 0.92608 |  |
| grpDL.bin.13 | 3663.22 | 424.214 | 436.067 | 0.00413 | ** | 0.00392 | ** | 0.85515 |  |
| grpDL.bin.16 | 39047.9 | 934.878 | 2896.85 | 9.9E-07 | *** | 0.00022 | *** | 0.24181 |  |
| grpDL.bin.21 | 1532.36 | 0 | 0 | 0.0001 | *** | 0.0001 | *** |  |  |
| grpDL.bin.24 | 18695.5 | 965.495 | 852.464 | 0.00051 | *** | 0.00056 | *** | 0.58736 |  |
| grpDL.bin.27 | 3784.45 | 0 | 0 | 0.0032 | ** | 0.0032 | ** |  |  |
| grpDL.bin.28 | 5117.05 | 0 | 196.984 | 0.01151 | * | 0.00688 | ** | 0.42265 |  |
| grpDL.bin.3 | 2049.68 | 0 | 0 | 8E-05 | *** | 8E-05 | *** |  |  |
| grpDL.bin.32 | 5303.77 | 1130.43 | 1202.56 | 0.00019 | *** | 0.00509 | ** | 0.87988 |  |
| grpDL.bin.37 | 1862.2 | 0 | 0 | 0.00047 | *** | 0.00047 | *** |  |  |
| grpDL.bin.4 | 2526.78 | 0 | 0 | 0.00812 | ** | 0.00812 | ** |  |  |
| grpDL.bin.44 | 1021.48 | 366.08 | 151.163 | 0.0131 | * | 0.00406 | ** | 0.15005 |  |
| grpDL.bin.47 | 6299.05 | 553.004 | 506.786 | 0.00265 | ** | 0.00285 | ** | 0.41058 |  |
| grpDL.bin.48 | 30745.6 | 341.679 | 356.086 | 0.00718 | ** | 0.00718 | ** | 0.72998 |  |
| grpDL.bin.49 | 2842 | 143.409 | 0 | 0.00062 | *** | 0.00273 | ** | 0.18359 |  |
| grpDL.bin.51 | 3638.82 | 0 | 0 | 0.00161 | ** | 0.00161 | ** |  |  |
| grpDL.bin.6 | 2852.9 | 87.6265 | 321.725 | 0.00831 | ** | 0.0048 | ** | 0.54705 |  |
| grpDL.bin.62 | 3101.04 | 0 | 0 | 0.0044 | ** | 0.0044 | ** |  |  |
| grpDL.bin.63 | 2389.7 | 0 | 0 | 0.01856 | * | 0.01856 | * |  |  |
| grpDL.bin.66 | 2362.69 | 0 | 0 | 0.01097 | * | 0.01097 | * |  |  |
| grpDL.bin.7 | 2830.59 | 380.419 | 218.252 | 0.00588 | ** | 0.00123 | ** | 0.28138 |  |
| grpDM.bin.106 | 0 | 893.176 | 50.7651 | 0.22367 |  | 0.42265 |  | 0.24135 |  |
| grpDM.bin.112 | 0 | 3776.85 | 2455.77 | 0.15611 |  | 0.42265 |  | 0.6837 |  |
| grpDM.bin.115 | 0 | 1817.49 | 450.5 | 0.13528 |  | 0.03327 | * | 0.20726 |  |
| grpDM.bin.116 | 0 | 722.754 | 376.731 | 0.18409 |  | 0.02223 | * | 0.44082 |  |
| grpDM.bin.119 | 0 | 881.7 | 363.679 | 0.01214 | * | 0.06356 |  | 0.01964 | * |
| grpDM.bin.123 | 0 | 4286.74 | 1277.95 | 0.00742 | ** | 0.23375 |  | 0.03961 | * |
| grpDM.bin.126 | 0 | 1313.03 | 0 | 0.42265 |  |  |  | 0.42265 |  |
| grpDM.bin.13 | 0 | 899.266 | 632.713 | 0.15485 |  | 0.06606 |  | 0.58941 |  |
| grpDM.bin.132 | 0 | 1548.75 | 1415.85 | 0.00987 | ** | 0.1507 |  | 0.85312 |  |
| grpDM.bin.133 | 0 | 1453.6 | 384.317 | 0.30954 |  | 0.00814 | ** | 0.42537 |  |
| grpDM.bin.138 | 0 | 1145.97 | 770.024 | 0.08862 |  | 0.19917 |  | 0.53041 |  |
| grpDM.bin.139 | 0 | 3048.75 | 574.767 | 0.32843 |  | 0.29535 |  | 0.40793 |  |
| grpDM.bin.14 | 0 | 1450.4 | 303.65 | 0.0056 | ** | 0.09339 |  | 0.00154 | ** |
| grpDM.bin.146 | 0 | 3675.99 | 152.927 | 0.40502 |  | 0.2846 |  | 0.42138 |  |
| grpDM.bin.149 | 0 | 1994.66 | 1307.43 | 0.10659 |  | 0.00803 | ** | 0.43546 |  |
| grpDM.bin.150 | 0 | 7249.81 | 2469.26 | 0.32481 |  | 0.2225 |  | 0.4863 |  |
| grpDM.bin.151 | 0 | 1497.65 | 1100.71 | 0.1354 |  | 0.07125 |  | 0.60566 |  |
| grpDM.bin.155 | 0 | 3122.71 | 2429.13 | 0.26032 |  | 0.14633 |  | 0.77945 |  |
| grpDM.bin.156 | 0 | 1587.27 | 456.795 | 0.14249 |  | 0.42265 |  | 0.24616 |  |
| grpDM.bin.157 | 0 | 2229.5 | 1940.04 | 0.0872 |  | 0.1135 |  | 0.78767 |  |
| grpDM.bin.158 | 0 | 1420.45 | 991.37 | 0.0169 | * | 0.08113 |  | 0.30457 |  |
| grpDM.bin.159 | 0 | 1109.07 | 162.083 | 0.1941 |  | 0.20566 |  | 0.2402 |  |
| grpDM.bin.16 | 0 | 1032.05 | 454.571 | 0.20244 |  | 0.42265 |  | 0.46616 |  |
| grpDM.bin.160 | 0 | 1626.7 | 952.072 | 0.07613 |  | 0.08449 |  | 0.30727 |  |
| grpDM.bin.164 | 0 | 1761.03 | 840.613 | 0.0082 | ** | 0.14837 |  | 0.11284 |  |
| grpDM.bin.169 | 0 | 702.563 | 524.338 | 0.19723 |  | 0.42265 |  | 0.79629 |  |
| grpDM.bin.173 | 0 | 311.43 | 177.767 | 0.00875 | ** | 0.28306 |  | 0.38909 |  |
| grpDM.bin.174 | 0 | 4763.47 | 0 | 0.18404 |  |  |  | 0.18404 |  |
| grpDM.bin.180 | 0 | 1536.12 | 350.85 | 0.04253 | * | 0.22731 |  | 0.04706 | * |
| grpDM.bin.20 | 0 | 1267.58 | 739.767 | 0.09158 |  | 0.03229 | * | 0.32912 |  |
| grpDM.bin.200 | 0 | 1696.32 | 873.592 | 0.08256 |  | 0.02099 | * | 0.25121 |  |
| grpDM.bin.201 | 0 | 565.539 | 499.403 | 0.00431 | ** | 0.08712 |  | 0.71952 |  |
| grpDM.bin.202 | 0 | 1251.09 | 418.404 | 0.11284 |  | 0.42265 |  | 0.25223 |  |
| grpDM.bin.203 | 0 | 1175.51 | 938.483 | 0.01486 | * | 0.06323 |  | 0.46579 |  |
| grpDM.bin.206 | 0 | 1320.88 | 133.993 | 0.03852 | * | 0.42265 |  | 0.02946 | * |
| grpDM.bin.207 | 0 | 1519.13 | 548.648 | 0.06997 |  | 0.00699 | ** | 0.14799 |  |
| grpDM.bin.215 | 0 | 1170.65 | 417.784 | 0.1312 |  | 0.03816 | * | 0.24947 |  |
| grpDM.bin.216 | 0 | 1725.14 | 830.53 | 0.03578 | * | 0.06253 |  | 0.10004 |  |
| grpDM.bin.218 | 0 | 4572.11 | 2435.05 | 0.1127 |  | 0.02264 | * | 0.33055 |  |
| grpDM.bin.22 | 0 | 1457.65 | 467.914 | 0.0975 |  | 0.02043 | * | 0.17973 |  |
| grpDM.bin.222 | 0 | 1539.93 | 168.021 | 0.13849 |  | 0.42265 |  | 0.15903 |  |
| grpDM.bin.225 | 0 | 1942.36 | 533.292 | 0.30466 |  | 0.05459 |  | 0.42564 |  |
| grpDM.bin.226 | 0 | 2181.51 | 120.692 | 0.2659 |  | 0.42265 |  | 0.28509 |  |
| grpDM.bin.23 | 0 | 457.222 | 508.432 | 0.26871 |  | 0.07205 |  | 0.88839 |  |
| grpDM.bin.230 | 0 | 1270.3 | 937.291 | 0.18719 |  | 0.18353 |  | 0.69919 |  |
| grpDM.bin.232 | 0 | 6035.49 | 144.258 | 0.42265 |  | 0.42265 |  | 0.43197 |  |
| grpDM.bin.233 | 0 | 1524.23 | 625.097 | 0.13169 |  | 0.00493 | ** | 0.28133 |  |
| grpDM.bin.236 | 0 | 1234.91 | 712.238 | 0.42265 |  | 0.42265 |  | 0.73682 |  |
| grpDM.bin.244 | 0 | 3870.06 | 899.952 | 0.34737 |  | 0.21246 |  | 0.44925 |  |
| grpDM.bin.253 | 0 | 980.642 | 376.85 | 0.1927 |  | 0.03107 | * | 0.35555 |  |
| grpDM.bin.256 | 0 | 1433.81 | 2081.86 | 0.23846 |  | 0.42265 |  | 0.79455 |  |
| grpDM.bin.258 | 0 | 1478.47 | 252.024 | 0.18442 |  | 0.18598 |  | 0.23785 |  |
| grpDM.bin.261 | 0 | 2890.03 | 179.607 | 0.25759 |  | 0.42265 |  | 0.27884 |  |
| grpDM.bin.264 | 235.855 | 855.174 | 128.451 | 0.43008 |  | 0.23605 |  | 0.36867 |  |
| grpDM.bin.269 | 0 | 3447.34 | 2627.83 | 0.18667 |  | 0.10878 |  | 0.70657 |  |
| grpDM.bin.27 | 0 | 4381.84 | 1053.86 | 0.21209 |  | 0.18441 |  | 0.30158 |  |
| grpDM.bin.274 | 0 | 1568.27 | 173.704 | 0.2363 |  | 0.18351 |  | 0.27469 |  |
| grpDM.bin.283 | 0 | 1433.27 | 2383.75 | 0.04552 | * | 0.10065 |  | 0.36979 |  |
| grpDM.bin.284 | 341.225 | 1682.43 | 652.756 | 0.09855 |  | 0.17149 |  | 0.14231 |  |
| grpDM.bin.285 | 0 | 3467.32 | 740.309 | 0.20604 |  | 0.35119 |  | 0.28097 |  |
| grpDM.bin.29 | 150.976 | 2098.91 | 1308.29 | 0.01011 | * | 4.5E-06 | *** | 0.05682 |  |
| grpDM.bin.294 | 0 | 1994.85 | 1398.88 | 0.06118 |  | 0.3246 |  | 0.65433 |  |
| grpDM.bin.295 | 0 | 2312.45 | 2724.9 | 0.12815 |  | 0.15535 |  | 0.80155 |  |
| grpDM.bin.296 | 0 | 1388.91 | 512.505 | 0.18585 |  | 0.42265 |  | 0.37455 |  |
| grpDM.bin.301 | 0 | 2081.53 | 1032.53 | 0.04823 | * | 0.03265 | * | 0.14529 |  |
| grpDM.bin.308 | 0 | 3421.11 | 806.889 | 0.06173 |  | 0.00509 | ** | 0.09872 |  |
| grpDM.bin.31 | 0 | 3556.24 | 123.156 | 0.13398 |  | 0.42265 |  | 0.1408 |  |
| grpDM.bin.310 | 0 | 10733.6 | 914.733 | 0.20594 |  | 0.2413 |  | 0.2323 |  |
| grpDM.bin.314 | 0 | 497.722 | 92.6777 | 0.10017 |  | 0.42265 |  | 0.12574 |  |
| grpDM.bin.318 | 0 | 4433.63 | 1679.88 | 0.01102 | * | 0.33214 |  | 0.16307 |  |
| grpDM.bin.32 | 0 | 1443.85 | 600.346 | 0.06443 |  | 0.0605 |  | 0.14798 |  |
| grpDM.bin.33 | 0 | 1443.91 | 575.258 | 0.09306 |  | 0.12688 |  | 0.20115 |  |
| grpDM.bin.34 | 0 | 833.334 | 557.109 | 0.08616 |  | 0.22215 |  | 0.54071 |  |
| grpDM.bin.37 | 0 | 255.043 | 101.876 | 0.07289 |  | 0.19718 |  | 0.17194 |  |
| grpDM.bin.38 | 0 | 420.113 | 329.213 | 0.02705 | * | 0.32382 |  | 0.75887 |  |
| grpDM.bin.39 | 0 | 1497.12 | 590.565 | 0.08668 |  | 0.09487 |  | 0.18544 |  |
| grpDM.bin.4 | 0 | 2542.29 | 0 | 0.22733 |  |  |  | 0.22733 |  |
| grpDM.bin.42 | 0 | 959.142 | 298.898 | 0.21133 |  | 0.20025 |  | 0.33806 |  |
| grpDM.bin.44 | 0 | 1148.01 | 1901.54 | 0.00419 | ** | 0.14977 |  | 0.46115 |  |
| grpDM.bin.48 | 0 | 2140.08 | 581.692 | 0.10592 |  | 0.0724 |  | 0.17118 |  |
| grpDM.bin.5 | 0 | 2041.8 | 1286.09 | 0.00334 | ** | 0.10948 |  | 0.24152 |  |
| grpDM.bin.52 | 0 | 7888.3 | 1106.89 | 0.14674 |  | 0.07026 |  | 0.18402 |  |
| grpDM.bin.6 | 0 | 2854.91 | 7177.69 | 0.2498 |  | 0.00712 | ** | 0.12422 |  |
| grpDM.bin.64 | 0 | 321.982 | 111.53 | 0.18372 |  | 0.20328 |  | 0.32208 |  |
| grpDM.bin.67 | 0 | 587.455 | 316.834 | 0.0351 | * | 0.02596 | * | 0.12398 |  |
| grpDM.bin.7 | 0 | 1865.73 | 463.759 | 0.31424 |  | 0.30285 |  | 0.42376 |  |
| grpDM.bin.71 | 0 | 4534.01 | 1489.27 | 0.12558 |  | 0.18426 |  | 0.22307 |  |
| grpDM.bin.72 | 0 | 3809.18 | 663.523 | 0.01676 | * | 0.20501 |  | 0.00894 | ** |
| grpDM.bin.87 | 0 | 1470.09 | 589.095 | 0.00801 | ** | 0.29034 |  | 0.1577 |  |
| grpDM.bin.89 | 0 | 533.925 | 149.957 | 0.18353 |  | 0.42265 |  | 0.29493 |  |
| grpDM.bin.92 | 0 | 416.67 | 136.06 | 0.22814 |  | 0.42265 |  | 0.38439 |  |
| grpDM.bin.93 | 0 | 1555.9 | 1072.45 | 0.02922 | * | 0.02113 | * | 0.21608 |  |
| grpDM.bin.94 | 0 | 1091.24 | 0 | 0.35937 |  |  |  | 0.35937 |  |
| grpDM.bin.96 | 53.9016 | 2010.51 | 276.735 | 0.01778 | * | 0.0519 |  | 0.0221 | * |
| grpH.bin.10 | 0 | 89.8248 | 2098.84 | 0.42265 |  | 0.1145 |  | 0.12126 |  |
| grpH.bin.100 | 0 | 391.409 | 3230.41 | 0.01802 | * | 0.38423 |  | 0.43386 |  |
| grpH.bin.102 | 0 | 293.105 | 912.464 | 0.11842 |  | 0.42265 |  | 0.56812 |  |
| grpH.bin.122 | 0 | 0 | 9382.61 |  |  | 0.41743 |  | 0.41743 |  |
| grpH.bin.126 | 0 | 943.866 | 2596.62 | 0.19737 |  | 0.36858 |  | 0.54261 |  |
| grpH.bin.129 | 0 | 0 | 2941.58 |  |  | 0.28688 |  | 0.28688 |  |
| grpH.bin.132 | 0 | 560.216 | 1401.37 | 0.01076 | * | 0.12098 |  | 0.25721 |  |
| grpH.bin.135 | 0 | 0 | 1324.28 |  |  | 0.35496 |  | 0.35496 |  |
| grpH.bin.136 | 0 | 11835.1 | 9386.5 | 0.18101 |  | 0.22822 |  | 0.77537 |  |
| grpH.bin.143 | 0 | 0 | 1439.7 |  |  | 0.3022 |  | 0.3022 |  |
| grpH.bin.152 | 0 | 1169.8 | 1807.67 | 0.05489 |  | 0.18423 |  | 0.56105 |  |
| grpH.bin.156 | 403.877 | 226.918 | 2929.1 | 0.25775 |  | 0.47949 |  | 0.45368 |  |
| grpH.bin.160 | 0 | 2285.16 | 3956.42 | 0.06543 |  | 0.10667 |  | 0.36297 |  |
| grpH.bin.171 | 0 | 101.179 | 228.517 | 0.23818 |  | 0.12294 |  | 0.30909 |  |
| grpH.bin.175 | 1323.72 | 8687.55 | 17931.2 | 0.09818 |  | 0.25179 |  | 0.47081 |  |
| grpH.bin.18 | 0 | 2842.72 | 4928.9 | 0.1268 |  | 0.20222 |  | 0.52421 |  |
| grpH.bin.181 | 0 | 1207.61 | 1391.35 | 0.07636 |  | 0.13297 |  | 0.79911 |  |
| grpH.bin.185 | 367.484 | 335.905 | 2303.73 | 0.93368 |  | 0.27475 |  | 0.26625 |  |
| grpH.bin.190 | 0 | 263.554 | 2184.56 | 0.18472 |  | 0.33486 |  | 0.38335 |  |
| grpH.bin.192 | 0 | 3964.69 | 5404.24 | 0.03583 | * | 0.20609 |  | 0.67613 |  |
| grpH.bin.194 | 0 | 233.364 | 1861.6 | 0.19178 |  | 0.27564 |  | 0.32305 |  |
| grpH.bin.196 | 0 | 0 | 1075.62 |  |  | 0.42265 |  | 0.42265 |  |
| grpH.bin.20 | 0 | 38175.8 | 5631.23 | 0.18834 |  | 0.20894 |  | 0.2339 |  |
| grpH.bin.202 | 0 | 323.22 | 549.59 | 0.03479 | * | 0.03456 | * | 0.1532 |  |
| grpH.bin.21 | 284.741 | 1468.33 | 2634.17 | 0.33363 |  | 0.4387 |  | 0.69132 |  |
| grpH.bin.210 | 0 | 190.556 | 968.537 | 0.18582 |  | 0.36526 |  | 0.44962 |  |
| grpH.bin.212 | 0 | 422.857 | 5719.4 | 0.18987 |  | 0.27372 |  | 0.30048 |  |
| grpH.bin.218 | 0 | 2055.24 | 1396.27 | 0.07792 |  | 0.0832 |  | 0.43234 |  |
| grpH.bin.225 | 0 | 140.497 | 439.463 | 0.03687 | * | 0.27176 |  | 0.41425 |  |
| grpH.bin.229 | 0 | 166.564 | 1468.19 | 0.18416 |  | 0.27957 |  | 0.32233 |  |
| grpH.bin.23 | 0 | 573.536 | 834.456 | 0.27733 |  | 0.42265 |  | 0.79625 |  |
| grpH.bin.236 | 0 | 97.7159 | 288.372 | 0.19204 |  | 0.19332 |  | 0.33027 |  |
| grpH.bin.237 | 0 | 492.332 | 1567.24 | 0.18812 |  | 0.20636 |  | 0.3329 |  |
| grpH.bin.238 | 0 | 56.7007 | 1016.83 | 0.42265 |  | 0.20491 |  | 0.22112 |  |
| grpH.bin.243 | 0 | 740.502 | 2327.04 | 0.42265 |  | 0.40364 |  | 0.55565 |  |
| grpH.bin.245 | 0 | 484.656 | 1146.58 | 0.09205 |  | 0.09691 |  | 0.22194 |  |
| grpH.bin.249 | 0 | 482.749 | 877.072 | 0.03232 | * | 0.01896 | * | 0.06554 |  |
| grpH.bin.256 | 0 | 660.472 | 1772.95 | 0.0224 | * | 0.08828 |  | 0.18444 |  |
| grpH.bin.266 | 0 | 420.683 | 1281.95 | 0.01648 | * | 0.19878 |  | 0.33101 |  |
| grpH.bin.271 | 0 | 69.3536 | 5280.12 | 0.42265 |  | 0.42265 |  | 0.42774 |  |
| grpH.bin.275 | 0 | 276.661 | 1055.72 | 0.02332 | * | 0.24093 |  | 0.34767 |  |
| grpH.bin.276 | 205.376 | 3023.65 | 3787.72 | 0.15175 |  | 0.24036 |  | 0.77879 |  |
| grpH.bin.28 | 0 | 0 | 775.959 |  |  | 0.42265 |  | 0.42265 |  |
| grpH.bin.281 | 0 | 555.531 | 1085.14 | 0.00211 | ** | 0.14724 |  | 0.37678 |  |
| grpH.bin.288 | 0 | 768.981 | 1278.51 | 0.27202 |  | 0.15891 |  | 0.54722 |  |
| grpH.bin.294 | 0 | 1196.91 | 5455.13 | 0.03915 | * | 0.25862 |  | 0.34682 |  |
| grpH.bin.298 | 0 | 581.687 | 4007.51 | 0.1954 |  | 0.40516 |  | 0.46559 |  |
| grpH.bin.302 | 0 | 123.274 | 387.537 | 0.42265 |  | 0.09614 |  | 0.21396 |  |
| grpH.bin.31 | 0 | 83.0132 | 1354.08 | 0.42265 |  | 0.42265 |  | 0.44707 |  |
| grpH.bin.310 | 0 | 443.614 | 704.536 | 0.05605 |  | 0.05918 |  | 0.29542 |  |
| grpH.bin.319 | 0 | 540.301 | 1299.08 | 0.0358 | * | 0.26807 |  | 0.46903 |  |
| grpH.bin.32 | 0 | 0 | 1185 |  |  | 0.42265 |  | 0.42265 |  |
| grpH.bin.323 | 0 | 0 | 535.806 |  |  | 0.42265 |  | 0.42265 |  |
| grpH.bin.324 | 0 | 561.085 | 4974.35 | 0.01044 | * | 0.37824 |  | 0.42417 |  |
| grpH.bin.36 | 0 | 463.931 | 1630.82 | 0.00054 | *** | 0.14702 |  | 0.23998 |  |
| grpH.bin.39 | 0 | 1454.65 | 1418.94 | 0.0674 |  | 0.06541 |  | 0.95148 |  |
| grpH.bin.42 | 0 | 501.096 | 2803.76 | 0.20398 |  | 0.12053 |  | 0.15844 |  |
| grpH.bin.46 | 0 | 649.286 | 1021.84 | 0.11237 |  | 0.20967 |  | 0.58813 |  |
| grpH.bin.50 | 0 | 932.494 | 1574.23 | 0.24666 |  | 0.27414 |  | 0.62932 |  |
| grpH.bin.53 | 0 | 0 | 1484.29 |  |  | 0.42265 |  | 0.42265 |  |
| grpH.bin.57 | 0 | 862.71 | 8518.63 | 0.13362 |  | 0.40411 |  | 0.44522 |  |
| grpH.bin.65 | 0 | 766.673 | 2016.07 | 0.02485 | * | 0.31812 |  | 0.50003 |  |
| grpH.bin.66 | 0 | 0 | 2339.62 |  |  | 0.42265 |  | 0.42265 |  |
| grpH.bin.71 | 0 | 0 | 7759.25 |  |  | 0.15247 |  | 0.15247 |  |
| grpH.bin.74 | 0 | 1870.83 | 3098 | 0.00648 | ** | 0.16474 |  | 0.48483 |  |
| grpH.bin.80 | 0 | 86.7111 | 158.271 | 0.08058 |  | 0.02324 | * | 0.11754 |  |
| grpH.bin.81 | 0 | 361.131 | 1476.84 | 0.00207 | ** | 0.13124 |  | 0.20167 |  |
| grpH.bin.84 | 208.173 | 0 | 803.777 | 0.00442 | ** | 0.38251 |  | 0.2728 |  |
| grpH.bin.95 | 0 | 899.367 | 1192.93 | 0.18424 |  | 0.01142 | * | 0.58725 |  |
| grpH.bin.98 | 28.8354 | 1657.3 | 1452.48 | 0.11671 |  | 0.10613 |  | 0.80968 |  |
| grpH.bin.99 | 0 | 777.361 | 9005.8 | 0.00599 | ** | 0.35836 |  | 0.39273 |  |
| H1.bin.10 | 0 | 477.454 | 1332.4 | 0.02591 | * | 0.35023 |  | 0.51932 |  |
| H1.bin.101 | 0 | 971.791 | 1643.87 | 0.42265 |  | 0.32028 |  | 0.69495 |  |
| H1.bin.103 | 0 | 1657.83 | 3887.79 | 0.03197 | * | 0.10227 |  | 0.23677 |  |
| H1.bin.108 | 0 | 184.593 | 3687.46 | 0.01191 | * | 0.1767 |  | 0.19067 |  |
| H1.bin.109 | 0 | 538.858 | 1780.81 | 0.0218 | * | 0.24691 |  | 0.37608 |  |
| H1.bin.113 | 3713.24 | 202.096 | 1641.2 | 0.00021 | *** | 0.31487 |  | 0.45323 |  |
| H1.bin.114 | 0 | 1149.17 | 5527.38 | 0.05636 |  | 0.22508 |  | 0.30296 |  |
| H1.bin.117 | 0 | 1605.31 | 5480.81 | 0.25462 |  | 0.33137 |  | 0.46571 |  |
| H1.bin.120 | 0 | 4058.03 | 2943.77 | 0.04344 | * | 0.11096 |  | 0.46743 |  |
| H1.bin.125 | 0 | 0 | 1373.61 |  |  | 0.34129 |  | 0.34129 |  |
| H1.bin.13 | 0 | 870.549 | 3555.52 | 0.07666 |  | 0.42265 |  | 0.52922 |  |
| H1.bin.15 | 0 | 373.817 | 1416.37 | 0.096 |  | 0.30558 |  | 0.42112 |  |
| H1.bin.16 | 0 | 2061.15 | 4418.46 | 0.03874 | * | 0.21213 |  | 0.437 |  |
| H1.bin.17 | 0 | 2458.52 | 3193.66 | 0.00225 | ** | 0.09911 |  | 0.56955 |  |
| H1.bin.2 | 412.264 | 1620.84 | 4422.35 | 0.16268 |  | 0.2319 |  | 0.35768 |  |
| H1.bin.20 | 0 | 81.739 | 791.274 | 0.42265 |  | 0.42265 |  | 0.46484 |  |
| H1.bin.21 | 0 | 1459.98 | 2767.97 | 0.42265 |  | 0.34963 |  | 0.65903 |  |
| H1.bin.22 | 0 | 1093.46 | 5556.24 | 0.0143 | * | 0.23696 |  | 0.31186 |  |
| H1.bin.23 | 0 | 908.552 | 6612.7 | 0.19308 |  | 0.24149 |  | 0.29078 |  |
| H1.bin.24 | 0 | 262.425 | 965.674 | 0.19002 |  | 0.26916 |  | 0.38547 |  |
| H1.bin.25 | 0 | 1787.12 | 7702.87 | 0.00636 | ** | 0.32058 |  | 0.42048 |  |
| H1.bin.26 | 0 | 823.256 | 1616.07 | 0.02468 | * | 0.1614 |  | 0.39767 |  |
| H1.bin.29 | 0 | 0 | 2377.8 |  |  | 0.42265 |  | 0.42265 |  |
| H1.bin.35 | 0 | 766.305 | 3006.05 | 0.09061 |  | 0.20844 |  | 0.30467 |  |
| H1.bin.36 | 0 | 234.228 | 240.756 | 0.00106 | ** | 0.29422 |  | 0.97301 |  |
| H1.bin.38 | 0 | 904.736 | 2340.27 | 0.01882 | * | 0.14157 |  | 0.28281 |  |
| H1.bin.4 | 722.448 | 7132.11 | 6830.29 | 0.11295 |  | 0.07335 |  | 0.92353 |  |
| H1.bin.40 | 0 | 351.857 | 703.901 | 0.13015 |  | 0.42265 |  | 0.66918 |  |
| H1.bin.43 | 0 | 283.892 | 2830.76 | 0.42265 |  | 0.37686 |  | 0.41757 |  |
| H1.bin.44 | 0 | 274.716 | 1993.1 | 0.18418 |  | 0.3242 |  | 0.37983 |  |
| H1.bin.46 | 202.457 | 1561.75 | 1331.59 | 0.11771 |  | 0.20481 |  | 0.78725 |  |
| H1.bin.50 | 135.16 | 0 | 27867.7 | 0.42265 |  | 0.41387 |  | 0.412 |  |
| H1.bin.53 | 114.244 | 8793.02 | 29791.4 | 0.08917 |  | 0.16997 |  | 0.2729 |  |
| H1.bin.54 | 0 | 900.481 | 2646.59 | 0.00813 | ** | 0.24399 |  | 0.3939 |  |
| H1.bin.60 | 0 | 1356.3 | 2057.74 | 0.03459 | * | 0.09853 |  | 0.42716 |  |
| H1.bin.62 | 0 | 212.309 | 3666.84 | 0.42265 |  | 0.38401 |  | 0.40692 |  |
| H1.bin.64 | 0 | 415.943 | 299.028 | 0.03848 | * | 0.0226 | * | 0.3067 |  |
| H1.bin.7 | 0 | 303.067 | 1016.39 | 0.06403 |  | 0.1442 |  | 0.23984 |  |
| H1.bin.77 | 23.2486 | 0 | 13272.1 | 0.42265 |  | 0.42332 |  | 0.42265 |  |
| H1.bin.79 | 0 | 405.275 | 2527.99 | 0.18539 |  | 0.31468 |  | 0.37997 |  |
| H1.bin.8 | 0 | 1180.83 | 1372.25 | 0.06299 |  | 0.12565 |  | 0.77723 |  |
| H1.bin.80 | 0 | 3610.86 | 3258.74 | 0.02142 | * | 0.17988 |  | 0.85152 |  |
| H1.bin.81 | 3192.57 | 3988.13 | 5604.69 | 0.70451 |  | 0.50784 |  | 0.67499 |  |
| H1.bin.82 | 0 | 410.24 | 2241.73 | 0.00441 | ** | 0.29913 |  | 0.37398 |  |
| H1.bin.85 | 0 | 0 | 1006.64 |  |  | 0.2871 |  | 0.2871 |  |
| H1.bin.89 | 0 | 0 | 2178.03 |  |  | 0.27894 |  | 0.27894 |  |
| H1.bin.91 | 0 | 71.7836 | 2194.88 | 0.42265 |  | 0.3627 |  | 0.37531 |  |
| H1.bin.94 | 0 | 129.431 | 275.129 | 0.22522 |  | 0.09092 |  | 0.28048 |  |
| H1.bin.95 | 404.137 | 4611.58 | 5563.12 | 0.06958 |  | 0.12409 |  | 0.70826 |  |
| H1.bin.96 | 0 | 461.407 | 2566.07 | 0.01954 | * | 0.257 |  | 0.32669 |  |
| H1.bin.97 | 0 | 1288.89 | 1841.29 | 0.00577 | ** | 0.05213 |  | 0.33347 |  |
| H1.bin.98 | 0 | 1776.13 | 2582.64 | 6.6E-05 | *** | 0.12917 |  | 0.51595 |  |
| H2.bin.1 | 0 | 95.2825 | 244.029 | 0.18388 |  | 0.1066 |  | 0.227 |  |
| H2.bin.10 | 0 | 145.431 | 209.954 | 0.23977 |  | 0.28544 |  | 0.72724 |  |
| H2.bin.105 | 0 | 0 | 4759.34 |  |  | 0.39764 |  | 0.39764 |  |
| H2.bin.106 | 0 | 669.563 | 2958.72 | 0.26395 |  | 0.36479 |  | 0.46417 |  |
| H2.bin.109 | 546.882 | 3686.54 | 5701.07 | 0.03774 | * | 0.06579 |  | 0.28537 |  |
| H2.bin.11 | 0 | 4629.05 | 7225.01 | 0.08364 |  | 0.14653 |  | 0.50783 |  |
| H2.bin.113 | 0 | 6719.23 | 5479.3 | 0.02696 | * | 0.14687 |  | 0.67018 |  |
| H2.bin.115 | 0 | 622.228 | 2560.35 | 0.21083 |  | 0.21364 |  | 0.3046 |  |
| H2.bin.116 | 0 | 0 | 1296.11 |  |  | 0.34891 |  | 0.34891 |  |
| H2.bin.117 | 0 | 409.02 | 6887.45 | 0.0015 | ** | 0.39663 |  | 0.42016 |  |
| H2.bin.118 | 0 | 905.839 | 1980.91 | 0.18568 |  | 0.38803 |  | 0.61705 |  |
| H2.bin.12 | 0 | 238.613 | 3664.19 | 0.18924 |  | 0.33495 |  | 0.36015 |  |
| H2.bin.127 | 0 | 3984.09 | 16574.7 | 0.01022 | * | 0.15469 |  | 0.23103 |  |
| H2.bin.128 | 0 | 686.482 | 2068.11 | 0.08738 |  | 0.22902 |  | 0.37113 |  |
| H2.bin.13 | 0 | 0 | 1186.69 |  |  | 0.30997 |  | 0.30997 |  |
| H2.bin.132 | 349.857 | 11685.8 | 12698.9 | 0.09305 |  | 0.14261 |  | 0.88321 |  |
| H2.bin.133 | 0 | 1476.65 | 2930.56 | 0.04444 | * | 0.0521 |  | 0.16031 |  |
| H2.bin.18 | 0 | 2042.89 | 6531.72 | 0.01955 | * | 0.32862 |  | 0.47158 |  |
| H2.bin.19 | 0 | 0 | 636.204 |  |  | 0.42265 |  | 0.42265 |  |
| H2.bin.20 | 0 | 4734.17 | 5593.32 | 0.1719 |  | 0.30538 |  | 0.86566 |  |
| H2.bin.26 | 0 | 0 | 6747.33 |  |  | 0.42265 |  | 0.42265 |  |
| H2.bin.28 | 0 | 1640.99 | 1595.33 | 0.20578 |  | 0.25421 |  | 0.97453 |  |
| H2.bin.29 | 0 | 359.279 | 4612.36 | 0.1842 |  | 0.40859 |  | 0.43982 |  |
| H2.bin.30 | 0 | 305.353 | 1417.41 | 0.1904 |  | 0.26982 |  | 0.35725 |  |
| H2.bin.32 | 0 | 1351.25 | 2424.98 | 0.03756 | * | 0.05557 |  | 0.20663 |  |
| H2.bin.34 | 0 | 160.545 | 1053.94 | 0.0029 | ** | 0.22188 |  | 0.27581 |  |
| H2.bin.35 | 0 | 666.849 | 2352.93 | 0.42265 |  | 0.2471 |  | 0.37415 |  |
| H2.bin.36 | 709.578 | 11193.7 | 8263.81 | 0.0829 |  | 0.10851 |  | 0.52617 |  |
| H2.bin.4 | 0 | 1876.95 | 2373.45 | 0.13385 |  | 0.19366 |  | 0.75241 |  |
| H2.bin.40 | 0 | 0 | 3492.02 |  |  | 0.42265 |  | 0.42265 |  |
| H2.bin.43 | 0 | 1620.29 | 2643.2 | 0.02282 | * | 0.28257 |  | 0.63094 |  |
| H2.bin.44 | 0 | 0 | 1101.22 |  |  | 0.42265 |  | 0.42265 |  |
| H2.bin.46 | 0 | 123.002 | 1279.57 | 0.42265 |  | 0.37064 |  | 0.40945 |  |
| H2.bin.48 | 0 | 273.683 | 2378.64 | 0.18351 |  | 0.29107 |  | 0.33519 |  |
| H2.bin.53 | 0 | 166.389 | 1592.06 | 0.18356 |  | 0.42265 |  | 0.4651 |  |
| H2.bin.56 | 0 | 507.647 | 2969.86 | 0.01411 | * | 0.33464 |  | 0.40571 |  |
| H2.bin.57 | 0 | 202.18 | 1271.86 | 0.19036 |  | 0.2599 |  | 0.3202 |  |
| H2.bin.59 | 0 | 2961.36 | 3516.9 | 0.03824 | * | 0.05328 |  | 0.62292 |  |
| H2.bin.6 | 0 | 0 | 1949.81 |  |  | 0.42265 |  | 0.42265 |  |
| H2.bin.7 | 0 | 0 | 920.168 |  |  | 0.42265 |  | 0.42265 |  |
| H2.bin.71 | 0 | 325.045 | 447.512 | 0.0044 | ** | 0.19729 |  | 0.65488 |  |
| H2.bin.73 | 0 | 3019.49 | 6746.24 | 0.09044 |  | 0.1962 |  | 0.40368 |  |
| H2.bin.75 | 0 | 961.631 | 2889.8 | 0.09938 |  | 0.32248 |  | 0.47718 |  |
| H2.bin.76 | 0 | 2256.69 | 6536.68 | 0.02902 | * | 0.20018 |  | 0.34229 |  |
| H2.bin.79 | 0 | 1348.25 | 1734.61 | 0.0219 | * | 0.07468 |  | 0.53385 |  |
| H2.bin.8 | 0 | 2781.83 | 6435.03 | 0.07764 |  | 0.3741 |  | 0.58666 |  |
| H2.bin.80 | 0 | 96.5062 | 2201.75 | 0.42265 |  | 0.42265 |  | 0.43993 |  |
| H2.bin.81 | 0 | 363.752 | 2061.36 | 0.18454 |  | 0.38219 |  | 0.45703 |  |
| H2.bin.84 | 0 | 2201.88 | 4055.11 | 0.00509 | ** | 0.23479 |  | 0.52287 |  |
| H2.bin.86 | 0 | 0 | 1183.15 |  |  | 0.42265 |  | 0.42265 |  |
| H2.bin.87 | 356.486 | 2147.36 | 2097.54 | 0.00353 | ** | 0.15615 |  | 0.95529 |  |
| H2.bin.89 | 0 | 1877.65 | 6273.87 | 0.00465 | ** | 0.2714 |  | 0.40237 |  |
| H2.bin.90 | 450.194 | 8056.05 | 6247.84 | 0.12916 |  | 0.25131 |  | 0.72239 |  |
| H2.bin.95 | 0 | 3518.81 | 3872.69 | 0.04277 | * | 0.19746 |  | 0.88257 |  |
| H3.bin.102 | 0 | 2911.74 | 996.759 | 0.42265 |  | 0.42265 |  | 0.58657 |  |
| H3.bin.104 | 0 | 79.5575 | 2352.38 | 0.42265 |  | 0.42265 |  | 0.43591 |  |
| H3.bin.107 | 0 | 925.521 | 2027.39 | 0.02469 | * | 0.23627 |  | 0.45961 |  |
| H3.bin.117 | 0 | 487.494 | 3276.57 | 0.0354 | * | 0.38251 |  | 0.44442 |  |
| H3.bin.119 | 0 | 380.252 | 2005.79 | 0.18493 |  | 0.29153 |  | 0.36879 |  |
| H3.bin.121 | 0 | 289.559 | 800.367 | 0.02132 | * | 0.30137 |  | 0.47129 |  |
| H3.bin.125 | 0 | 544.089 | 1364.11 | 0.21122 |  | 0.32745 |  | 0.52526 |  |
| H3.bin.127 | 0 | 1169.96 | 1522.56 | 0.00401 | ** | 0.16493 |  | 0.66904 |  |
| H3.bin.128 | 0 | 659.953 | 1599.47 | 0.01409 | * | 0.21646 |  | 0.40484 |  |
| H3.bin.130 | 0 | 667.766 | 2136.77 | 0.03446 | * | 0.25639 |  | 0.39261 |  |
| H3.bin.134 | 0 | 2052.1 | 3451.59 | 0.02666 | * | 0.27361 |  | 0.60745 |  |
| H3.bin.135 | 0 | 372.156 | 2116.65 | 0.00254 | ** | 0.36066 |  | 0.43471 |  |
| H3.bin.14 | 0 | 818.925 | 1598.06 | 0.21358 |  | 0.3137 |  | 0.5926 |  |
| H3.bin.150 | 0 | 0 | 912.976 |  |  | 0.29377 |  | 0.29377 |  |
| H3.bin.154 | 0 | 92.3933 | 1393.56 | 0.18377 |  | 0.22115 |  | 0.24246 |  |
| H3.bin.156 | 0 | 407.792 | 1260.42 | 0.00352 | ** | 0.1389 |  | 0.24644 |  |
| H3.bin.157 | 0 | 748.758 | 1873.63 | 0.18474 |  | 0.19613 |  | 0.37399 |  |
| H3.bin.158 | 0 | 2772.05 | 2731.63 | 0.08853 |  | 0.26566 |  | 0.98512 |  |
| H3.bin.161 | 0 | 1218.55 | 1738.65 | 0.02 | * | 0.23928 |  | 0.67091 |  |
| H3.bin.162 | 0 | 722.349 | 3309.51 | 0.1929 |  | 0.25198 |  | 0.33836 |  |
| H3.bin.163 | 0 | 966.154 | 2209.84 | 0.00862 | ** | 0.31156 |  | 0.52895 |  |
| H3.bin.166 | 2947.48 | 8315.01 | 11551.9 | 0.04587 | * | 0.23975 |  | 0.60047 |  |
| H3.bin.168 | 507.555 | 1800.14 | 4436.93 | 0.07391 |  | 0.24491 |  | 0.3886 |  |
| H3.bin.172 | 322.529 | 8428.64 | 8811.89 | 0.01253 | * | 0.17853 |  | 0.93597 |  |
| H3.bin.19 | 0 | 2031.69 | 2607.7 | 0.01107 | * | 0.10629 |  | 0.60076 |  |
| H3.bin.21 | 0 | 318.964 | 525.544 | 0.01876 | * | 0.05389 |  | 0.24095 |  |
| H3.bin.23 | 0 | 287.231 | 1230.59 | 0.02592 | * | 0.22695 |  | 0.31717 |  |
| H3.bin.26 | 0 | 633.726 | 1364.65 | 0.07014 |  | 0.38984 |  | 0.61992 |  |
| H3.bin.28 | 0 | 48.3602 | 591.062 | 0.42265 |  | 0.42265 |  | 0.45563 |  |
| H3.bin.30 | 0 | 915.626 | 3500.02 | 0.09214 |  | 0.35599 |  | 0.47243 |  |
| H3.bin.34 | 0 | 13576.7 | 4751.14 | 0.17479 |  | 0.25056 |  | 0.31436 |  |
| H3.bin.36 | 0 | 837.113 | 1248.55 | 0.18428 |  | 0.30967 |  | 0.71455 |  |
| H3.bin.39 | 0 | 380.919 | 968.368 | 0.02311 | * | 0.28292 |  | 0.47079 |  |
| H3.bin.4 | 0 | 662.739 | 1460.79 | 0.18895 |  | 0.42265 |  | 0.64319 |  |
| H3.bin.43 | 253.172 | 2949.7 | 3483.79 | 0.01598 | * | 0.06361 |  | 0.60884 |  |
| H3.bin.45 | 0 | 2422.2 | 4793.37 | 0.18069 |  | 0.01673 | * | 0.17736 |  |
| H3.bin.50 | 0 | 0 | 4348.96 |  |  | 0.36072 |  | 0.36072 |  |
| H3.bin.53 | 0 | 297.777 | 2910.4 | 0.18723 |  | 0.29641 |  | 0.33551 |  |
| H3.bin.54 | 0 | 168.915 | 796.155 | 0.18551 |  | 0.25869 |  | 0.3431 |  |
| H3.bin.56 | 0 | 851.27 | 1352.29 | 0.06922 |  | 0.21993 |  | 0.58715 |  |
| H3.bin.57 | 0 | 573.708 | 1239.84 | 0.42265 |  | 0.42265 |  | 0.66122 |  |
| H3.bin.6 | 0 | 691.924 | 1361.86 | 0.19434 |  | 0.27818 |  | 0.55432 |  |
| H3.bin.60 | 0 | 2506.79 | 4138.88 | 0.00495 | ** | 0.19693 |  | 0.53103 |  |
| H3.bin.61 | 0 | 0 | 1230.18 |  |  | 0.32071 |  | 0.32071 |  |
| H3.bin.62 | 0 | 0 | 1093.23 |  |  | 0.42265 |  | 0.42265 |  |
| H3.bin.65 | 0 | 4492.94 | 3461.43 | 0.03527 | * | 0.16458 |  | 0.61131 |  |
| H3.bin.68 | 1084.61 | 451.164 | 1149.41 | 0.01501 | * | 0.93181 |  | 0.40658 |  |
| H3.bin.7 | 0 | 9777.23 | 15905.4 | 0.19738 |  | 0.09318 |  | 0.45018 |  |
| H3.bin.70 | 0 | 1519.6 | 1264.71 | 0.14505 |  | 0.32113 |  | 0.83914 |  |
| H3.bin.8 | 0 | 2649.2 | 9823.96 | 0.38873 |  | 0.23626 |  | 0.3501 |  |
| H3.bin.81 | 0 | 2218.7 | 1534.16 | 0.02776 | * | 0.24177 |  | 0.55122 |  |
| H3.bin.83 | 0 | 3455.63 | 4369.95 | 0.12555 |  | 0.0682 |  | 0.64088 |  |
| H3.bin.84 | 0 | 1083.75 | 2408.65 | 0.00214 | ** | 0.16391 |  | 0.35752 |  |
| H3.bin.85 | 0 | 147.436 | 5030.48 | 0.42265 |  | 0.15144 |  | 0.15806 |  |
| H3.bin.88 | 421.279 | 4310.77 | 9782.29 | 0.18928 |  | 0.14332 |  | 0.30852 |  |
| H3.bin.89 | 0 | 89.6358 | 1107.73 | 0.42265 |  | 0.28303 |  | 0.31254 |  |
| H3.bin.90 | 0 | 197.172 | 1673.38 | 0.09992 |  | 0.3667 |  | 0.4147 |  |
| H3.bin.92 | 0 | 0 | 1985.28 |  |  | 0.42265 |  | 0.42265 |  |
| H3.bin.95 | 0 | 0 | 706.544 |  |  | 0.42265 |  | 0.42265 |  |
| H3.bin.97 | 0 | 0 | 713.029 |  |  | 0.42265 |  | 0.42265 |  |
