# Supplementary material for: Effects of diarrhea and antibiotic-induced microbial elimination on dynamic changes in fecal microbial communities and antibiotic resistance of Hu sheep lambs (Ovis aries)
Source: PeerJ. 2026 Jul 31;14:e21574. doi: 10.7717/peerj.21574 (PMC13431306; doi:10.7717/peerj.21574)
Supplement: Supplemental Information 6 [file peerj-14-21574-s006.docx]

| Supplementary Table S5. The main ARG family in MAGs. | |
| --- | --- |
| ARG type | Number |
| AAC(6')-Iad | 29 |
| AAC6_Ie_APH2_Ia | 1 |
| aadA | 1 |
| abeS | 3 |
| acrB | 4 |
| adeF | 1 |
| Afab_ACT_CHL | 1 |
| amrA | 1 |
| ANT(6)-Ib | 1 |
| APH(2'')-IIa | 1 |
| arlR | 24 |
| arlS | 2 |
| arnA | 3 |
| AxyY | 1 |
| bacA | 3 |
| Bado_rpoB_RIF | 9 |
| Bbif_ileS_MUP | 1 |
| bcrA | 14 |
| catB3 | 1 |
| catB8 | 1 |
| catQ | 2 |
| catS | 1 |
| CblA-1 | 1 |
| Ccol_ACT_CHL | 2 |
| CdnimB | 1 |
| cepA | 3 |
| CepA-44 | 1 |
| CfxA2 | 12 |
| CfxA6 | 2 |
| cmeB | 6 |
| cmlA5 | 1 |
| CRP | 3 |
| dfrA3 | 1 |
| dfrA35 | 1 |
| dfrF | 7 |
| dfrI | 1 |
| Ecol_acrA | 1 |
| Ecol_emrE | 2 |
| efrA | 25 |
| efrB | 14 |
| Erm(42) | 1 |
| ErmB | 1 |
| ErmQ | 1 |
| hp1181 | 1 |
| IreK | 1 |
| kdpE | 2 |
| LlmA_23S_CLI | 262 |
| lmrD | 2 |
| lnuA | 1 |
| lnuC | 9 |
| lnuD | 2 |
| lnuE | 1 |
| LnuP | 1 |
| lsaB | 8 |
| lsaE | 73 |
| macB | 48 |
| MCR-8.1 | 1 |
| mdtA | 1 |
| mdtC | 1 |
| Mef(En2) | 1 |
| mel | 3 |
| MexA | 1 |
| MexB | 2 |
| MexE | 1 |
| MexF | 3 |
| msbA | 8 |
| mtrD | 1 |
| MUN-1 | 1 |
| MuxB | 1 |
| nimA | 5 |
| nimB | 5 |
| nimE | 1 |
| nimF | 2 |
| nimG | 6 |
| nimH | 1 |
| nimI | 3 |
| nimJ | 10 |
| novA | 12 |
| optrA | 5 |
| otr(A)S.liv | 1 |
| otr(A)S.rim | 5 |
| patA | 2 |
| patB | 7 |
| PAU-1 | 1 |
| PmrF | 2 |
| poxtA | 3 |
| qacJ | 1 |
| qacL | 4 |
| RanA | 75 |
| RanB | 15 |
| rosA | 10 |
| rpoB2 | 210 |
| rsmA | 11 |
| Saur_mupA_MUP | 12 |
| Saur_mupB_MUP | 6 |
| smeA | 1 |
| smeF | 1 |
| sul4 | 1 |
| TaeA | 9 |
| tet(32) | 4 |
| tet(35) | 1 |
| tet(40) | 2 |
| tet(44) | 2 |
| tet(Q) | 4 |
| tet(S) | 1 |
| tet(T) | 2 |
| tet(W) | 1 |
| tet(W/32/O) | 1 |
| tet(W/N/W) | 1 |
| tetA(58) | 1 |
| tetA(60) | 1 |
| tetB(46) | 1 |
| tetB(60) | 4 |
| tetB(P) | 2 |
| tva(A) | 1 |
| ugd | 136 |
| vanG | 18 |
| Vang_ACT_CHL | 1 |
| vanI | 1 |
| vanR_in_vanA_cl | 9 |
| vanR_in_vanB_cl | 1 |
| vanR_in_vanD_cl | 2 |
| vanR_in_vanF_cl | 3 |
| vanR_in_vanG_cl | 29 |
| vanR_in_vanI_cl | 15 |
| vanR_in_vanP_cl | 8 |
| vanS_in_vanA_cl | 6 |
| vanS_in_vanC_cl | 2 |
| vanS_in_vanG_cl | 7 |
| vanS_in_vanL_cl | 1 |
| vanT_in_vanG_cl | 5 |
| vanTr_in_vanL | 1 |
| vanU_in_vanG_cl | 53 |
| vanW_in_vanB_cl | 1 |
| vanW_in_vanG_cl | 1 |
| vanX_in_vanP_cl | 1 |
| vanXY_in_vanG | 4 |
| vanY_in_vanA_cl | 1 |
| vanY_in_vanB_cl | 12 |
| vanY_in_vanG_cl | 2 |
| vatA | 3 |
| vatB | 24 |
| vatE | 3 |
| vatF | 7 |
| YajC | 1 |
| ykkD | 1 |
| total | 1443 |
